# Systematic review on the association between brain-related measures and upper limb movement characteristics after stroke

**DOI:** 10.1186/s12984-026-02094-4

**Published:** 2026-08-08

**Authors:** Anne Schwarz, Min-Keun Song, Ming Wu, Shivani Sakthi, Maeve Settle, Eden Farahmand, Rachel Wing, Matthew Hansen, Janne Marieke Veerbeek, Steven C. Cramer

**Affiliations:** 1 Department of Neurology, David Geffen School of Medicine at UCLA, 710 Westwood Plaza, Reed C239, Los Angeles, CA 90095-1769 USA; 2 California Rehabilitation Institute, Los Angeles, CA USA; 3 Department of Physical and Rehabilitation Medicine, Chonnam National University Medical School, Gwangju, Republic of Korea; 4 Department of Medicine, Rutgers Robert Wood Johnson Medical School, New Brunswick, NJ USA; 5 Clinic for Neurology and Neurorehabilitation, Lucerne Cantonal Hospital, University Teaching and Research Hospital of the University of Lucerne, Lucerne, Switzerland; 6 School of Exercise Science, Physical and Health Education, University of Victoria, Victoria, BC Canada; 7 Institute on Aging & Lifelong Health, University of Victoria, Victoria, BC Canada

**Keywords:** Stroke, Upper extremity, Biomechanical assessment, Neuroimaging, Neurophysiology, Rehabilitation

## Abstract

**Background:**

Upper limb (UL) movements are highly diverse, as are movement deficits following stroke. The relationship that stroke pathology has with UL impairments and their recovery has been studied with an increasingly complex array of neuroimaging and neurophysiology tools. This systematic review summarizes evidence on brain-related measures and UL movement in subjects with stroke.

**Methods:**

Bibliographic databases were systematically searched for combinations of ‘brain function’ and ‘kinematics/ kinetics/ muscle activity’ and ‘stroke’ and ‘upper limb’. Studies were included if both brain and UL movement measures were assessed in at least 10 subjects. Each study was evaluated for risk of bias (adapted QUADAS-2 tool). Information on assessments for brain and UL measures, their associations, and experimental tasks was extracted, summarized, and synthesized. This study was registered on PROSPERO (CRD42025637373).

**Results:**

A total of 220 studies were included that reported 288 experimental tasks across 4'954 subjects with stroke (mean 59.7 years). Transcranial magnetic stimulation, functional magnetic resonance imaging (fMRI), and electroencephalography, commonly combined with grip force sensors and motion sensor- or camera-based systems, were the most frequently used methodologies. Overall, 92 brain-related and 201 UL metrics were assessed. Risk of bias was low-to-moderate. Structural brain measures revealed associations between corticospinal tract integrity and greater grip strength, as well as kinematic differences between left- and right-hemisphere stroke locations. For functional brain measures, increased activation of ipsilesional motor areas during fMRI was associated with improved smoothness. In addition, studies reported reduced activation intensity and excitability after stroke, alongside task-related recruitment of additional brain areas.

**Discussion:**

This review summarizes a large and heterogeneous body of research reflecting substantial methodological and technological advances in the assessment of brain and UL movement measures after stroke. The current findings support the relevance and utility of combining brain-based and UL metrics to identify meaningful associations between neurological injury, brain function, and behavioral outcomes after stroke and thereby provide an improved understanding of movement after stroke.

**Supplementary Information:**

The online version contains supplementary material available at https://doi.org/10.1186/s12984-026-02094-4.

## Introduction

The desire to understand the neural control of arm and hand movement in humans has motivated extensive study, especially when these are affected by stroke, which is commonly associated with upper limb (UL) motor deficits that limit independence in daily living [1]. Using the UL to reach for an object in space requires efficient processing of multisensory information related to the object (e.g., size, texture, recognition), the environment (e.g., spatial location), and the individual. In parallel, appropriate motor programs (e.g., muscle combinations) and grasp configurations must be selected and executed to enable successful interaction with the object or environment. The underlying processing and control mechanisms are believed to be arranged in several small- and large-scale sensorimotor loops with convergent and overlapping brain networks, such as frontoparietal, cortico-striatal, cortico-thalamic, or cortico-cerebellar loops [2, 3]. How these small- and large-scale central nervous system loops are orchestrated to control the wide range of UL movements remains largely unknown; however, it is generally assumed that the relative cortical and subcortical control varies with task demands.

In this context, stroke can affect these sensorimotor loops in numerous ways, leading to many forms of motor deficit, such as weakness, impaired coordination, and a loss of dexterity [4, 5], with substantial interindividual variability in severity and recovery. The neuropathological mechanisms underlying these deficits remain incompletely understood. Damage to the corticospinal tract (CST), assessed structurally using neuroimaging or functionally using Transcranial Magnetic Stimulation (TMS), is consistently associated with UL impairment and recovery across studies and methodological approaches. Accordingly, CST integrity is currently considered the primary prognostic biomarker for UL recovery after stroke [6], meaning it is used to predict recovery potential and stratify patients; however, these measures typically explain only ~ 20% of the inter-individual variability in outcomes [7]. Beyond CST damage, numerous additional brain-related measures, across many anatomical regions, have been implicated in UL motor impairment and recovery, e.g., injury to secondary motor cortical and subcortical regions [8], brain age using structural magnetic resonance imaging (MRI) [9], interhemispheric functional connectivity based on electroencephalogram (EEG) [10], and functional disconnections between motor and cognitive networks in the prefrontal cortex measured with functional MRI (fMRI) [11]. Together, these approaches span multiple spatial and temporal scales and reflect complementary aspects of stroke-related alterations in brain structure and function. While neuroimaging techniques such as MRI and fMRI provide spatially resolved measures of brain structure and activity, neurophysiological methods, including EEG and TMS, offer complementary information on cortical dynamics, functional connectivity, and corticospinal excitability. Accordingly, this review examines the full range of brain-related measures used to characterize stroke-related brain injury and its consequences for functioning.

Equally important to characterizing brain injury is the precise definition and measurement of behavior. Detailed kinematic and kinetic analysis of UL movements have been recommended in stroke research because they provide accurate and reliable quantification of motor behavior [12], and allow differentiation between movement restitution and compensation [13]. These measures complement clinical scales [14] and self-reported outcomes, [15] and permit fine-grained evaluation of neuromodulatory interventions [16]. Rapid technological advances, including high-resolution imaging, motion analysis systems (e.g., cameras, wearable sensors), and robotic technologies, have facilitated these approaches [17], yet the growing number and diversity of devices make it challenging to meaningfully synthesize the current evidence.

Although numerous narrative expert reviews exist in this field [2, 3, 12, 18–20], these are not systematically conducted and are therefore vulnerable to bias. Other reviews employing systematic search strategies have focused either on one specific brain measurement system, such as fMRI or EEG [21], or on broader assessments of motor functions across many body parts [22, 23].

The goal of the current systematic review was to summarize the state of the art of research on the associations that measures of brain structure and function have with UL movement kinematics, kinetics, and muscle activity after stroke. Specifically, we addressed the following questions: 1) which brain structures, connections, and activations; and which kinematic, kinetic, and muscle activity metrics have been investigated with respect to UL motor functioning after stroke; 2) what associations have been reported between brain-related and UL movement-related measures in subjects with stroke; 3) how do brain-related and UL movement-related measures differ between subjects with stroke and healthy controls (e.g., in their expression or in the strength and nature of reported associations); and 4) how do brain-related and UL movement-related measures vary as a function of the type of UL movement task assessed (e.g., single-joint isometric strength test versus a multi-joint grasp task or tasks with increased motor or cognitive complexity). Answering these questions will help to structure the rapidly growing literature, identify consistent and complementary brain-behavior relationships, uncover gaps in current research, and guide the selection of brain and UL movement measures for future mechanistic and interventional studies in stroke recovery.

## Methods

This systematic review was performed and written in accordance with the Preferred Reporting Items for Systematic Reviews and Meta-Analysis requirements [24] and registered in the international prospective register of systematic reviews (PROSPERO: CRD42025637373). The search was performed by two authors (A.S. and J.M.V.) in PubMed, Embase, and NeuroImage journal from inception to June 9, 2023 by searching based on combinations of key terms of “brain”, “upper extremity”, “kinematics”, “kinetics”, "muscle activity", and “stroke” and is reported in the supplementary material (SM Section 1e, Box 1 and Box 2).

Studies were included if they had at least 10 adult (≥ 18 years old) participants, stroke survivors with or without healthy control subjects; were published in a peer-reviewed journal; and reported measures of brain structure and/or function, as well as UL kinematic, kinetics and/or muscle activity within an experimental design involving active UL movement. Articles were considered in English language. Studies that solely focused on movement intention detection (e.g., for device control) and studies that did not include active movements (e.g., motor imagery, passive movements, or purely sensory assessments) were excluded (for complete eligibility see SM Section 1b). Researchers were not blinded regarding study authors and groups.

### Data collection and definitions

Information about brain-related and UL measurements, the timing of brain assessments relative to UL measures (simultaneous or not), study design, participant demographics including stroke characteristics when appropriate (e.g., time post-stroke, motor impairment severity), and study findings were extracted from each included study.

The full range of brain-related measurement systems was considered including structural (e.g., computed tomography [CT] and/or MRI [diffusion-weighted and others]) and functional (e.g., fMRI, EEG, and TMS) measures. Common examples for structural metrics are stroke lesion location and volume, grey and white matter volumes, or white matter tract characteristics, and frequently reported functional metrics include blood oxygenation level dependent (BOLD) contrasts based on fMRI, as well as EEG-based spectral analysis and event-related potentials (ERPs). The corresponding definitions of each brain-related metric was extracted to assure their interpretability. In stroke subjects, the side of the brain was defined as ipsilesional (IL) for the lesioned hemisphere and contralesional (CL) for the non-lesioned hemisphere. In addition, the reported brain regions of interest (ROI) were extracted together with direct neural effects associated with any intervention, which were classified as positive (e.g., facilitation/activation), negative (e.g., inhibition/damage) or neutral. The rationale for this data collection was to synthesize and quantify reported neural findings across brain ROIs, for example by determining how many studies addressed subcortical lesions, facilitation of the IL primary motor cortex (M1), or inhibition of the CL M1.

UL kinematic and kinetic measures included characteristics of motion, forces, torques, and muscle activation related to the shoulder, arm, hand, and fingers, as captured by electromyography (EMG), force sensors, camera- and/or sensor-based systems. Measurement systems were categorized into minimal (e.g., sensor-based or camera-based systems), medium (e.g., end-effector systems) and high (e.g., exoskeletons or splints with weight support) influence groups based on their presumed level of interaction forces with the person being assessed in accordance with a preceding review [25]. Their metrics were assigned to one of the following categories: EMG, force, accuracy, data-driven scores, efficacy, efficiency, movement planning, precision, smoothness, spatial posture, speed, or temporal posture [13, 14, 25]. A list of movement category definitions appears in the supplemental material (SM Section 1a). EMG measures used exclusively to quantify TMS-evoked motor evoked potentials (MEPs) were classified separately and were therefore excluded from the UL movement-related metrics.

Besides the brain-related and UL movement related metrics, the experimental movement task that formed the basis of the assessment was recorded and assigned to one of five task groups that reflect different domain in the International Classification of Functioning, Disability and Health (ICF) framework [26]: for *body functions and structures*, tasks were assigned to *strength tasks* (e.g., grip strength), or *active range of motion (aROM) tasks* (e.g., wrist flexion–extension); and *activities*, which were subclassified as *reach tasks* (e.g., planar pointing), *grasp tasks* (e.g., 3D reach-to-grasp), or *complex tasks* that involve high demands on motor and/or cognitive functioning (e.g., motor sequence learning, choice reaction time, dual task designs [27]). Importantly, *grasp* tasks were defined as comprising distinct phases, including a reaching phase with grasp preparation, object contact and manipulation, in contrast to grip *strength* tasks, which involve unidimensional isometric movements. Some studies had several different experimental movement tasks, with each listed separately.

### Study quality assessment

The risk of bias for the included studies was assessed with an adapted version of the Quality Assessment of Diagnostic Accuracy Studies [28, 29] (QUADAS-2), a tool to evaluate a diagnostic study’s quality by means of the population tested, the diagnostic test of interest, the gold standard reference test, and the timing between both tests. The adaptation of QUADAS-2 involved the inclusion of a second index test category that allows to rate both brain-related measures (index 1) and UL movement-related measures (index 2) with respect to clinical scales as reference standard. The adapted version that was used to score the QUADAS-2 tool consisted of the participant selection section (e.g., group balance, sample size), brain-related measures as index test 1 (e.g., acquisition method, assessor blinding, results, missing values), UL movement-related measures as index test 2 (e.g., acquisition method, assessor blinding, results, missing values), clinical scales when applicable as the reference standard (e.g., measure, blinding toward index test 1 and 2), and flow and timing (e.g., timing of index tests, timing with reference measure, completeness of data) of measures [28], which resulted in a total of 18 items that were rated with “yes” if satisfied, “no” if not satisfied and supported by specifications and qualifiers per rating, or “unclear”. This adapted version was developed by two reviewers (A.S. and J.M.V.), then tested and validated by two reviewers (A.S. and M.K.S.). Additional raters were trained and approved by two reviewers (A.S. and M.K.S.), who cross-checked a convenience sample of their ratings.

### Synthesis of results

The review findings were summarized by means of observational (e.g., observational cross-sectional studies [O-C], observational longitudinal studies [O-L]) and interventional studies (e.g., interventional cross-sectional (≤ 1 day) [I-C], interventional longitudinal studies (> 1 day) [I-L]).

The extracted study data were grouped according to the study population (stroke vs. healthy controls, stroke subjects only). Participant characteristics were extracted by means of UL impairment severity, UL activity limitations assessed and summarized using median and (quartile 1—quartile 3) of the Fugl-Meyer Assessment for the Upper Extremity (FMA-UE) and the Action Research Arm Test (ARAT), respectively. Although the FMA-UE and ARAT are commonly used and closely correlated UL assessments, the FMA-UE predominantly reflects impairments on the ICF *body functions and structures* level, whereas the ARAT captures ICF *activity* level deficits [26, 30]. Time post-stroke was also extracted and grouped into acute (≤ 7 days), early subacute (≤ 3 months), late subacute (< 6 months), and chronic > 6 month based on the time at study [31].

Brain-related and UL movement-related outcome systems and metrics, timing of assessments, and investigated brain ROIs, including the reported direction of neural modulation, when available (e.g., inhibition/damage or facilitation/activation), were extracted, counted across all studies and within each task group, and summarized using Sankey diagrams and frequency plots. These visualizations illustrate both the occurrence of specific measures across studies and how these brain- and UL movement-related measures were combined within experiments, including the targeted brain regions and their reported neural modulation. Brain and UL movement metrics were considered relevant for future research if they were reported in at least five studies within one movement task group and in at least 3 of the 5 task groups overall.

Findings regarding associations between brain-related and UL movement measures were extracted and summarized for brain and UL movement metrics or category pairs that were reported in more than one study. Information on the movement task and the study population were included in subgroup analysis using the above approach to explore potential differences in brain and UL movement metrics.

## Results

**Fig. 1:**
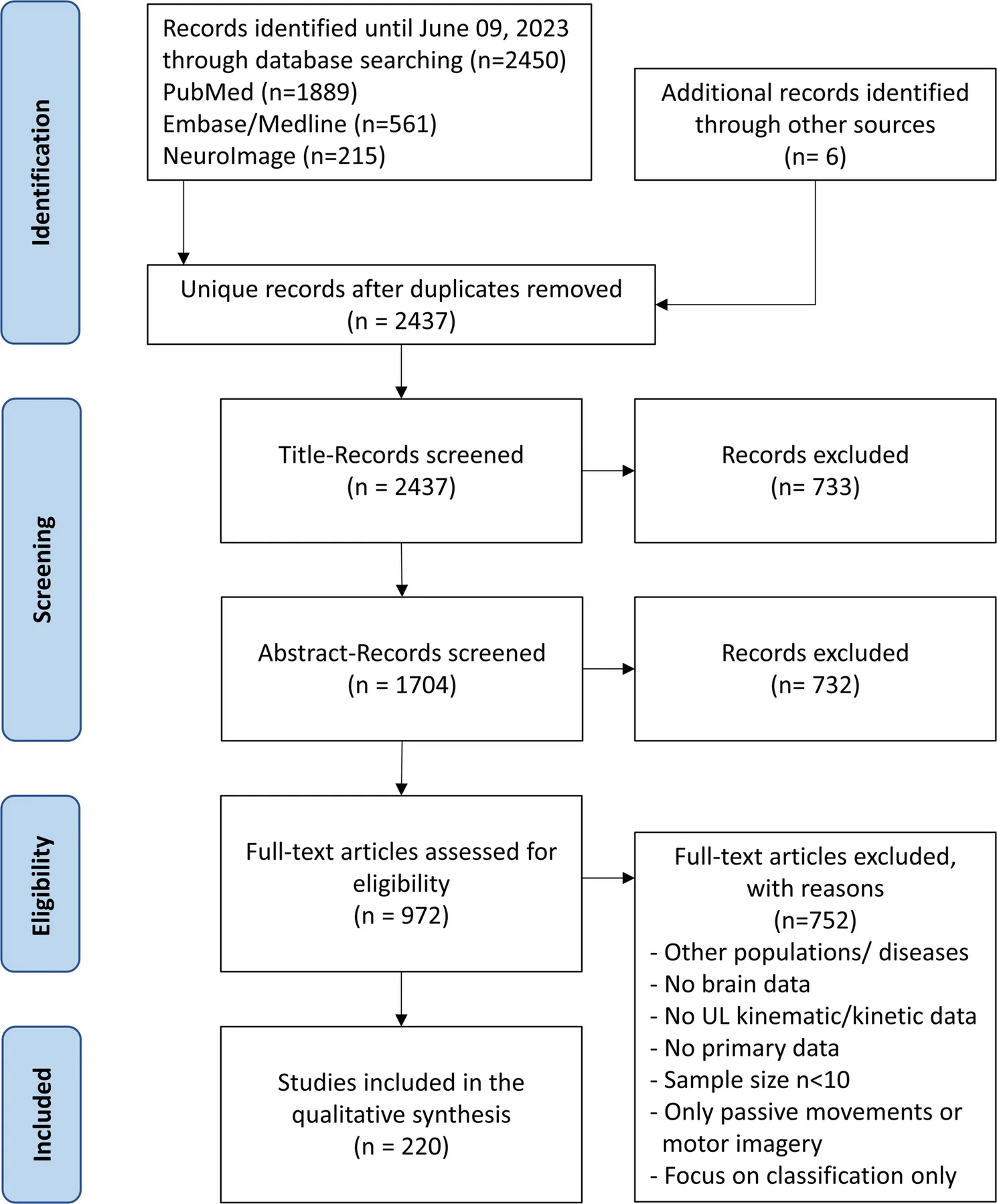
Flow chart of systematic review

A total of 220 studies [8, 32–250], comprising 4′954 stroke subjects (mean age 59.7 years, 34.1% females) and 2′709 healthy controls (mean age 56.1 years, 42.1% females), and representing 288 experimental tasks, met the inclusion criteria as shown in the flow of search and study identification in Fig. 1. Characteristics of the included studies are summarized in the supplementary material (Section 1c. SM Table 1. Study characteristics).

Comparisons between subjects with stroke and healthy controls were performed in 122 studies, including 87 cross-sectional observational, 18 longitudinal observational, 5 cross-sectional interventional, and 12 longitudinal interventional studies. The remaining 98 studies investigated subjects with stroke only and comprised 29 cross-sectional observational, 7 longitudinal observational, 12 cross-sectional interventional, and 50 longitudinal interventional studies. The longitudinal observational timeframes varied from 1–2 weeks, [101, 129, 234] over 1 month [112, 132, 133, 200], 2–3 months [58, 64, 121, 217, 222] to 6 months and more [8, 83, 88, 163, 172, 179, 230] with differing visit schedules.

Interventional studies investigated the effects of single-session interventions (n = 17) or interventions lasting 2–12 weeks (n = 62), with or without follow-up. Most of the 79 interventional studies investigated the effects of non-invasive central and peripheral neuromodulation techniques (n = 37; repetitive TMS, theta burst stimulation [TBS], transcranial direct current stimulation [tDCS], functional electrical stimulation [FES], and nerve stimulation [NS]). These were followed by exercise-based interventions (n = 11; e.g., constraint-induced movement therapy and bilateral arm training), combination therapies (n = 10; e.g., exercise combined with TMS or robotic therapy combined with tDCS), robotic therapy alone (n = 8), brain-computer interface-based interventions (n = 6), and, less frequently, pharmacological interventions (n = 2), music therapy (n = 1), monetary incentives (n = 1), acupuncture (n = 1), virtual reality (n = 1), and magnifying glasses (n = 1).

Participants were enrolled in the acute phase (until 7 days) [251] in 22 studies, the early subacute phase (1 week–3 months) in 49 studies, the late subacute (3–6 months) in 22 studies, and the chronic phase (> 6 months) after stroke in 120 studies. Three studies included participants across the combined subacute phase (1 week–6 months), and four studies did not report the timepoint since stroke. UL motor impairments and UL activity limitations were mostly moderate, as assessed by the FMA-UE (median 38.6, Q1-Q3: 26.0–48.1), reported in 84 studies (38.2%), and the ARAT (median 34.9, Q1-Q3: 25.1–44.3), reported in 39 studies (17.7%).

### Risk of bias assessment

The risk of bias assessment was overall low to moderate (median 69.44%, range 33.33–94.44). Most of the studies reported aspects of the participant selection (e.g., eligibility criteria), attempted to control for age and sex when including healthy or control groups (54.98% of studies), and included information from reference measures (e.g., FMA-UE or National Institutes of Health Stroke Scale) for characterizing the stroke population (91.04% of the studies). The study sample size was in general low, with only 36.03% of studies having 30 or more participants.

The acquisition methods and results were reported in 93.39% the brain-related and 90.64% of the UL movement-related measures. Assessor blinding towards group allocation or other outcome/reference measures was unclear in 55.81% of studies regarding brain-related measures, and 63.57% of studies regarding UL-related measures. Missing data and appropriate handling strategies were reported for brain-related measures in 25.19%, and for UL movement measures in 17.84%, of studies, while most were unclear in this regard (67.05% and 77.32%, respectively). Both brain and UL movement measures were assessed within one week of each other in 67.03% of the studies, 32.60% reported unclear information on timing. A complete overview of the risk of bias assessment can be found in the supplementary material (SM Fig. 1).

### Data synthesis

The following section presents the findings of this review, organized according to the four predefined research questions. Specifically, we summarize: 1) which brain-related and UL movement-related measures have been investigated in subjects after stroke; 2) the reported associations between brain-related and UL movement-related measures; 3) differences between subjects after stroke and healthy controls in terms of the expressions of these measures and/or their associations; and 4) differences in the expressions of measures and/or their associations across movement tasks. Each question is addressed in a separate subsection.

#### Brain-related and UL movement-related measures investigated after stroke

**Fig. 2:**
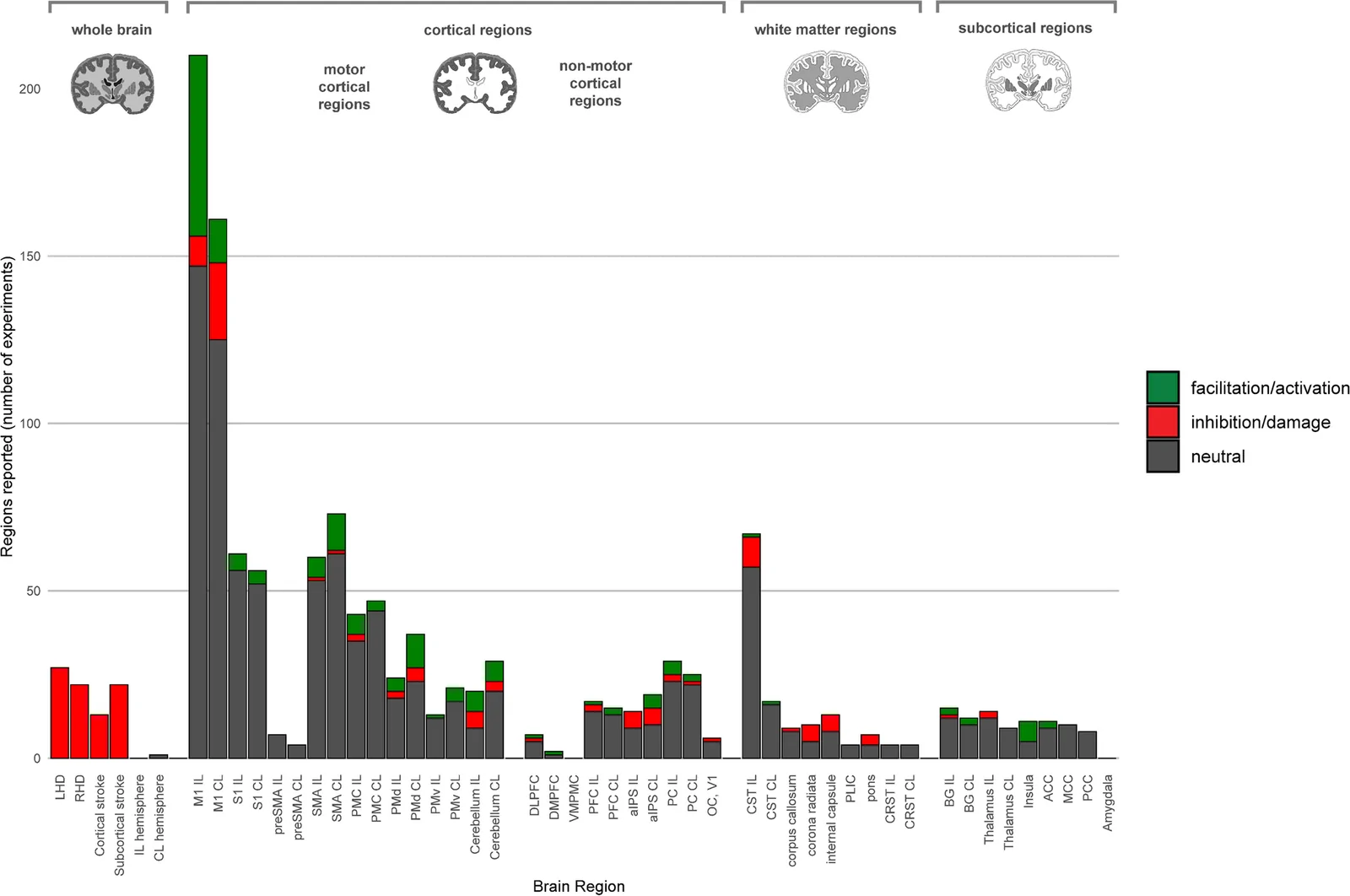
Brain regions investigated across 220 studies and 288 experiments. Green parts of the bar plot indicate experiments where the region of interest was stimulated or showed activations by means of brain-related metrics (e.g., motor evoked potentials, functional magnetic resonance imaging related contrasts), red parts indicate experiments where the considered region of interest was damaged by the stroke lesion or showed decreased activation or inhibition, and the dark grey parts refer to experiments, where a region was mentioned in the methods but not further specified or did not show significant results and was therefore considered neutral. ACC, anterior cingulate cortexf; aIPS, anterior intraparietal sulcus; BG, basal ganglia; CL, contralesional; CRST, corticoreticulospinal tract; CST, corticospinal tract; DLPFC, dorsolateral prefrontal cortex; DMPFC, dorsomedial prefrontal cortex; IL, ipsilesional; LHD, left hemispheric stroke; M1, primary motor cortex; MCC, midcingulate cortex; OC, occipital cortex; PC, parietal cortex; PCC, posterior cingulate cortex; PFC, prefrontal cortex; PLIC, posterior limb of the interal capsule, PMC, premotor cortex; PMd, dorsal premotor cortex; PMv, ventral premotor cortex; preSMA, pre-supplementary motor area; RHD, right hemispheric stroke; S1, primary sensory cortex; SMA, supplementary motor area; VMPFC, ventromedial prefrontal cortex

**Fig. 3:**
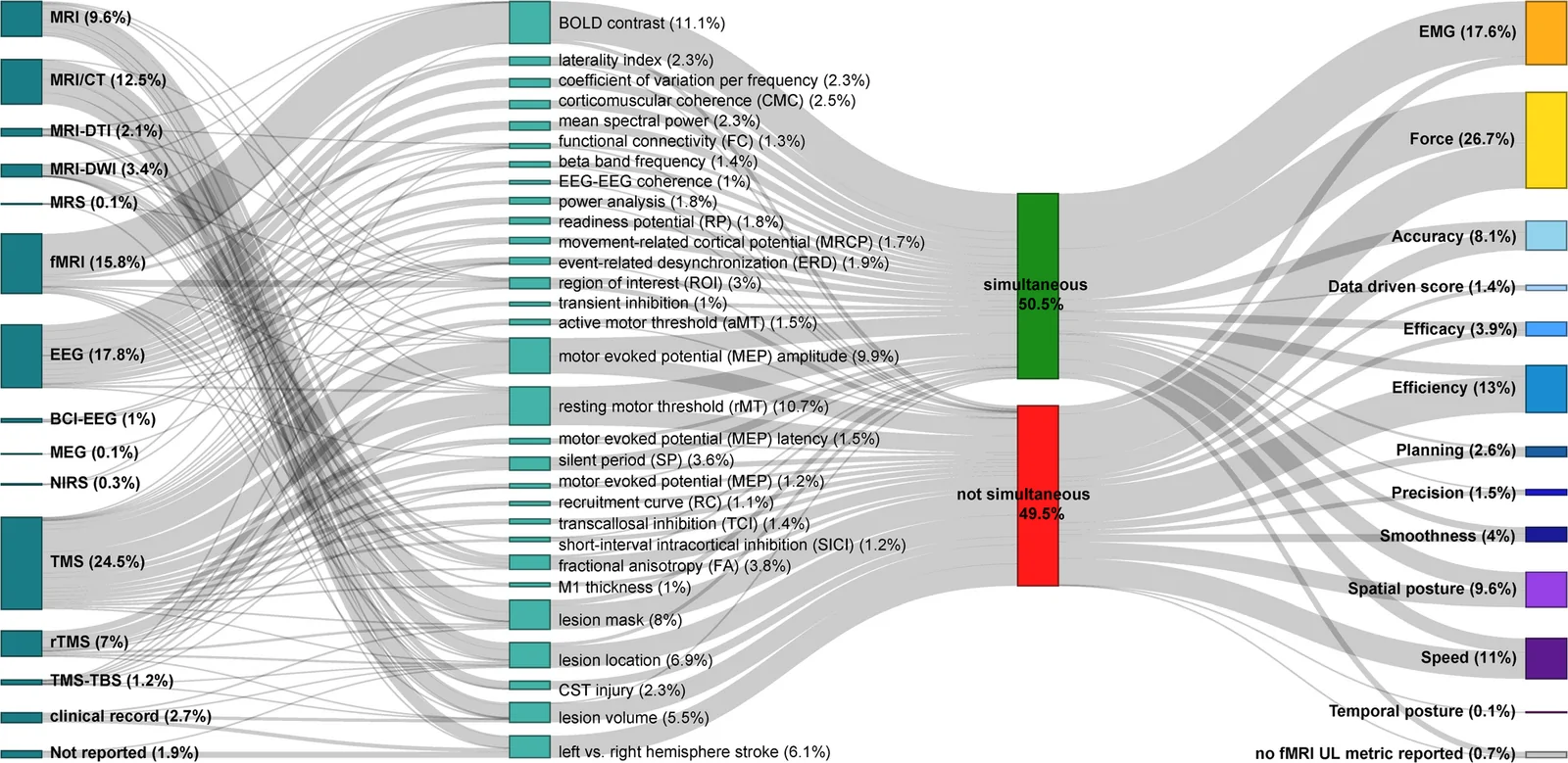
Combinations of brain-related and upper limb movement-related measures across all 220 studies and 288 experiments. BCI, brain computer interface; CST, corticospinal tract; CT, computer tomography; DTI, diffusion tensor imaging; DWI, diffusion weighted imaging; EEG, electroencephalogram; EMG, electromyography; FES, functional electrical stimulation; fMRI, functional magnetic resonance imaging; MEG, magnetencephalography; MRI, magnetic resonance imaging; MRS, magnetic resonance spectroscopy; rTMS, repetitive TMS; SPECT, single-photon emission computed tomography; TBS, theta burst stimulation; TCD, transcranial doppler sonography; tACS, transcranial alternating current stimulation; tDCS, transcranial direct current stimulation; TMS, transcranial magnetic stimulation. Connections represent co-occurrence of brain-related and UL movement-related measures within the same study. Links do not indicate that one measure was derived from another but reflect multimodal assessment within individual studies

**Fig. 4:**
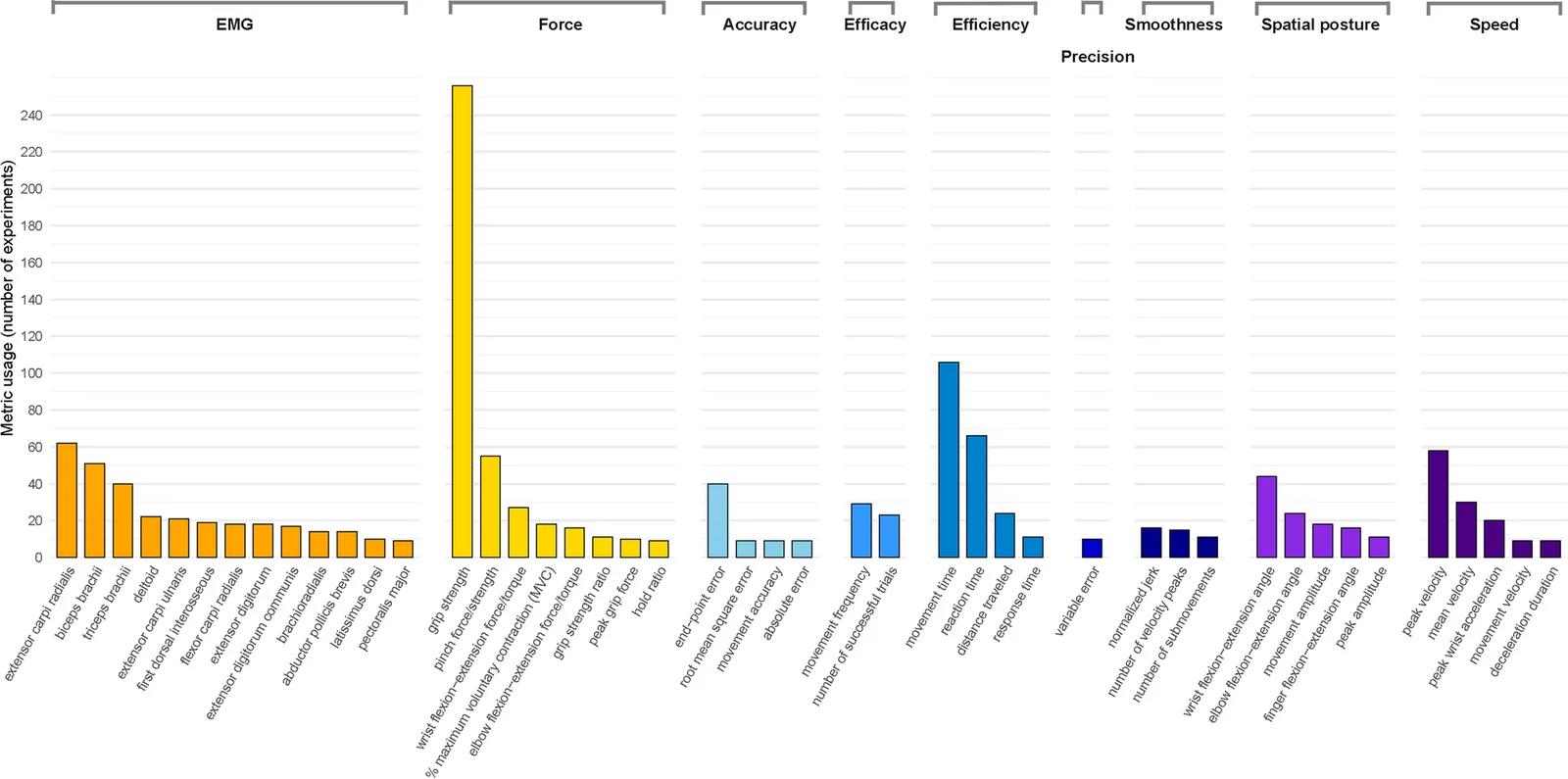
Frequency of upper limb metrics across all 220 studies and 288 experiments

Across studies, a total of 92 brain-related and 201 UL movement-related metrics, as well as 50 different brain ROIs (16 motor cortex, 10 non-motor cortex, 9 white matter, 9 subcortical, and 6 global brain regions) were identified. The main breadth of extracted brain-related and UL movement-related measures across studies is summarized in Figs. 2, 3, 4 according to the brain ROIs, brain and UL movement measurement system, associated metrics, and timing of measures. Definitions of the brain-related metrics reported in Fig. 3 are provided in Table 1. Definition of the UL movement-related metrics are reported elsewhere [25].

**Table 1:** Definition of brain-related metrics

Measurement system	Brain related metric	Definition	Relevant references
	Regions of interest (ROI)	Is a specific anatomical area or set of voxels (3D pixels) selected in neuroimaging data (like fMRI, PET, DTI) to focus analysis on a particular structure or functional network	[44, 51, 60, 63, 71, 91, 114, 127, 133, 143, 148, 168, 185, 228, 236, 248]
S MRI/CT	CST injury	Is defined as the extent of damage caused by a stroke lesion to the corticospinal tract, a major white-matter pathway that connects the primary motor cortex with the brainstem and spinal cord and transmits descending motor commands and sensorimotor signals. CST injury is usually quantified by neuroimaging (e.g., overlaying lesion masks with CST template, diffusion tensor imaging) or neurophysiological methods (e.g., assessing motor evoked potentials with transcranial magnetic stimulation)	[8, 66, 70, 91, 99, 128, 130, 150, 163, 177, 241]
	Left vs. right hemisphere stroke	Left versus right hemisphere stroke refers to a binary lesion laterality metric indicating whether the cerebral infarct or hemorrhage primarily affects the left or the right cerebral hemisphere. This metric captures hemispheric specialization in brain function and is clinically relevant because stroke laterality is associated with distinct patterns of impairment, such as language and praxis deficits following left hemisphere stroke and visuospatial, attentional, and body awareness deficits following right hemisphere stroke	[41, 67, 81, 84, 97, 111, 166, 167, 187, 188, 198, 202, 240]
	Lesion location	Refers to the anatomical position of a stroke-related brain lesion within specific cortical, subcortical, or white-matter regions. It is typically determined from neuroimaging by neuroradiological evaluation or by mapping the lesion to standardized brain atlases or regions of interest and serves as a spatial metric capturing where neural tissue is damaged	[36–38, 40, 45, 47, 49, 86, 93, 106, 115, 129, 135, 136, 146, 147, 153, 181, 186, 187, 192, 198, 210, 215, 223, 225, 233, 234, 240]
	Lesion mask	Is a binary or probabilistic neuroimaging-derived 3D reconstruction map that delineates brain tissue affected by a stroke lesion. It is created by manually, semi-automatically, or automatically segmenting the lesion on structural imaging (e.g., T1-, T2-, FLAIR-, or diffusion-weighted MRI) and is typically registered to native or standardized space	[45, 64, 70, 76, 80, 96, 120, 123, 159, 163, 221, 226, 229]
	Lesion volume	Is the quantifiable, three-dimensional space an abnormal tissue area (lesion) occupies, measured in units like cubic centimeters (cm^3^) using medical imaging (MRI, CT) to assess the damage caused by stroke	[38, 45, 83, 96, 101, 129, 139, 159, 193, 233]
S MRI	M1 thickness	Refers to the total volume of brain tissue, or grey matter volume, herein the grey matter of the primary motor cortex, primarily composed of neuronal cell bodies, dendrites, synapses, and supporting glial cells, located mainly in the cerebral cortex and subcortical nuclei. It reflects the structural capacity for local information processing and integration	[233]
S DWI/DTI	Fractional anisotropy (FA)	Is a quantitative measure from diffusion MRI (DTI) indicating the directional preference of water movement in tissue, ranging from 0 (random diffusion/isotropic) to 1 (directional diffusion/anisotropic). In the brain, high FA (near 1) signifies structured, organized white matter tracts (like myelinated fibers) where water moves along the fibers, while low FA (near 0) indicates isotropic diffusion, typical of gray matter or cerebrospinal fluid, showing uniform movement in all directions	[50, 66, 70, 83, 89, 101, 108, 139, 155, 168, 177–179, 190, 191, 199, 204, 211]
F EEG	Beta band frequency	Is a brainwave rhythm, typically 12-30 Hz (or low [12.5–16 Hz], and high beta [16-30 Hz]) most prominent in the frontal cortex (e.g., motor cortex), whereas alpha band frequency is prominent in relaxed and wakeful states with the eyes closed	[46, 141, 156]
	Corticomuscular coherence (CMC)	is a neurophysiological measure showing the functional synchronization between the brain's motor cortex and contracting muscles, indicating the efficiency of neural communication in the motor system, typically measured by correlating EEG/MEG (brain) and EMG (muscle) signals in specific frequency bands (especially beta, 15–30 Hz) during movement. It estimates quantify the strength of the coupling between cortex and the muscles (CMC)	[132, 244]
	EEG-EEG coherence	Measures the synchronization, or functional connectivity, between electrical signals from different brain areas by assessing the consistency of their phase and amplitude in specific frequency bands, indicating how well those regions are "talking" to each other, with values from 0 (no relation) to 1 (identical)	[58, 141, 156, 160, 201]
	Event-related desynchronization (ERD)	Describe relative changes in the power of specific EEG frequency bands in response to an event, where ERD (negative values) reflect cortical activation and ERS (positive values) reflect cortical inhibition or idling	[34, 55, 58, 59, 103, 144, 154, 162, 245]
	Movement-related cortical potential (MRCP)	Is a slow, low-frequency electrical signal detected by scalp EEG, appearing up to two seconds before voluntary movement, reflecting the brain's motor planning and preparation processes in areas like the supplementary motor cortex	[43, 73, 110, 149]
	Power analysis	Is the time–frequency decomposition of the sources, often performed using a Morlet wavelet transform spanning the frequency range from 5 to 48 Hz with a constant wavelet cycle number of 5 and a linear scale of 1 Hz between frequencies	[55, 59, 79, 100, 133, 154, 168, 238]
	Coefficient of variation per frequency	Is a normalized measure of variability that quantifies how much the power (or another frequency-domain metric) fluctuates relative to its mean within a specific frequency or frequency band	[154, 164]
	Mean spectral power	Is defined as the average of the power spectral density (PSD) values across frequencies within a predefined band (e.g., alpha 8–12 Hz, beta 13–30 Hz) and, typically, across time and/or trials	[164]
	Readiness potential (RP)	Or Bereitschaftspotential, is a slow, negative electrical shift in the EEG that appears in the brain seconds before a self-initiated movement, reflecting neural activity in motor areas preparing for action and is considered the first part of an MRCP	[149, 151]
F EEG, fMRI	Laterality index	Is a quantitative measure used in relation with different structural and functional brain-related measurement systems, such as MRI, fMRI and EEG, to describe the hemispheric dominance or asymmetry of brain activity during a task or at rest	[57, 127, 151, 169, 214, 238]
	Functional connectivity	Refers to statistical dependencies or temporal correlations between spatially distinct neural signals, such as BOLD signal fluctuations across brain regions based on fMRI or electrophysiological signals based on EEG, that are measured without assuming a direct causal relationship	[42, 48, 51, 131, 156, 179, 220, 221]
F fMRI	BOLD contrast	Blood oxygen lLevel dependent (BOLD) contrast MRI is the core technique in functional MRI (fMRI) that detects brain activity by measuring changes in blood oxygenation, relying on deoxyhemoglobin's magnetic properties; active brain areas receive more oxygenated blood, diluting paramagnetic deoxyhemoglobin, which increases the MRI signal and maps brain function non-invasively	[38, 51, 56, 57, 76, 78, 88, 94, 95, 119, 127, 134, 138, 142, 145, 155, 157, 170–172, 178, 182, 194–197, 205, 207, 216, 218, 222, 225, 228–230]
F TMS	Active motor threshold (aMT)	Is the minimum intensity of a TMS pulse needed to evoke a measurable MEP in a specific muscle while that muscle is actively contracting. It's a key measure of motor cortex excitability, typically set lower than the resting motor threshold (rMT) because the brain is more responsive during voluntary muscle activity, and helps researchers determine optimal stimulation levels for studies involving movement or muscle activation	[54, 69, 125, 160, 175, 183, 184, 206, 212]
	Motor evoked potential (MEP) presence	Reflects the excitability and integrity of the corticospinal system, usually indicated as presence or absence based on the EMG response evoked by TMS over the contralateral primary motor cortex (M1)	[39, 82, 129, 180, 200, 206, 242]
	Motor evoked potential (MEP) amplitude	Is strongly related to cortical output strength and synaptic efficacy and it is the peak-to-peak height of the electrical signal recorded from a muscle after stimulating the motor cortex, representing the strength or intensity of the corticospinal pathway's response, typically measured in millivolts (mV) on an EMG waveform. It's a key measure of how excitable the brain's motor system is, reflecting the overall signal strength traveling from the cortex down to the muscle	[32, 33, 36, 50, 54, 64, 112, 114, 117, 118, 120, 126, 129, 135, 140, 152, 158, 165, 175, 200, 208, 210–213, 232, 242, 246]
	Motor evoked potential (MEP) duration	Is the temporal length of the EMG response, usually measured from MEP onset to offset. It reflects the temporal dispersion and synchronization of corticospinal volleys and motor unit recruitment, and is influenced by cortical excitability, spinal motor neuron properties, and inhibitory-excitatory balance	[158]
	Motor evoked potential (MEP) latency	Is the time delay between stimulating the brain's motor cortex (e.g., TMS) and the first electrical response detected in a target muscle via EMG	[87, 117, 136, 200, 212, 219]
	Recruitment curve (RC)	It plots MEP size vs. stimulation intensity to assess nervous system function	[112, 140, 184]
	Resting motor threshold (rMT)	Was defined as the stimulus intensity needed to produce MEPs with a size of 50–100 mV in 5 out of 10 consecutive trials during complete muscle relaxation. defined as the lowest stimulus intensity necessary to produce MEPs of ≥ 50 V peak-to-peak amplitude in 5 of 10 subsequent trials	[36, 50, 54, 65, 69, 82, 100, 102, 114, 117, 118, 120, 126, 129, 135–137, 140, 143, 149, 160, 175, 180, 183, 184, 200, 206, 209–211, 217, 226, 227, 243, 245, 246]
	Short-interval intracortical inhibition (SICI)	Is a paired-pulse TMS measure of intracortical inhibitory function within the primary motor cortex. It is assessed by delivering a subthreshold conditioning stimulus followed, after a short interstimulus interval (typically 1–5 ms), by a suprathreshold test stimulus, resulting in a reduction of the motor-evoked potential (MEP) amplitude compared with the test stimulus alone. It is mediated by GABA_A_-ergic interneuronal circuits and widely used to quantify alterations in cortical inhibition, including changes associated with stroke recovery	[50, 74, 125, 204]
	Silent period (SP)	A temporary cessation of ongoing muscle activity (EMG) in a voluntarily contracting muscle, occurring after a TMS pulse to the motor cortex. It reflects the brain's inhibitory control, lasting up to a second, and is quantified by its duration, with longer periods indicating stronger cortical inhibition. It has been called cortical SP, ipsilesional and contralesional SP	[39, 54, 92, 96, 112, 120, 136, 140, 160, 183, 184, 204]
	TMS map area	TMS map area was quantified as the cortical surface area comprising all stimulation sites that produced MEP amplitudes exceeding 500 µV	[76, 129, 184, 203, 212]
	Transcallosal inhibition (TCI)	Refers to inhibitory interactions between the two cerebral hemispheres mediated by transcallosal fibers of the corpus callosum. In TMS studies, TCI is typically assessed by stimulating one motor cortex and measuring the resulting suppression of motor-evoked potentials or voluntary muscle activity in the homologous muscles controlled by the opposite hemisphere	[112, 152, 183, 204]
	Transient inhibition	Refers to the temporary suppression or disruption of neural activity in a targeted brain region induced by TMS, effectively creating a virtual lesion. This approach is used to causally probe the functional contribution of a cortical or subcortical region by examining how its brief disruption affects behavior or neural processing	[91, 94]

Of the brain measures, most studies included TMS (n = 61), followed by fMRI (n = 54), EEG (n = 34), MRI (n = 30), rTMS or TMS-TBS (n = 22), MRI/CT (n = 23), and MRI-DTI or MRI-DWI (n = 22), with a total of 92 unique brain-related metrics (18 structural, 70 functional, and 4 structural or functional metrics).

The most frequently and consistently reported structural brain-related metrics were lesion location (12.15%), the creation of a lesion mask (11.46%), lesion volume (7.29%), hemispheric side of stroke (7.29%), use of an ROI (7.29%), and CST injury (4.17%), related to MRI or CT; and fractional anisotropy (FA, 6.60%), related to MRI diffusion-weighted imaging (DWI).

The most frequently and consistently reported functional brain-related metrics across study experiments were BOLD contrast (17.36% of the experiments) related to fMRI; resting motor threshold (rMT, 18.06%), MEP amplitude (14.24%), and silent period (SP) (4.17%) related to TMS; corticomuscular coherence (CMC, 2.78%) and movement-related cortical potential (MRCP, 1.39%), related to EEG.

The UL movement-related measures were captured with systems that generally introduced minimal interference on behavior in 51% of experimental tasks, including EMG (n = 42), motion sensor-based systems (n = 30), button press/touch screen (n = 21), and camera-based systems (n = 26). The UL measures were captured with systems that introduced medium interference in 36.46% of experiments, including end-effector robots (n = 18) or dynamometers (n = 52). The UL measures were captured with systems that introduced a high level of interference in only 5.9% of experiments, such as exoskeletons (n = 18). A total of 201 individual UL metrics were identified. Amongst force-related outcomes, grip strength (36.13%) and pinch strength (9.49%) were most frequently reported. For efficiency, movement time (20.07%) and reaction time (9.49%) predominated, while elbow flexion–extension angle (2.55%) was the most commonly reported spatial position metric. Speed-related outcome most often included peak velocity (14.23%) and mean velocity (4.01%). Of the 54 fMRI studies, 11 did not report any UL movement-related measure directly related to the fMRI paradigm (0.7%), as indicated in the bottom right of Fig. 3. Brain- and UL movement-related measures were assessed simultaneously in 50.5%, and not simultaneously in 49.5%.

#### Associations between brain-related and UL movement-related measures

Each study was screened for explicitly reported statistical associations or direct comparisons between brain-related and UL movement-related measures. While several studies assessed multiple combinations of brain and UL movement-related measures within more than one experiment, a direct relationship between these measures was examined and reported in 31.14% of the included studies. Table 2 summarizes pairs of brain-related and UL movement-related metrics that were investigated in more than one study, together with their reported associations (e.g., correlation or regression analyses, specialized analyses [e.g., voxel lesion symptom mapping]) when available, and the corresponding references, organized according to study design (O-C, O-L, I-C, and I-L).

**Table 2:** Association between brain-related measures and UL movement-related measures

Brain related		UL movement related		Task Group	Association	References			
Category	Metric	Metric	Category			O-C	O-L	I-C	I-L
SMRI	CST injury	Grip strength	Force	Strength; grasp	CST LL—6-month grip and pinch control r = 0.34 [163]; CST LL _manual_—motor deficit r = 0.46, CST LL _automatic_—motor deficit r = 0.56 [123]; CST injury classified motor recovery with 73% [8]	[123, 177]	[8, 99, 163]		[66, 70]
	Left vs. right hemisphere stroke	End-point error	Accuracy	Reach	Higher error in LHD > RHD [97]; higher error in RHD > LHD [186]; LHD > RHD higher initial variable and constant error [84]; LHD undershoot target RHD overshoot target [188]; RHD > LHD higher endpoint error, both undershoot target [198]	[8–12]			
		Movement time	Efficiency	aROM; complex; grasp; reach	LHD > RHD longer movement time [84]; LHD > RHD slower movements (subcortical faster than cortical [240]; RHD > LHD longer movement time [198]; no of pauses and time is higher both hands for LHD > RHD [202]; LHD > RHD longer movement time [239]	[67, 81, 84, 97, 166, 167] [186, 187, 198, 202, 239]			[35]
		Reaction time		Complex; grasp; reach	RHD > LHD, slower reaction time [67]; LHD > RHD slower reaction time, initial component less accurate [84]	[67, 84, 166, 167, 187]			[35]
		Peak velocity	Speed	aROM; complex; reach	No difference in speed, modulation of acceleration more impaired in LHD than RHD [186]; LHD > RHD slower tapping [240]; RHD > LHD slower peak velocity and peak acceleration [198], peak velocity reduced in LHD [239]	[67, 84, 166, 167, 187, 240]			
	Lesion mask	Grip strength	Force	aROM; grasp; strength; reach	Lesion to basal ganglia, posterior insula and CST—grip force [233]	[139, 186, 233]	[83, 129]		[45, 66]
		Number of successful trials	Efficacy	Grasp; reach	VLSM showed no association of TMS effect on finger tapping frequency [226]	[53, 75, 124, 226]			
		movement time		aROM; complex; grasp; reach	VLSM showed association between cerebellar lesions and lower gains in movement time [121]; VLSM showed association superior cerebellar cortex and slow hand transport (superior and inferior cerebellar cortex associated with impaired straightness) [124]	[75, 85, 91, 96, 98, 124, 159]	[121]		[35, 45]
		Reaction time		aROM; grasp; reach	VLSM showed association between longer reaction times and lesion to orbital part of the inferior frontal gyrus (F3Orb, or BA 47, BA 45) and insula [159]	[85, 91, 159]			[35]
		Peak velocity	Speed	Grasp; reach	VLSM showed association between lesion to thalamus and PLIC and reduced motor learning as speed-accuracy [176]; VLSM showed association between superior cerebellar cortex lesions and slow hand transport [124]	[75, 98, 124, 235]			[176]
	Lesion volume	Absolute error	Accuracy	Reach	Larger lesions associated with lower accuracy bilaterally compared to unilateral and no deficit group [192]	[86, 186, 187, 192]			
		movement time	Efficiency	aROM; complex; grasp; reach	Lesion volume and movement time or peak velocity unrelated [86]	[85, 86, 96, 98, 146, 159, 186, 187, 192, 198]			
		Variable error	Precision	Reach	Lesion volume larger in patients with apraxia [85]; lesion volume and variable error unrelated [86]	[85, 86, 186]			
S DWI	Fractional anisotropy (FA)	Grip strength	Force	aROM; grasp; strength	Mean FA M1—grip r = 0.87, mean FA PMd—grip r = 0.72 [191]; CL anterior corona radiata—grip IL and CP-CST—grip [89]; FA—grip r = 0.94 [139]; dw-weighted CST injury—6 month grip r = 0.94 [99]; grip—CST(FA) r = n.s. [177]; T1 CST ∆AD—subacute grip r = 0.88, T1 CST ∆AD—T3 grip r = 0.85, T2 CST ∆FA—T3 grip r = 0.70 [83]; increased local WM efficiency with training [193]	[50, 89, 108, 114, 139, 155, 168, 177, 190]	[83, 99]		[66, 70, 193]
				aROM; grasp; strength	Initial grip force ratio no significant predictor for recovery that is related to FA (and MEP ±) [200]; FA ratio (and rMT) explained deficits (grip ratio) [114]	[114, 191]	[179, 200]		
F fMRI	BOLD contrast	Grip strength	Force	aROM; complex; strength; reach	CL IPL—grip recovery r = 0.65 for 6-month and 0.63 for 24-month [88]; strength and accuracy index—shift from IL to CL M1 [194]	[95, 155, 229, 231]	[88, 171, 172, 195, 200, 216]	[36, 196]	[194, 207, 228]
		Number of successful trials	Efficacy	aROM; grasp; reach	rTMS on CL M1 associated with no of finger taps and CL PMd and IL preSMA BOLD contrast [157]; IL M1 BOLD signal—number of fist closures r = 0.74 [171], ∆ tapping—rTMS in subcortical [36]; IL PMv-SMA and IL SMA-M1—improved finger tapping frequency [228]	[76]	[171, 222]		[157, 225, 228]
		Movement time	Efficiency	aROM; grasp	Reaction time—IL SMA, IL DLPFC, IL ACC and bilateral precuneus (regression) [197]; IL cerebellum—movement time r = 0.71 [51]; movement time improved after treatment and related to brain activation ratio [218]	[182]	[51]		[157, 197, 218]
		Normalized jerk	Smoothness	aROM; complex; grasp	IL premotor—normalized jerk r = 0.77, IL insula—normalized jerk r = 0.778, IL cerebellum—normalized jerk r = 0.83 [51]; normalized jerk improved after 3 weeks treatment and related with return from bilateral to unilateral and IL activation during fMRI [218]; normalized jerk during fMRI n.s. different between sessions [134]		[51]		[134, 218]
		Movement amplitude	Spatial posture	aROM; complex; reach	aIPS interference improves finger tapping amplitude [95], smaller amplitude and additional activation CL middle temporal gyrus in stroke [222]	[95]	[222]		[197, 228]
F EEG	EEG-EEG coherence	Grip strength	Force	aROM; strength	IL M1 interhemispheric beta coherence—grip r = -0.61 [160]	[201]	[58]	[160]	[156]
	Functional connectivity (FC)			Strength	CL PMd- ILM1—recovery [133]; IL M1 interhemispheric IPC—grip [160]	[131, 160, 221]	[133]		[156]
	Power analysis			aROM; complex; grasp; strength	aIPS-M1 higher alpha band frequency—grip, regression coefficient = − 0.33, aIPS-M1 beta band frequency– grip regression coefficient = − 0.43 [46]	[46, 162, 168, 238]			[52, 55]
	ERD/ERS			aROM	Event-related desynchronization (ERD)/event-related synchronization (ERS) increased in alpha band frequency (8–13 Hz) central, temporal and parietal, beta band frequency (14–32 Hz) parietal and frontal and grip strength gains with BCI [55]	[162]	[58]		[55]
	Movement-related cortical potential (MRCP)			aROM; grasp; strength	Grip strength and MRCP and MEP amplitude increase with rTMS n.s. [149]; 12 sessions BCI associated with MRCP modulation and motor improvement [43]	[43, 110]		[149]	
F TMS	transient inhibition	Movement time	Efficiency	Grasp; reach	iPMd TMS—finger movements r = 0.62 (PMd TMS slows movements) [248]; interference of cl aIPS detoriated grasp accuracy, interfering with cl M1 impaired grasping speed [94]; interference with aIPS improve finger tapping [95]	[91, 94]			
	Motor evoked potential (MEP) amplitude			Complex; grasp; reach	MEP max with lesion volume—movement time regression coefficient = − 0.46 [50]; increased MEP amplitude and improved accuracy not movement time with rTMS [118]	[50]	[112]	[126]	[92, 116, 118, 203]
		Grip strength	Force	aROM; complex; grasp; strength	MEP amplitude—grip r = 0.49, MEP threshold—grip r = − 0.54 [213]; MEP amplitude—grip r = 0.52 [212]	[50, 175, 212]	[129, 200]		[32, 69, 116, 118, 178, 225, 232, 246]
	Silent period (SP)			Grasp; strength	cM1-SP durration—grip r = 0.6, cM1-SP depth—grip r = 0.52 [183]	[136, 183]		[160]	[184]
	TMS map area			aROM; grasp; strength	TMA area—grip r = 0.52, shift of TMS area—grip r = 0.79 [212]	[212]	[129]		[184]
	Resting motor threshold (rMT)	Peak velocity	Speed	aROM; grasp	rMT—wrist peak acceleration different in stroke when compared to healthy controls [50]	[50, 95]	[217]	[82]	[102, 157, 180]

aIPS, anterior intraparietal sulcus; ACC, anterior cingulate cortex; AD, axial diffusivity; aROM, active range of motion; BOLD, blood-oxygenation-level–dependent; CL, contralesional; CST, corticospinal tract; CP, cerebral peduncle; DLPFC, dorsolateral prefrontal cortex; F, functional brain measurements; FA, fractional anisotropy; I-C, interventional cross-sectional study; I-L, interventional longitudinal study; IL, ipsilesional; IPL, inferior parietal lobule; IPS, intraparietal sulcus; LHD, left hemisphere damage; MEP, motor evoked potential; M1, primary motor cortex; O-I, observational cross-sectional study; O-L, observational longitudinal study; PLIC, posterior limb of the internal capsule; PMd, dorsal premotor cortex; RHD, right hemisphere damage; rMT, resting motor threshold; rTMS, repetitive transcranial magnet stimulation; S, structural brain measurements; SMA, supplementary motor area; VLSM, voxel-lesion symptom mapping; WM, white matter

Structural brain-related measures, including CST injury, hemispheric side of stroke, lesion masks, lesion volume, and FA, mainly of the CST, were assessed in relation to upper-limb movement measures such as grip strength, accuracy, movement time, reaction time, and peak velocity. Greater CST injury, quantified as lesion load on a CST template, was significantly associated with reduced recovery of grip strength in O-L studies. [8, 99, 163] Similarly, higher FA measures and greater increases in FA within the CST and related motor regions (e.g., M1, dorsal premotor cortex [PMd], corona radiata, and cerebral peduncles) were significantly associated with improved grip strength in O-C studies [89, 139, 190] and greater grip strength recovery in O-L studies. [83, 99] Several O-C studies reported differences in movement accuracy, efficiency, and speed between left- and right-hemisphere strokes. Compared with healthy controls, longer movement times and reduced speed were consistently demonstrated following left-hemisphere stroke (LHD) [84, 186, 202, 239, 240], whereas higher endpoint errors were repeatedly observed after right-hemisphere stroke (RHD) [146, 187, 198, 239]. Additionally, lesion masks were used in VLSM, e.g., identifying lesions in cerebellum linked to longer movement times [121, 124]; lesions in the orbital part of the inferior frontal gyrus and the insula, linked to slower reaction times [159]; and lesions in the thalamus and posterior limb of the internal capsule, linked to impaired motor learning as reflected by reduced improvements in speed and accuracy over time in an I-L study [176]. In addition,, associations with larger lesion volumes were associated with reduced reaching accuracy in both arms [192], although no significant associations were observed between lesion volume and movement time or variable error in O-C studies. [86].

Functional brain-related measures, including fMRI-based BOLD contrast; EEG-derived measures such as EEG–EEG coherence, power analysis, event-related desynchronization (ERD), and MRCPs; and TMS-derived measures including MEP amplitude, SP, TMS map area, rMT, and transient inhibition were assessed in relation to grip strength, number of successful movement trials, movement time, and normalized jerk as a measure of movement smoothness. Increased BOLD signals in IL M1 [171], as well as enhanced connectivity between IL ventral premotor cortex (PMv)-supplementary motor are (SMA) and IL SMA-M1 [228], were significantly associated with the number of successful movements in longitudinal study designs. Additionally, increased BOLD signals in the IL cerebellum were correlated with improved movement times, and increased BOLD signals in IL premotor cortex, insula, and cerebellum were associated with improved normalized jerk during stroke recovery [51]. Regarding EEG measurements, cross-sectional studies reported that increased IL M1 beta EEG–EEG coherence and stronger functional connectivity between IL M1 and the intraparietal cortex(IPC) [160], as well as higher alpha- and beta-band activity within the anterior inferior parietal sulcus (aIPS)–M1 network [46], were associated with greater grip strength. Longitudinal studies further showed that increased functional connectivity between CL PMd and IL M1 [133], and increased event-related desynchronization (ERDs) in the alpha and beta frequency band over central, parietal and frontal regions [55], were related to recovery in grip strength over time. With respect to TMS measurements, O-C studies demonstrated that higher MEP amplitudes were significantly correlated with greater grip strength [212, 213] and shorter movement times [50]. A higher resting motor threshold was reported in stroke subjects compared to healthy controls and associated with lower peak velocity [50]. Moreover, transient inhibition of IL PMd [248] and IL M1 [94] resulted in slower movements, whereas interference of CL aIPS led to reduced grasping accuracy [94], but improved finger tapping [95].

#### Differences between subjects after stroke and healthy controls

The following section summarizes research comparing brain-related and UL movement-related measures between subjects with stroke and healthy controls, including differences in their expression and in the strength or nature of reported associations. Such comparisons are essential for establishing the discriminative validity, sensitivity and specificity of these measures. Among the 122 identified studies that included comparisons between subjects after stroke and healthy controls, 87 studies employed an O–C design, 18 an O–L design, 5 an I–C design, and 12 an I–L design.

Studies employing structural brain-related measures, such as lesion masks or information on the hemispheric side of stroke, reported slower movement execution [84, 217] and reduced movement accuracy in subjects after stroke compared with healthy controls [97, 112, 130, 159, 248, 250]. Some studies reported that UL movement characteristics in subjects with LHD were comparable to those of healthy controls but not in those with RHD [135, 198], whereas other studies reported the opposite pattern, with subjects with RHD showing movement characteristics closer to healthy controls than those with LHD [84, 97, 188]. These inconsistencies may partly reflect differences in the task employed, including reaching tasks, bimanual tasks, or wrist pronation-supination movements, as well as potential differences in severity levels of post-stroke motor deficits.

Research using functional brain-related measures, particularly fMRI, reported additional recruitment and increased activation of CL M1 in subjects after stroke compared with healthy controls [94, 95, 142, 229]. Furthermore, patients with mild deficits and good recovery showed brain activation patterns similar to healthy controls, whereas more severely affected and less well recovered stroke subjects exhibited globally reduced activation [78, 94, 171, 249, 250]. EEG studies reported reduced CMC in stroke subjects when compared to healthy controls [72, 132, 214, 244]. However, findings regarding the direction of the CMC changes in relation to recovery were not entirely consistent. While several studies reported that increases in CMC values towards levels observed in healthy controls were associated with improved UL movement recovery (e.g., improved reaching performance, and better grip force control, [72, 132]) another study observed higher CMC in stroke compared to healthy controls and reported that decreases or normalization of CMC values were associated with improved range of motion [68]. These findings suggest that changes in CMC, particularly shifts towards normalized CMC patterns, are related to motor recovery, although direction of change might depend on baseline levels and task context. Using TMS, CST-related MEP amplitudes were reported to be smaller in subjects after stroke than in healthy controls [50, 142, 165].

In summary, most identified studies comparing subjects after stroke with healthy controls focused on group differences in either brain-related or UL movement-related metrics, whereas direct comparisons of brain-UL relationships across groups were only rarely performed (e.g., more focal brain activation associated with larger ranges of motion in healthy individuals versus more widespread and less efficient activation patterns associated with reduced ranges of motion in individuals after stroke).

#### Task-dependent differences in brain-related and UL movement-related measures

This section summarizes how the use of brain-related measures and UL movement-related measures may vary as a function of the type of UL movement task assessed (e.g., single-joint isometric strength test versus a multi-joint grasp task or tasks with increased motor or cognitive complexity), herein defined subgroups of *strength* task, *aROM* task, *complex* task, *reach* task, and *grasp* tasks.

Brain and UL measurement system and metric usage were summarized and visualized according to the UL movement task group by considering tasks related to ICF body functions and structures or ICF activities (SM Fig. 2A–SM Fig. 6C). Of the 232 included studies, 75 focused on strength, 65 on aROM, 38 on reaching, 27 on grasping, and 27 on complex tasks. Amongst studies with tasks related to ICF *body functions and structures*, 13 used combinations of *aROM* and *strength* [67, 88, 102, 105, 170–172, 178, 195, 200, 225, 226, 252]. In the ICF *activity*-related task domain of *reach*, *grasp*, and *complex* tasks, 9 studies compared different tasks within one or more population [35, 51, 94, 95, 114, 157, 228, 253, 254]. Brain-related and UL movement-related measures were assessed simultaneously in 50.3% of experiments examining *strength*, 69.0% of experiments examining *aROM,* 58.2% of experiments involving *complex* tasks, 42.9% of *reach* tasks, and 47.0% of *grasp* tasks. The most frequently reported UL categories differed across task groups: force measures were most common in *strength* tasks, efficiency measures in reach task, and accuracy measures in *complex* tasks (see SM Figs. 2-6C). Similarly, the type of brain-related measures varied between tasks, with MRI-based measures being most frequently used in relation to *strength* and *reach* tasks, TMS primarily applied in *aROM* and *grasp* tasks, and fMRI most commonly employed in *complex* tasks. The most frequently reported brain-related metrics included BOLD contrasts in *strength* (12.2%), *aROM* (17.5%), and *complex* tasks (19.0%), hemispheric side of stroke in reach (11.8%), and rMT (14.5%) in *grasp* tasks (see SM Figs. 2-6B). As illustrated in SM Figs. 2-6A, the brain regions of interest similarly varied according to the task type. For *reach* tasks, studies most frequently examined the lesioned hemispheric side and M1 as well as activation of secondary motor areas. For *strength* and *aROM* tasks, investigations primarily focused on IL M1 and CL M1. In contrast, for *grasp* and *complex* tasks, studies additionally reported modulation or inhibition of secondary motor and non-motor regions, reflecting broader task-related network engagement. Further synthesis of the results was not feasible due to the heterogeneity in measurement systems, metrics and population (e.g., healthy versus stroke, severity, poor versus good recovery after stroke, and differences in post stroke phases).

## Discussion

This review synthesized current state of use of brain-related structural and functional measures and UL movement-related measures in subjects with stroke compared to healthy controls. Across 220 studies including 4954 subjects with stroke and 2709 healthy controls, a wide range of structural imaging, neurophysiological, and behavioral approaches were applied to characterize UL motor control. The overarching goal was to improve understanding of how stroke-related brain structural and/or functional pathology relates to measurable alterations in UL movement quality and quantity.

A central observation of this review is that the field remains strongly centered on CST integrity and M1-related measures, as indicated by their high frequencies compared to other motor and non-motor brain regions as shown in Fig. 2, as well as in SM Figs. 2-6A. Structural markers such as CST injury and diffusion-based metrics were most consistently associated with grip strength deficits and recovery, reinforcing their prognostic relevance. However, the predominance of CST-focused biomarkers also highlights a potential narrowing of perspective, as UL motor control depends on distributed sensorimotor [8] and higher-order networks, such as attentional [255–257] and frontoparietal networks [95, 217, 258, 259] beyond descending motor pathways.

Functional brain-related measures including fMRI-based activation patterns, EEG-derived measures such as CMC and MRCPs, and TMS-based indices of corticospinal excitability were more frequently associated with movement efficiency, smoothness, and strength-related outcomes. In particular, increased activation in IL motor regions was commonly linked to improved motor function, whereas CL recruitment was more often interpreted as compensatory or potentially maladaptive, depending on impairment severity [42, 95, 142, 143, 171, 217, 226]. Together, these findings support contemporary models proposing that recovery reflects a dynamic balance between restoration of IL network function and context-dependent CL contributions.

More than 55% of the included studies included healthy control groups, allowing differentiation between stroke-related alterations and normative motor control. Nevertheless, relatively few studies systematically examined direct brain-behavior relationships across both groups, for example through parallel comparisons of brain-related and UL movement-related measures or by testing associations between neural and behavioral outcomes within and between groups. As a result, distinctions between restitution and compensation remain partly inferential. Future research should more explicitly model these mechanisms across impairment levels and recovery stages.

Task heterogeneity emerged as another key issue. Although strength tasks, finger movements, reaching, and grasping paradigms were widely used, few studies directly compared different UL tasks and difficulty levels within the same experimental design. Because neural correlates of motor control are task-dependent, the low number of within-study task comparisons [94, 260] limits interpretation and cross-study synthesis. Integrating multiple task types within unified designs in the same study participant may enable more granular phenotyping and clarify whether observed neural signatures reflect individual impairment patterns, general recovery mechanisms, or task-specific adaptations.

Methodologically, most studies demonstrated low to moderate risk of bias and reported acquisition procedures transparently. However, reporting limitations, particularly regarding assessor blinding, missing data, and participant flow remain common. Sample sizes were frequently small, likely reflecting the logistical and financial burden of multimodal neuroimaging and neurophysiological assessments, and the higher demand such studies make upon elderly stroke patients. While large-scale studies may not always be feasible, improvements in comprehensively reporting transparency and methodological rigor would enhance reproducibility and facilitate secondary analyses including data pooling.

Overall, this review underscores that meaningful characterization of post-stroke UL motor control requires integrated consideration of the neural structural reserve and dynamic functional engagement, the task demands, and population characteristics.

### Evidence on associations between brain and UL movement measures

Associations between brain-related and UL movement-related measures were heterogeneous with respect to brain modality, task characteristics, and participant profiles, precluding quantitative pooling. Nevertheless, several converging patterns emerged.

CST integrity demonstrated the most consistent relationship with grip strength impairment and recovery, reaffirming its role as a foundational biomarker. [6, 7] However, its explanatory scope appeared largely confined to force-related besides clinical outcomes (e.g., FMA-UE), suggesting that additional neural mechanisms contribute to higher-order aspects of movement control such as coordination and smoothness.

Differences in movement kinematics were reported with respect to the side of the hemispheric stroke; however, findings were inconsistent. This variability highlights the need for more systematic investigation of theoretical frameworks such as the previously presented complementary dominance hypothesis, which posits distinct contributions of the dominant hemisphere to movement execution and of the non-dominant hemisphere to impedance control [261]. The authors of the original study suggest that these lateralized functions may further influence how stroke-related lesions affect movement behavior in both arms and may inform the development of targeted treatment strategies [261].

Functional measures provided further insight into dynamic recovery processes. Activation patterns in IL motor and non-motor regions were more frequently associated with improvements in movement efficacy, efficiency, and smoothness. In contrast, CL activation was more often linked to compensatory recruitment or maladaptive plasticity, particularly in more severely impaired individuals [95, 171, 217]. These findings reinforce the notion that hemispheric contributions are not static but depend on structural damage, impairment severity, and recovery stage.

TMS studies provided important causal evidence. Modulation of IL and CL motor and non-motor regions resulted in task-specific changes in accuracy, efficiency, and speed, underscoring the functional relevance of interhemispheric interactions. Similarly, measures of CST and cortico-reticulospinal tract excitability were used to distinguish between restitution-driven improvements and compensatory strategies. Such approaches move beyond correlational evidence and contribute mechanistic insight into recovery processes.

Despite these promising findings, the joint application of brain-related and UL movement-related measures remains relatively limited and fragmented. Important modifiers, including stroke location, subtype, and chronicity, behavioral impairment severity, and the specific task characteristics especially in UL research are not yet systematically integrated into predictive models, which could help understanding the relative contribution of these covariates. Addressing these factors will be essential for refining mechanistic interpretations.

Notably, 35.91% of the identified studies investigated intervention effects using combined brain and behavioral measures. These studies highlight the potential of multimodal approaches to elucidate treatment mechanisms, differentiate neural repair from compensation, and optimize therapy selection. Taken together, the evidence supports the view that integrated brain-UL movement assessment, referenced against healthy physiological patterns and examined across diverse movement tasks, is essential for advancing precision neurorehabilitation after stroke.

### Measurement qualities and technical aspects

This review extends previous works focusing on UL kinematic assessments [25] and brain-related measures such as EEG [21] or fMRI [22, 23]. Prior reviews primarily examined the measurement properties of kinematics, EEG, or fMRI metrics and generally reported good reliability for metrics such as movement time, number of movement onsets/ends, path length ratio, peak velocity, number of velocity peaks/spectral arc length, trunk displacement, and shoulder flexion/extension [21, 25], as well as EEG-derived metrics including beta and gamma frequency bands, intra- and interhemispheric connectivity, coherence measures and phase lagged index [21]. For fMRI, endpoint tracking of the finger or hand during fMRI has been reported to have the highest reliability [22].

Herein, we also considered the temporal relationship between brain-related and UL movement-related measures, specifically whether these measures were acquired simultaneously or separately. While simultaneous acquisition can provide insights into dynamic processes and their interactions, it also poses substantial technical challenges due to the constraints of many brain-related measurement systems. For example, shoulder movements are restricted in MRI systems, and EEG recordings are susceptible to noise from large movements. This review identified 62 studies using fMRI, of which 14 studies (22.6%) did not report any simultaneously collected UL movement-related measure. This finding is consistent with previous reviews [22], which found that studies using fMRI include direct statistical analysis between brain and UL movement-related measures in only half of the studies, and did not report UL kinetics or kinematics in 14% of the studies. Studies have reported that including real-time movement information for fMRI analysis can improve the accuracy of behavioral correlations with BOLD activation [182, 222, 262], but detailed reporting of these movement signals remains uncommon. Examples are the use of EMG sensors or camera-based systems for detecting mirror movements [185], movements onsets and offsets, or providing real-time biofeedback [32, 56, 57, 194, 263, 264]. Measures of UL kinematics, kinetics, and muscle activity offer high resolution and versatility, useful for both normative and population-specific datasets, and therefore can help to improve the interpretability and analytical precision of brain-related measures. Therefore, combining experiments of brain-related and UL movement-related measures is promising to better understand neurologic control of hand and arm movements beyond observer-based ordinal outcome scales, which remain the mainstay of clinical studies. Nevertheless, acquiring and processing such data simultaneously comes with technical challenges, e.g., for hardware setup and processing, plus optimization and harmonization (e.g., movement signals are fast fluctuating whereas BOLD changes measured with fMRI are slow and stable signals) [22]. Such synchronization techniques between functional neuroimaging measures and motion capture systems are insufficiently reported in the literature. [23].

A further consideration emerging from this review concerns the integration of different brain-related measurement modalities (e.g., structural MRI, functional EEG, and TMS). While multimodal brain assessments were not the primary focus of this review, several included studies combined modalities within the same UL movement task study (e.g., fNIRS and EEG, [148] EEG and TMS, [55, 156, 160] or TMS and MRI [42, 101, 142, 195, 226, 231]), or used one modality to inform another (e.g., MRI-guided TMS targeting to increase accuracy) [247, 265].

These approaches highlight the potential of multimodal designs to improve mechanistic insight and spatial–temporal resolution.

### Recommendations for future studies

With the goal of enabling data pooling across mostly small but high-quality studies, future research should ensure transparent and standardized reporting of experimental task design (including ICF-aligned task descriptions, instructions, number of repetitions); data acquisition hardware and setup, processing steps, analysis pipelines of brain and motion data; report missing data and its handling; describe timing and blinding across assessments; and provide comprehensive participant characteristics such as demographics, time post-stroke, clinical impairment, and lesion characteristics.

Clear distinctions and considerations of the experimental movement tasks are important. For example, grasping tasks that include reaching, hand opening, grasping and manipulation should be explicitly distinguished from grip strength tasks that primarily reflect force generation using a handheld sensor. The classification of UL movements into strength and aROM tasks (ICF body functions and structures) and reach, grasp, and complex tasks (ICF activities), as extended here from previous work, [25] may improve conceptual clarity, facilitate evidence synthesis, and better characterize distinct UL movement qualities. Incorporating complex tasks that combine both motor and cognitive components may further elucidate stroke-related real-world activity limitations, which frequently involve both domains, and improve alignment between research in healthy and clinical populations.

This review proposes five groups of UL movement task groups aligned with the ICF framework to better characterize task-specific demands on neural control and motor execution. These range from simple strength or aROM paradigms to advanced activities, such as object manipulation or dual task conditions reflecting everyday functional challenges. These recommendations are further supported by prior reviews [20, 266] demonstrating task-specific neural recruitment, including dorsal PMC involvement during grasping and engagement of secondary non-motor areas such as PFC or cingulate cortex during more complex paradigms [266]. Including multiple task types within a single study may therefore provide a better understanding of stroke-related UL impairments within the inherent complexity of human UL functioning.

Finally, comprehensive reporting and open-source access to the individual-level datasets via online repositories are critical to enable cross-study integration. Such efforts will allow quantitative evaluation of post-stroke motor control reorganization, lesion-behavior relationships, longitudinal recovery trajectories, and individual recovery potential. Establishing shared standards for data pooling is essential to advance mechanistic insight and support the development of precision neurorehabilitation strategies.

### Limitations and strengths of the systematic review

Several strengths and limitations warrant consideration. By capturing the full spectrum of brain and UL measurement systems, metrics, analytical approaches, and task paradigms across stroke and healthy populations, we identified a large number of eligible studies; however; this breath necessarily increased methodological heterogeneity. Consequently, the level of detail in directly comparing studies, particularly regarding the quantification of the brain-UL relationship and contrasts between stroke and healthy populations, was limited.

Given our objective to evaluate multiple key factors in parallel, we deliberately prioritized comprehensiveness over narrow methodological focus. Finally, although classifications of experimental task groups, measurement systems, and metrics were grounded in established literature, some degree of conceptual overlap between categories cannot be excluded.

## Conclusions

This review identified a rich but also heterogeneous growing body of research that reflects technological advances in both brain-related and UL movement-related measurement systems, devices, and related metrics. The identified studies had an overall low to moderate risk of bias, and across studies indicated several common associations between brain-related and UL movement characteristics. As details were incomplete in many studies, the current findings emphasize the need for future studies to include both detailed brain-related and UL movement-related measurements, as possible, as well as comprehensive reporting on experimental task design, brain and UL measures, and subject characteristics including lesion anatomy. Doing so will facilitate meta-analyses and advance the therapeutic utility of these measures.

## Supplementary Information

Additional file1 (PDF 2933 KB)

## Data Availability

The data used for this study will be shared by the authors upon request, without undue reservation.
