# Supplementary material for: Systematic review on the association between brain-related measures and upper limb movement characteristics after stroke
Source: J Neuroeng Rehabil. 2026 Aug 8;23:315. doi: 10.1186/s12984-026-02094-4 (PMC13732956; doi:10.1186/s12984-026-02094-4)
Supplement: Supplementary file 1 [file 12984_2026_2094_MOESM1_ESM.pdf]

SUPPLEMENTAL MATERIAL

Systematic review on the association between brain-related measures and upper limb movement characteristics after stroke

Anne Schwarz[1,2], Min-Keun Song[1,2,3], Ming Wu[4], Rachel Wing[1,2], Shivani Sakthi[1,2], Maeve Settle[1,2] , Eden Farahmand[1,2], Matthew Hansen[1,2], Janne Marieke Veerbeek[5,6,7], Steven C. Cramer[1,2].

[1] Department of Neurology, David Geffen School of Medicine at UCLA, Los Angeles, California, United States.
[2] California Rehabilitation Institute, Los Angeles, California, United States.
[3] Department of Physical & Rehabilitation Medicine, Chonnam National University Medical School, Gwangju, Republic of Korea.
[4] Department of Medicine, Rutgers Robert Wood Johnson Medical School, New Brunswick, New Jersey, United States.
[5] Clinic for Neurology and Neurorehabilitation, Lucerne Cantonal Hospital, University Teaching and Research Hospital of the University of Lucerne, Lucerne, Switzerland.
[6] School of Exercise Science, Physical & Health Education, University of Victoria, Victoria, BC, Canada.
[7] Institute on Aging & Lifelong Health, University of Victoria, Victoria, BC, Canada.

Corresponding author:
Anne Schwarz
UCLA
Department of Neurology
710 Westwood Plaza, Reed C239
Los Angeles, CA 90095-1769
United States
EMAIL: anneschwarz@mednet.ucla.edu

Table of content

1. MATERIAL AND METHODS 3
1a. Definitions 3
1b. Eligibility criteria 3
1c. Information sources and search 3
1d. Study selection 4
1e. Data collection 4
1f. Risk of bias assessment 5
1g. Synthesis of results 5
2. RESULTS 5
3. REFERENCES 61

1. MATERIAL AND METHODS

1a. Definitions

Stroke was defined as "a central nervous system infarction attributable to ischemia or hemorrhage based on neuropathological, neuroimaging, and/or clinical evidence of permanent injury that incorporates specific clinical and tissue criteria".(1) The time poststroke was defined as hyper-acute (0 to 24 hours), acute (1 to 7 days), early subacute (7 days to 3 months), late subacute (3 to 6 months), and chronic (> 6 months).(2)

Brain measures (e.g., right vs. left/ ipsilesioned vs. contralesional hemisphere, ROI) - We define brain measures as any quantity describing direct or indirect measures of brain structures and functions. Brain structures can include general volumetric brain measures, regions of interest in the brain as well as location and size of stroke lesions. Brain function and neural activity might be measured by means of electrical activity (e.g., electroencephalogram [EEG]), changes in blood flow and regional oxygenation (e.g., functional magnet resonance imaging [fMRI], functional near-infrared spectroscopy [fNIRS]), or applications of regional magnetic fields (e.g., transcranial magnetic stimulation [TMS]). Side of stroke was considered a brain measure only when analyzed in relation to a study research question.

UL kinematic, kinetic and muscle activity measures (e.g., key pressing, joystick) - We define upper limb (UL) kinematics and kinetics as physical movement properties that act on the upper extremity segments and joints from the shoulder girdle until the fingertips, whereas kinematics include motion characteristics and kinetics include properties of force and torque. Measures of muscle activity include electromyography (EMG) that captures myoelectric signals or ultrasound imaging for assessing muscle contraction and changes in muscle size and shape UL measures might include time-based measures and the number of successful task completions, but no ordinal classification schemes that rely on observer rating principles.
The identified metrics were assigned to one of the following UL movement categories that are defined as follows. *EMG* entails measures of electrical activity in muscles with surface electrodes. *Force* describes measures of strength, power, energy, or torque produced (e.g., grip strength, pinch force). *Accuracy* describes the spatial error of movements relative to optimal behavior (e.g., relative to a straight line). *Data driven scores* apply statistical transformations on either sensor data or metrics to construct an abstract score that is not intuitively relatable to physiological behavior (e.g., principal component analysis). *Efficacy* describes whether a targeted task goal could be successfully achieved or not. *Efficiency* defines the quality of how a targeted task goal was reached. *Movement planning* captures the ability to perform goal-directed tasks in a feedforward manner. *Precision* is the variability of performance across multiple repetitions of the same task. Smoothness analyzes the quality of feedforward control based on the deviation of the velocity profile to an optimal, bell-shaped velocity profile. *Spatial posture* describes position-related aspects of single or multiple joints. *Speed* refers to how fast movements were performed. *Temporal posture* refers to dynamic posture state changes that often focus on patterns or rhythm and include factors like sway frequency or variability.(3)

1b. Eligibility criteria

This review included primary studies on persons after stroke of 18 years or older, with or without a healthy control group, that investigated measures of brain activation and arm movements. There were no restrictions in terms of timing poststroke or stroke severity. Cross-sectional and longitudinal observational and intervational studies (e.g., cohort studies, randomized controlled trials) with ≥10 participants that included measures of brain activity and connectivity (e.g., primary motor cortex activation, CST injury) and kinematic and/or kinetic measures (e.g., movement speed, hand trajectories, joint angles, smoothness measures, instrumented muscle strength, EMG) were considered in this review. Publications in peer-reviewed journals written in English, were considered for inclusion.

Identified hits were excluded if other neurologic diseases (e.g., cerebral palsy / Infantile Cerebral Palsy, Parkinson disease, spinal cord injury, epilepsy, Alzheimer's, psychiatric disorders such as schizophrenia or depression) were investigated. Neurophysiologic measures taken solely for device control accuracy are outside of the scope of this study and were excluded if not reported otherwise. Furthermore, research on motor imagery or sensory function without actual movement execution is excluded (e.g., passive movements, motor imagery, sensory assessments). Literature reviews, simulated data, or secondary data analysis were excluded, as no primary data was presented. Lastly, research on specific sport movement sciences (e.g., golf swing, swimming) was outside of the scope of this review.

1c. Information sources and search

A systematic search in the following electronic databases was performed rom inception until 30/09/2023: PubMed, EMBASE, and NeuroImage. Combinations of the keywords 'brain function' and 'kinematics/ kinetics' and 'stroke' and 'upper limb' were searched for by using MeSH terms and free text words with synonyms and related terms in title and abstract.

Search was performed in Pubmed (Box 1), Embase, (Box 2), and Neuroimage

Box 1. Pubmed search string (last searched June 09, 2026)

Search [20230609]: ((((((((((((((((((((((((brain[MeSH Terms]) OR (brain*[tiab])) OR (brain connect*[tiab])) OR (brain function*[tiab])) OR (brain activ*[tiab])) OR (functional connect*[tiab])) OR (structural connect*[tiab])) OR (cerebrum[MeSH Terms])) OR (cerebrum[tiab])) OR (cort*[tiab]))) OR (Brain/physiopathology[MeSH Terms])) OR (neuroimaging[MeSH Terms])) OR (functional neuroimaging[MeSH Terms])) OR (diffusion magnetic resonance imaging[MeSH Terms])) OR (Magnetic Resonance Imaging[MeSH Terms])) OR (diffusion tensor imaging[MeSH Terms])) OR (motor cortex[MeSH Terms])) OR (imaging, perfusion[MeSH Terms])) OR (brain mapping[MeSH Terms])) OR (magnetic resonance imag*[tiab])) OR (MRI[tiab])) OR (EEG[tiab])) OR (fNIRS[tiab])) AND ((kinematic* [tiab] OR Biomechanical Phenomena [MeSH Terms] OR velocit* [tiab] OR jerk [tiab] OR accuracy [tiab] OR reaction time [tiab] OR smooth* [tiab] OR reach* [tiab] OR point* [tiab] OR tap* [tiab] OR grasp* [tiab] OR grip* [tiab] OR Hand Strength [MeSH Terms] OR hand strength [tiab] OR Muscle Strength [MeSH Terms] OR muscle power [tiab] OR muscle activ* [tiab]))) AND ((((((((((((((((("Upper Extremity"[MeSH Terms]) OR "Upper Extremity/physiopathology"[MeSH Terms]) OR "Upper Extremity"[MeSH Terms]) OR "Upper Extremity/physiology"[MeSH Terms]) OR "Arm/physiopathology"[MeSH Terms]) AND upper extremit*[tiab]) OR upper limb*[tiab]) OR arm[tiab]) OR arms[tiab]) OR shoulder*[tiab]) OR elbow*[tiab]) OR forearm*[tiab]) OR wrist*[tiab]) OR hand*[tiab]) OR finger*[tiab])))) NOT ((Lower Extremity [MeSH Terms] OR Lower Extremity [tiab] OR Gait [MeSH Terms] OR gait* [tiab] OR walking [MeSH Terms] OR walk* [tiab] OR Postural Balance [MeSH Terms] OR balance [tiab] OR Posture [MeSH Terms] OR postur* [tiab])))) AND ((((((((("Stroke"[MeSH Terms] OR cva[tiab] OR cvas[tiab] OR poststroke*[tiab] OR stroke*[tiab] OR apoplex*[tiab])) OR ((brain*[tiab] OR cerebr*[tiab] OR cerebell*[tiab] OR intracran*[tiab] OR intracerebral*[tiab] OR vertebrobasilar*[tiab]) AND vascular*[tiab] AND (disease[tiab] OR diseases[tiab] OR accident*[tiab] OR disorder*[tiab]))) OR (cerebrovascular*[tiab] AND (disease[tiab] OR diseases[tiab] OR accident*[tiab] OR disorder*[tiab]))) OR ((brain*[tiab] OR cerebr*[tiab] OR cerebell*[tiab] OR intracran*[tiab] OR intracerebral*[tiab] OR vertebrobasilar*[tiab]) AND (haemorrhag*[tiab] OR hemorrhag*[tiab] OR ischemi*[tiab] OR ischaemi*[tiab] OR infarct*[tiab] OR haematoma*[tiab] OR hematoma*[tiab] OR bleed*[tiab]))) OR (("Hemiplegia"[MeSH Terms] OR "Paresis"[MeSH Terms] OR hemipleg*[tiab] OR hemipar*[tiab] OR paresis[tiab] OR paretic[tiab])))))) NOT ((Parkinson Disease [MeSH Terms] OR parkinson* [tiab] OR Spinal Cord Injuries [MeSH Terms] OR spinal cord injur* [tiab] OR tetrapleg* [tiab]))) Filters: Humans

Box 2. Embase search string (last searched June 09, 2023)

#5 #1 AND #2 AND #3 AND ([randomized controlled trial]/lim OR 'controlled clinical trial'/de) AND ([article]/lim OR [article in press]/lim OR [conference paper]/lim) AND ([adult]/lim OR [young adult]/lim OR [middle aged]/lim OR [aged]/lim OR [very 2elderly]/lim) AND [humans]/lim AND [clinical study]/lim
561; #4 #1 AND #2 AND #3 26,614; #3 'cerebrovascular accident' OR brain OR 'cerebellum' OR 'brain hemorrhage' OR 'brain ischemia' OR 'bleeding' OR 'paresis' OR 'hemiplegia' OR 'hemiparesis' 3,860,301; #2 ('upper extremity function' OR 'arm' OR 'elbow' OR 'hand' OR 'shoulder' OR 'forearm' OR 'finger' OR 'kinematics' OR 'movement (physiology)' OR velocit* OR jerk OR accuracy OR reaction) AND time OR smooth* OR reach* OR point* OR 'tapping' OR 'grasping' OR 'grip strength' OR 'muscle strength' OR 'muscle contraction' OR 'muscle action potential' 4,574,276; #1 (('brain connectivity'/exp OR 'brain connectivity' OR 'functional magnetic resonance imaging'/exp OR 'functional magnetic resonance imaging' OR 'nuclear magnetic resonance imaging'/exp OR 'nu-clear magnetic resonance imaging' OR 'brain'/exp OR 'brain' OR 'structural connectivity'/exp OR 'structural connectivity' OR 'motor cortex'/exp OR 'motor cortex') AND ('structural connectome'/exp OR 'structural connectome') OR 'perfusion'/exp OR perfusion) AND ('imaging'/exp OR imaging) OR 'diffusion tensor imaging'/exp OR 'diffusion tensor imaging' OR 'electroencephalogram'/exp OR 'electroencephalogram' OR 'functional near-infrared spectroscopy'/exp OR 'functional near-infrared spectroscopy

1d. Study selection

Two unblinded reviewers (A.S. and M.K.S.) screened the first 1'000 hits independently. Consensus was reached by including a third reviewer (J.M.V.) and study eligibility criteria were refined where necessary. Trained research assistants E.F., M.H., M.S., M.W., R.W., and S.S. evaluated the remainder of study hits of the search and were naïve towards the study authors. All evaluations were crosschecked by A.S. and M.K.S., and disagreements were solved by involving a third reviewer (J.M.V.) to reach consensus.

1e. Data collection

The initial data extraction sheet was designed by two reviewers A.S. and J.M.V. and piloted by A.S. and M.K.S. and iteratively adapted based on inputs from all researchers (A.S., M.K.S., M.W., R.W., S.S., M.S., E.F., M.H., and J.M.V.) and based on consensus-reaching. Data extraction forms were piloted and updated.
The form contained information regarding the first author; publication year; number of stroke participants; number of healthy participants; age; sex; time poststroke; standard clinical scale descriptions (e.g., Fugl-Meyer Assessment of the Upper Extremity, FMA-UE; Action Research Arm Test, ARAT); brain-related measurement system; brain-related

metrics; experimental motor task; UL measurement system; UL metrics; and timing of brain and UL measures.; and used metrics.
One reviewer (A.S.) extracted three-quarters of the study information and a second reviewer (M.K.S.) extracted one-quarter. Reviewers were not blinded. Three reviewers (A.S., M.K.S., J.M.V.) cross-checked comprehensiveness and correctness of the extracted data.

1f. Risk of bias assessment

The methodological quality of the included studies was assessed using an adapted version of the QUADAS-2 tool consisting of four domains: patient selection, index test, reference test, flow and timing.(4, 5) The tool and signaling questions were developed by two reviewers (A.S. and J.M.V.) and pilot tested on ten preselected studies by two reviewers (A.S. and M.K.S.). The tool consists of three signaling questions to determine risk of bias regarding the participant selection, four questions each on the information on the measures on brain activation and arm movements, three question regarding a reference measure and three signaling questions regarding the flow and timing of measures. Questions may be answered with yes, no, unclear or NA in case of healthy study participants. Six research assistants (E.F., M.H., M.S., M.W., R.W., and S.S.) that were mostly naïve towards the article authors were trained in evaluating the studies' risk of bias by using the adapted QUADAS-2 tool. Most of the risk of bias ratings (~86%) were cross-checked by two researchers (A.S. and M.K.S.).

1g. Synthesis of results

Descriptive statistics regarding patient characteristics, task characteristics, and usage of brain-related and kinematic parameters and brain regions of interest were applied. Brain regions of interest (ROIs) were grouped into whole brain, cortical motor, cortical non-motor, white matter, and subcortical ROIs, and counted for facilitation/activation or inhibition/damage, based on the potential neural modulation of the ROI, and otherwise counted neutral.
Each UL metric was categorized according to its physiological interpretation (EMG, accuracy, data driven scores, efficacy, efficiency, planning, precision, smoothness, spatial posture, speed, temporal posture, workspace) by two reviewers independently (A.S. and M.K.S.). Consensus regarding the categorization was reached through discussion. Brain-related and UL movement-related measurement systems and the related metrics were summarized in Sankey diagrams and frequency plots per task group (strength tasks, aROM tasks, reach tasks, grasp tasks, and complex tasks).

2. RESULTS

A total of 220 studies were included in the review. The summary of the risk of bias assessment across all included studies is illustrated in SM Figure 1.

2a. Risk of bias results based on the adapted version of QUADAS-2

SM Figure 1.

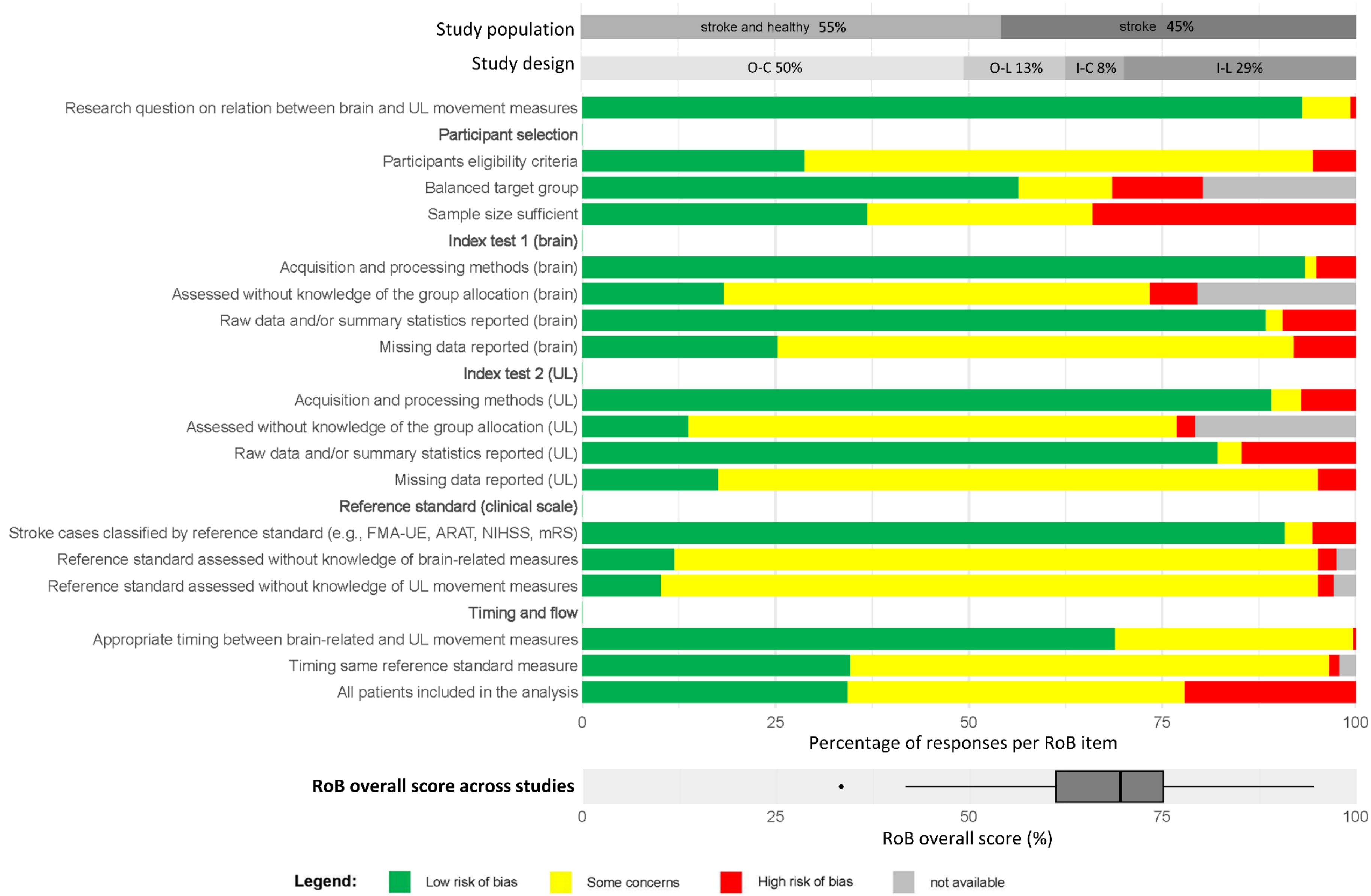

2b. Brain and UL measurements with respect to the movement task

This section provides a task-specific descriptive overview of the included studies, stratified by UL movement task (*strength*, *aROM*, *reach*, *grasp*, and *complex* tasks). For each task domain, we summarize sample characteristics, time post-stroke distribution, brain regions of interest (ROIs), neuroimaging and neurophysiological modalities, and the range of brain-related and UL-related metrics reported. This supplementary breakdown is intended to enhance transparency and support the cross-task synthesis presented in the main manuscript by detailing how brain and UL measures were implemented and combined within specific movement contexts.

Body functions and structures strength **(e.g., grip strength, pinch force, torque)**
Stroke subjects (n=1'462, mean age 58.94 years, 37% females) and healthy controls (n=699, mean age 51.1 years, 33.0% females) were included in 68 studies on strength. Stroke subjects had a median FMA-UE of 36.7 (quartile 1-3, 24-48, reported in 21 studies, 30.9%). Nine studies included stroke subjects in the acute phase, 18 studies stroke subjects in the early subacute phase, seven in the late subacute, two in subacute, and 32 in the chronic phase after stroke. The investigated brain ROI across studies including strength tasks are summarized in SM Figure 2A. Brain-related measures were assessed mostly with EEG (23% of the experiments), MRI (22.6% of the experiments), fMRI (14.2%), and TMS (13.1%) by means of a total of 57 related metrics. UL measures included mainly measures of force and muscle activity, as illustrated in SM Figure 2B and C. Grip strength, pinch force, and EMG of extensor carpi radialis (ECR), and first dorsal interosseus (FDI) muscles were most frequently reported amongst 60 identified metrics. Brain-related and UL movement-related measures were captured simultaneously in 47.1% of the experiments.

Body functions and structures active range of motion **(e.g., finger, hand, wrist, elbow, shoulder movements)**
Stroke subjects (n=1'639, mean age 60.24 years, 36% females) and healthy controls (n=1'205, mean age 58.7 years, 52.2% females) were included in 62 studies on active range of motion tasks. Stroke subjects had a median FMA-UE of 37.5 (quartile 1-3, 19.7-45.69, reported in 25 studies, 40.3%). Stroke patients were included in the acute phase in eight studies, in early subacute in 16 studies, late subacute in six studies, and in the chronic phase in 32 studies. The investigated brain ROI across studies including active range of motion (aROM) tasks are summarized in SM Figure 3A. Brain-related measures were assessed with TMS (30.7%), fMRI (22.5%), EEG (17.6%), and MRI-DWI (7.1%) by means of a total of 43 brain-related metrics. UL measures included measures of muscle activity, spatial posture, force, speed as illustrated in SM Figure 3B and C. Wrist flexion-extension angle, EMG of extensor carpi radialis (ECR) and biceps brachii (BB), grip strength, and pinch force were most frequently reported amongst 70 different identified metrics. Brain-related and UL movement-related measures were captured simultaneously in 69.5% of the experiments.

Activities of reaching **(e.g., reaching, pointing)**
Stroke subjects (n=891, mean age 60.84 years, 32.9% females) and healthy controls (n=457, mean age 60.5, 31.9% females) were included in 38 studies on reaching tasks. Stroke subjects had a median FMA-UE of 46.43 (quartile 1-3, 28.38-53.73, reported in 18 studies, 47.4%). Stroke patients were included in the acute phase in four studies, early subacute in eight studies, late subacute in one study, and in the chronic phase in 25 studies. The investigated brain ROI across studies including strength tasks are summarized in SM Figure 4A. Brain-related measures were assessed with MRI (27.4%), EEG (25.5%), TMS (24.3%), and fMRI (3%) by means of a total of 26 different brain-related metrics. UL measures included mainly measures of muscle activity, spatial posture, speed, efficiency, force and accuracy as illustrated in SM Figure 4B and C. Endpoint error, movement time, reaction time, elbow flexion-extension angle, peak velocity, and angular velocity, and EMG activity of the biceps brachii muscle were most frequently reported amongst 85 unique metrics. Brain-related and UL movement related measures were captured simultaneously in 38.8% of the experiments.

Activities of grasping **(e.g., reach-to-grasp)**
Stroke subjects (n=507, mean age 59.95 years, 36.9% females) and healthy controls (n=120, mean age 51.7 years, 31.7% females) were included in 26 studies on grasping tasks. Stroke subjects had a median FMA-UE of 41.47 (quartile 1-3 35.7 -49.67, reported in 10 studies, 38.5%) and were included in the acute phase in one study, in early subacute in five studies, late subacute in five studies, subacute phase in one study, and in the chronic phase in 14 studies. The investigated brain ROIs are summarized in SM Figure 5A. Brain-related measures were assessed mostly with TMS (26.1%), MRI (17.8%), EEG (14.8%), and fMRI (12.2%) by means of 30 different metrics as shown in SM Figure 5B and C. UL measures of correlation between peak and mean grip force, hand kinematic synergies, movement time, finger flexion-extension angle and angular velocity were most frequently reported amongst 61 the identified UL metrics. Brain-related and UL movement-related measures were collected simultaneously in 43.5 % of the experiments.

Activities of complex movements **(e.g., dual task activities, with cognitive component)**
Stroke subjects (n=455, mean age 58.87 years, 35.3% females) and healthy controls (n=228, mean age 50.4 years, 39.5% females) were included in 26 studies on complex tasks. Stroke subjects had a median FMA-UE of 40.5 (quartile 1-3, 35.5-43.3, reported in nine studies, 34.6%). The investigated brain ROIs are summarized in SM Figure 6A. Brain-related measures were assessed mostly with fMRI (34.3%), MRI (15.7%), and NIRS (14.3%) by means of a total of 23 different brain-related metrics. UL measures included mainly measures of muscle activity, efficiency, accuracy, force, speed, planning and spatial posture as illustrated in SM Figure 6 B and C. Reaction time, movement time, root mean square error, finger flexion-extension angles, and mean velocity were most frequently reported amongst 43 identified metrics. Brain-related and UL movement related measures were captured simultaneously in 62.1% of the experiments.

SM Figure 2A. Brain regions reported (including facilitation/activation or inhibition/damage) in studies on strength tasks

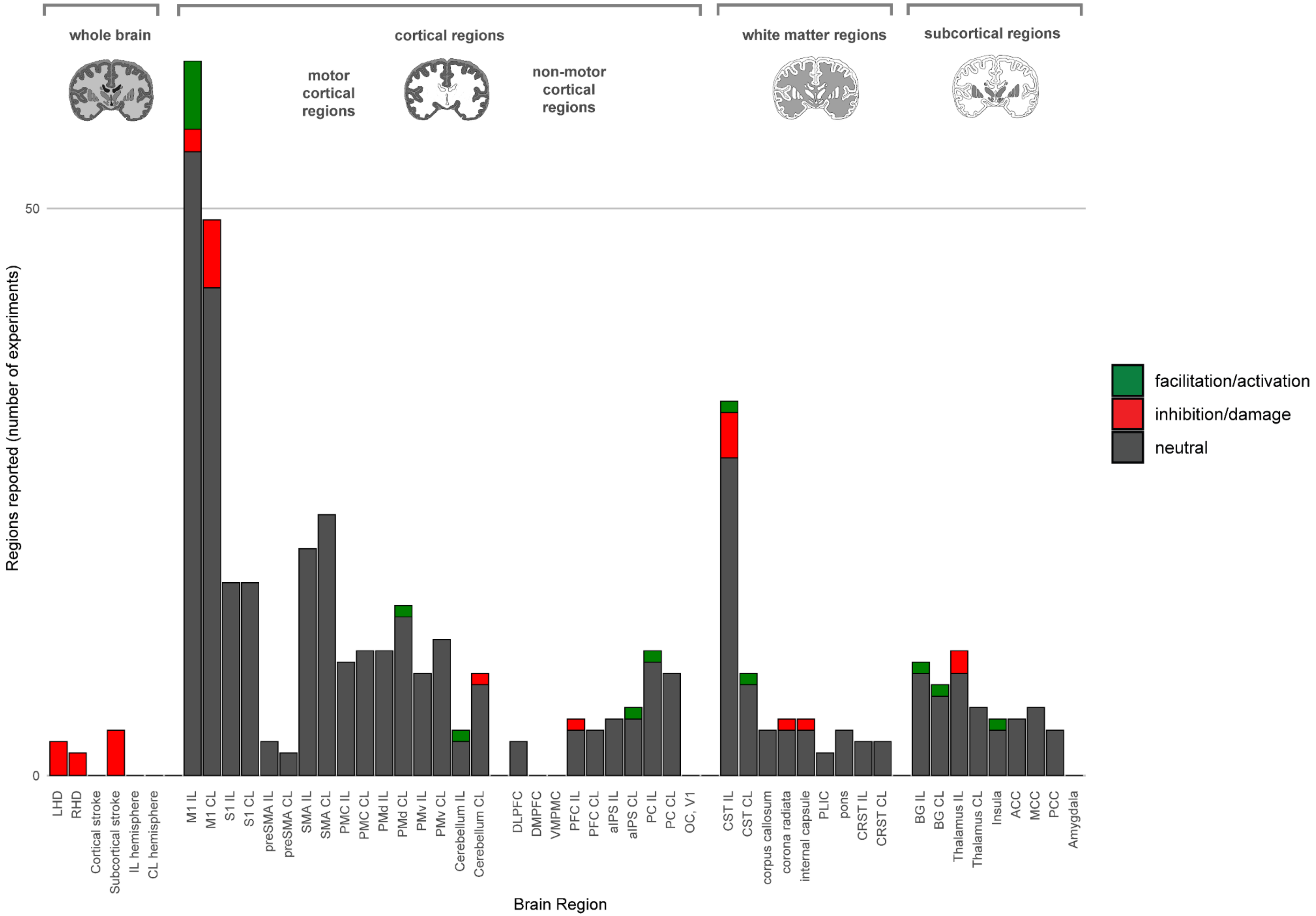

Legend: Green parts of the barplot indicate experiments, where the region of interest was stimulated or showed activations by means of brain-related metrics (e.g. motor evoked potentials, fuctional magnetic resonance imaging related contrasts), red parts indicate experiments, where the considered region of interest was damaged by the stroke lesion or showed decreased activation or inhibition, and the dark grey parts refer to experiments, where a region was mentioned in the methods but not further specified or did not show significant results and was therefore considered neutral. ACC, anterior cingulate cortex; aIPS, anterior intraparietal sulcus; BG, basal ganglia; CL, contralesional; CRST, corticoreticulospinal tract; CST, corticospinal tract; DLPFC, dorsolateral prefrontal cortex; DMPFC, dorsomedial prefrontal cortex; IL, ipsilesional; LHD, left hemispheric stroke; M1, primary motor cortex; MCC, midcingulate cortex; OC, occipital cortex; PC, parietal cortex; PCC, posterior cingulate cortex; PFC, prefrontal cortex; PLIC, posterior limb of the interal capsule, PMC, premotor cortex; PMd, dorsal premotor cortex; PMv, ventral premotor cortex; preSMA, pre-supplementary motor area; RHD, right hemispheric stroke; S1, primary sensory cortex; SMA, supplementary motor area; VMPFC, ventromedial prefrontal cortex.

SM Figure 2B. Combinations of brain-related and upper limb movement measures in studies on strength tasks

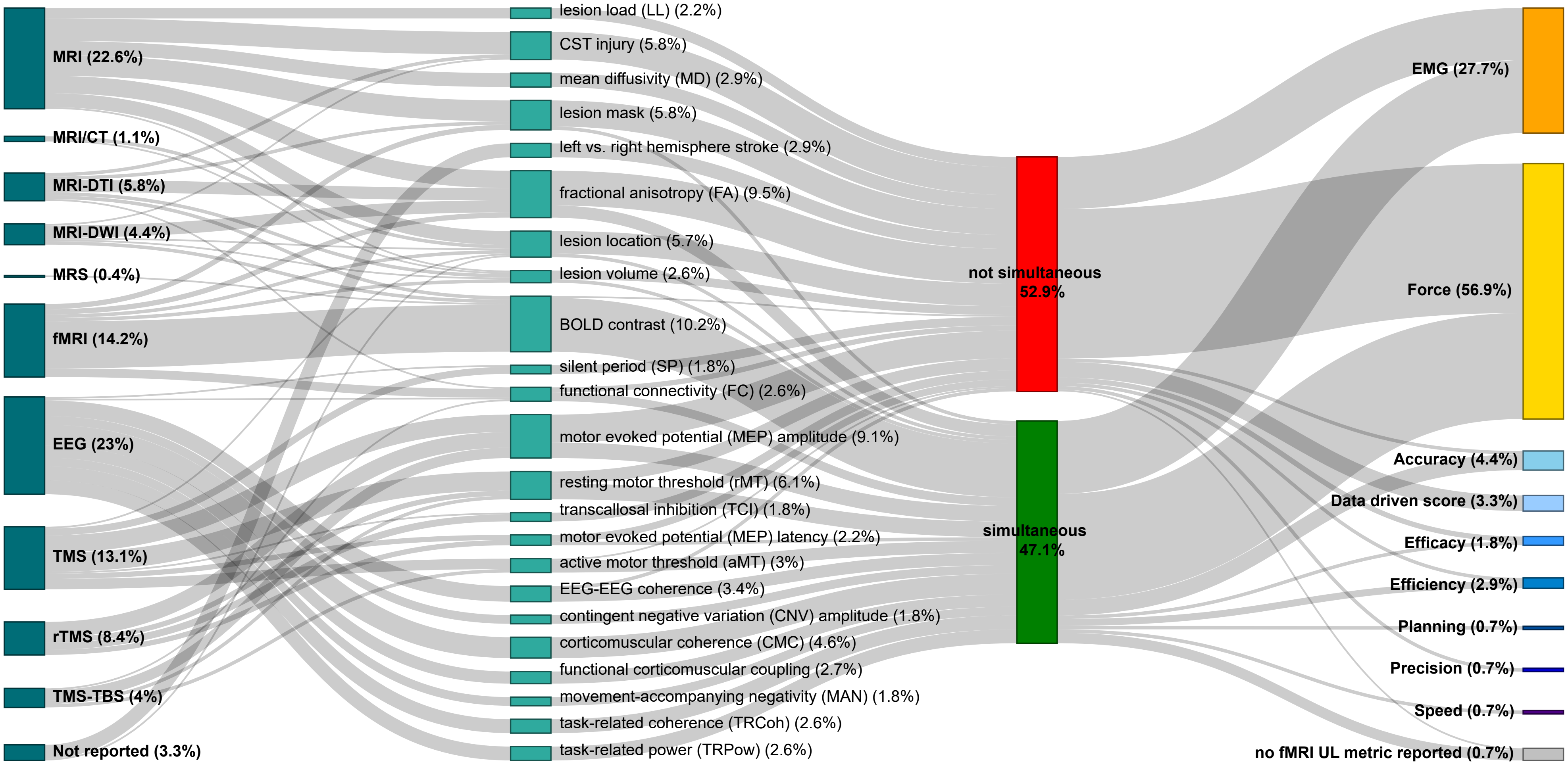

Legend: CST, corticospinal tract, DTI, diffusion tensor imaging; DWI, diffusion weighted imaging; EEG, electroencephalogram; EMG, electromyography; FES, functional electrical stimulation; fMRI, functional magetic resonance imaging; MRI, magnetic resonance imaging; MRS, magnetic resonance spectroscopy; rTMS, repetitive transcranial magnetic stimulation; SPECT, single-photon emission computed tomography; TBS, theta burst stimulation; tDCS, transcranial direct current stimulation; TMS, transcranial magnetic stimulation.

Connections represent co-occurrence of brain-related and UL movement-related measures within the same study. Links do not indicate that one measure was derived from another but reflect multimodal assessment within individual studies.

SM Figure 2C. Frequency of upper limb metrics in studies on strength tasks

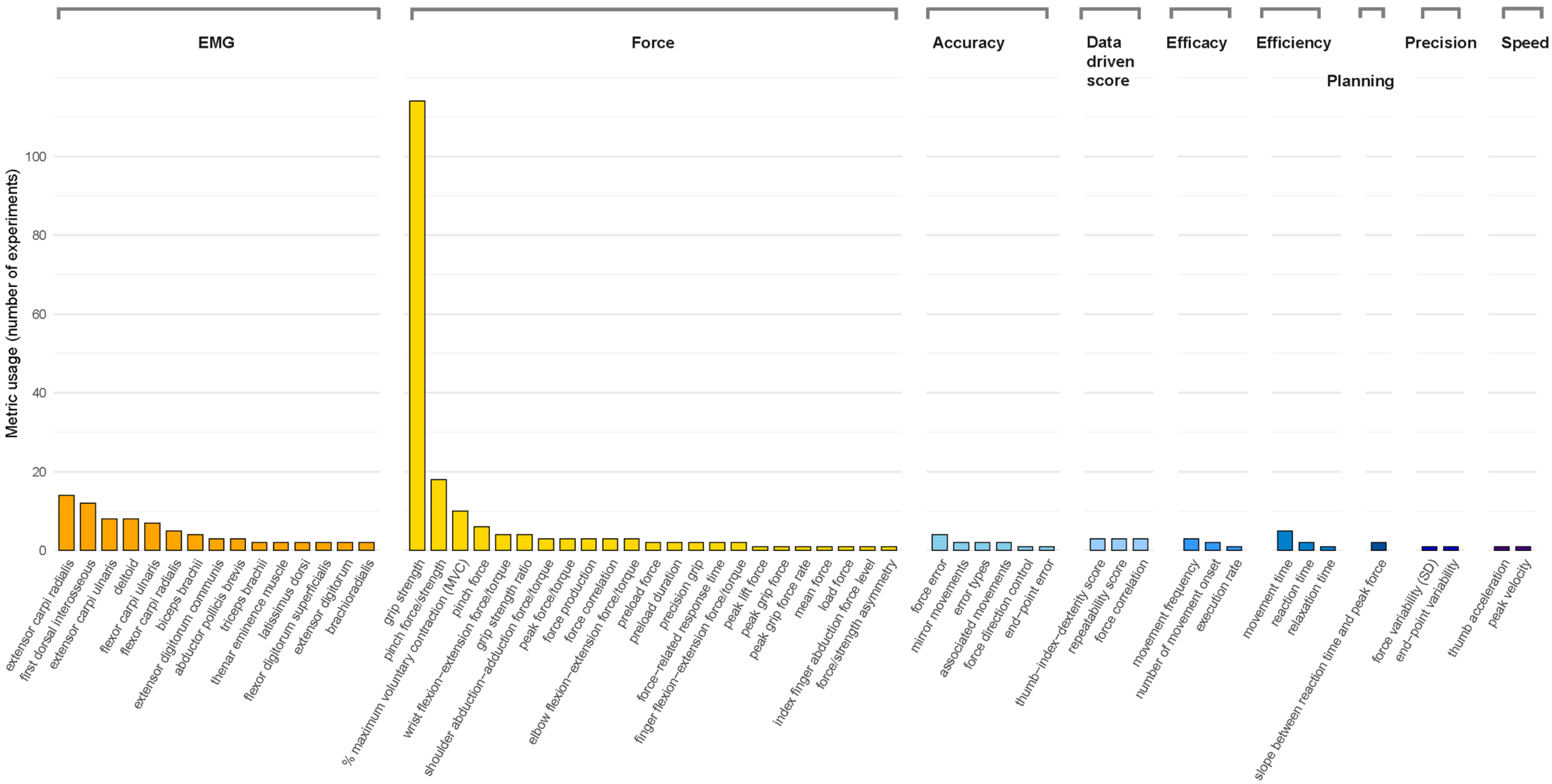

SM Figure 3A. Brain regions reported (including facilitation/activation or inhibition/damage) in studies on active range of motion (aROM) tasks

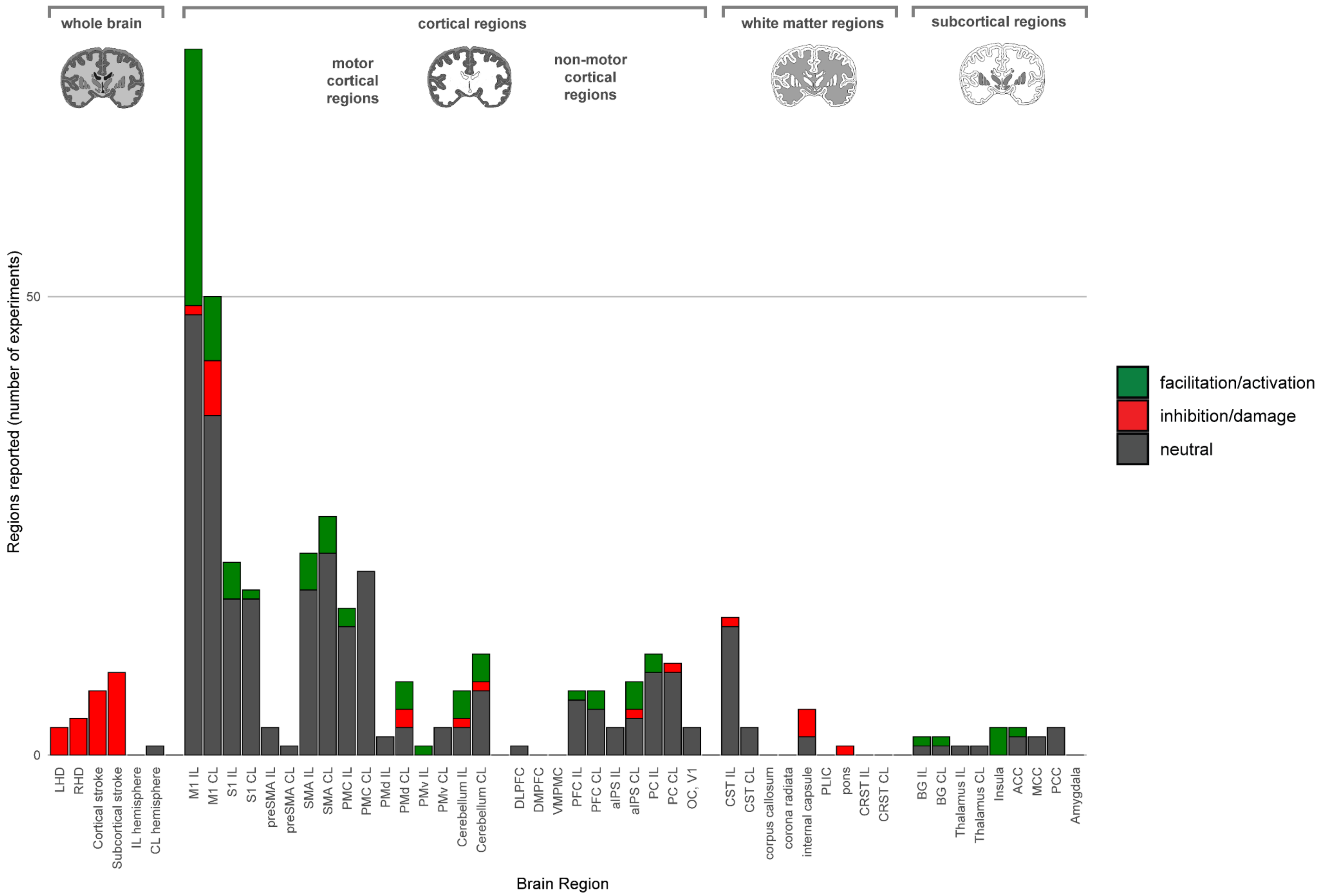

Legend: Green parts of the barplot indicate experiments, where the region of interest was stimulated or showed activations by means of brain-related metrics (e.g. motor evoked potentials, functional magnetic resonance imaging related contrasts), red parts indicate experiments, where the considered region of interest was damaged by the stroke lesion or showed decreased activation or inhibition, and the dark grey parts refer to experiments, where a region was mentioned in the methods but not further specified or did not show significant results and was therefore considered neutral. ACC, anterior cingulate cortex; aIPS, anterior intraparietal sulcus; BG, basal ganglia; CL, contralesional; CRST, corticoreticulospinal tract; CST, corticospinal tract; DLPFC, dorsolateral prefrontal cortex; DMPFC, dorsomedial prefrontal cortex; IL, ipsilesional; LHD, left hemispheric stroke; M1, primary motor cortex; MCC, midcingulate cortex; OC, occipital cortex; PC, parietal cortex; PCC, posterior cingulate cortex; PFC, prefrontal cortex; PLIC, posterior limb of the interal capsule, PMC, premotor cortex; PMd, dorsal premotor cortex; PMv, ventral premotor cortex; preSMA, pre-supplementary motor area; RHD, right hemispheric stroke; S1, primary sensory cortex; SMA, supplementary motor area; VMPFC, ventromedial prefrontal cortex.

SM Figure 3B. Combinations of brain-related and upper limb movement measures in studies on active range of motion (aROM) tasks

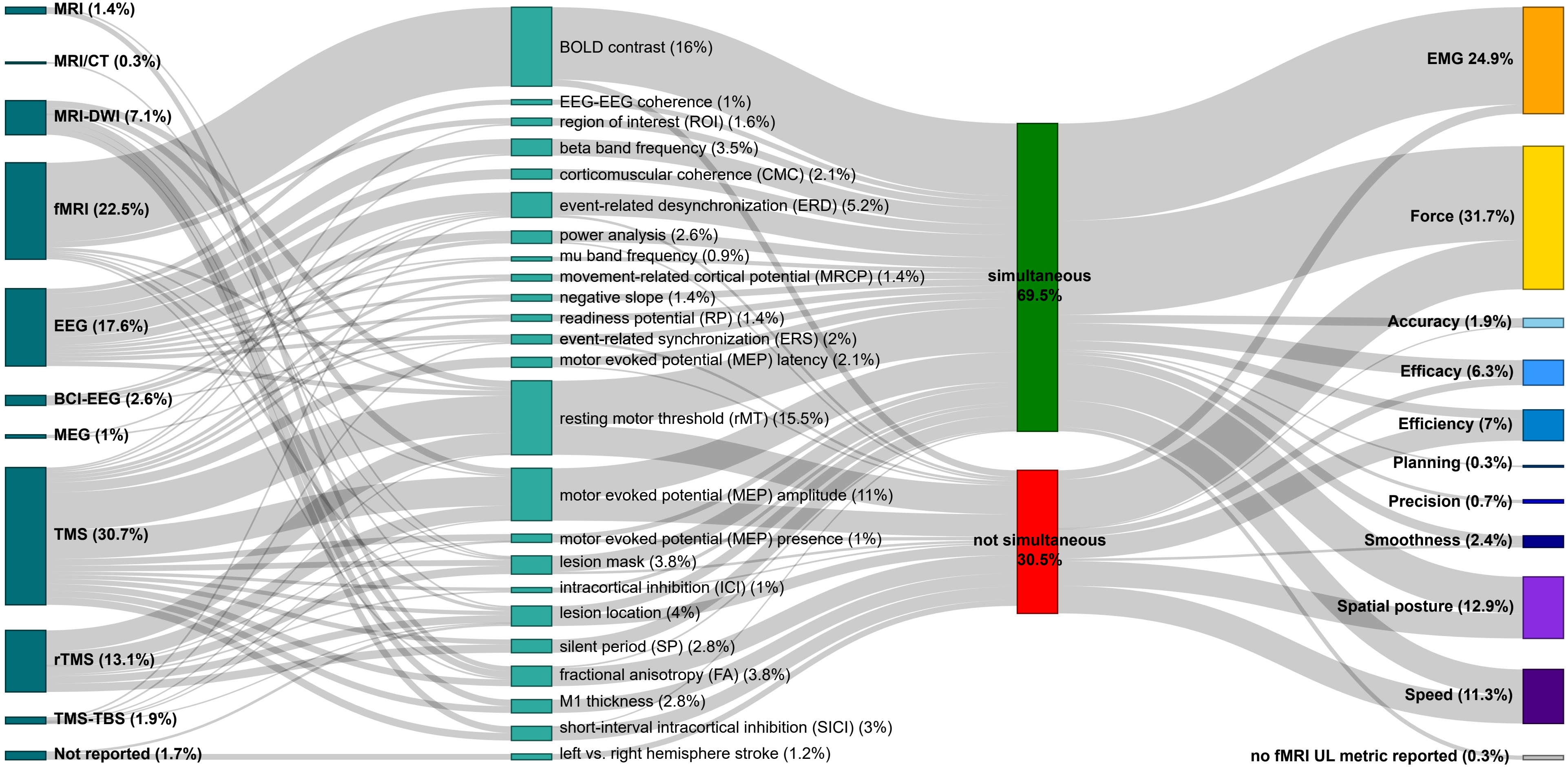

Legend: BCI, brain computer interface; CT, computer tomography, DWI, diffusion weighted imaging; EEG, electroencephalogram; EMG, electromyography; fMRI, functional magnetic resonance imaging; MEG, magnetencephalogram; MRI, magnet resonance imaging; rTMS, repetitive transcranial magnetic stimulation; tACS, transcranial alternating current stimulation; TBS, theta burst stimulation; TCD, transcranial doppler sonography; tDCS, transcranial direct current stimulation; TMS, transcranial magnetic stimulation.
Connections represent co-occurrence of brain-related and UL movement-related measures within the same study. Links do not indicate that one measure was derived from another but reflect multimodal assessment within individual studies.

SM Figure 3C. Frequency of upper limb metrics in studies on active range of motion (aROM) tasks

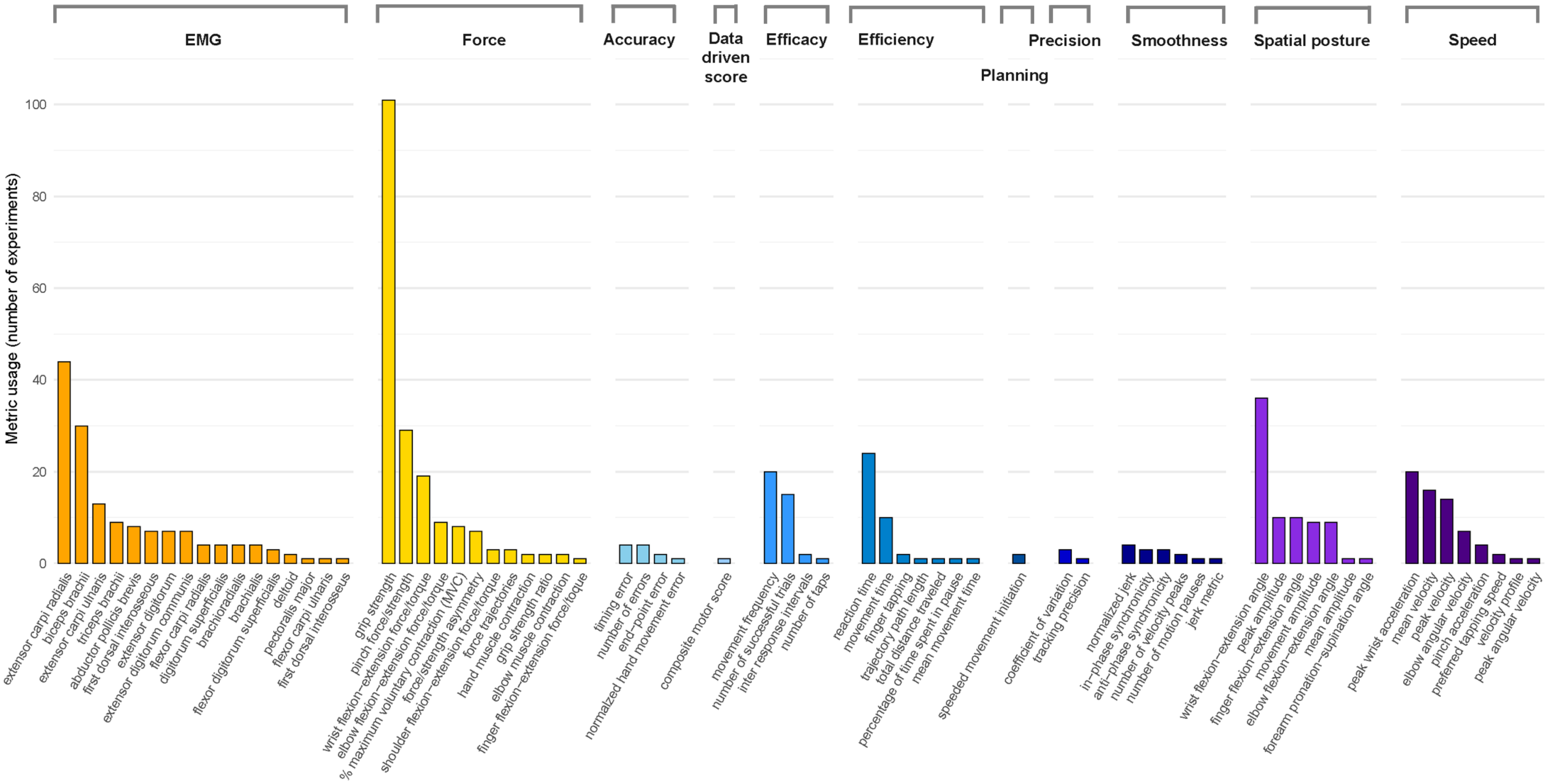

SM Figure 4A. Brain regions reported (including facilitation/activation or inhibition/damage) in studies on reach tasks

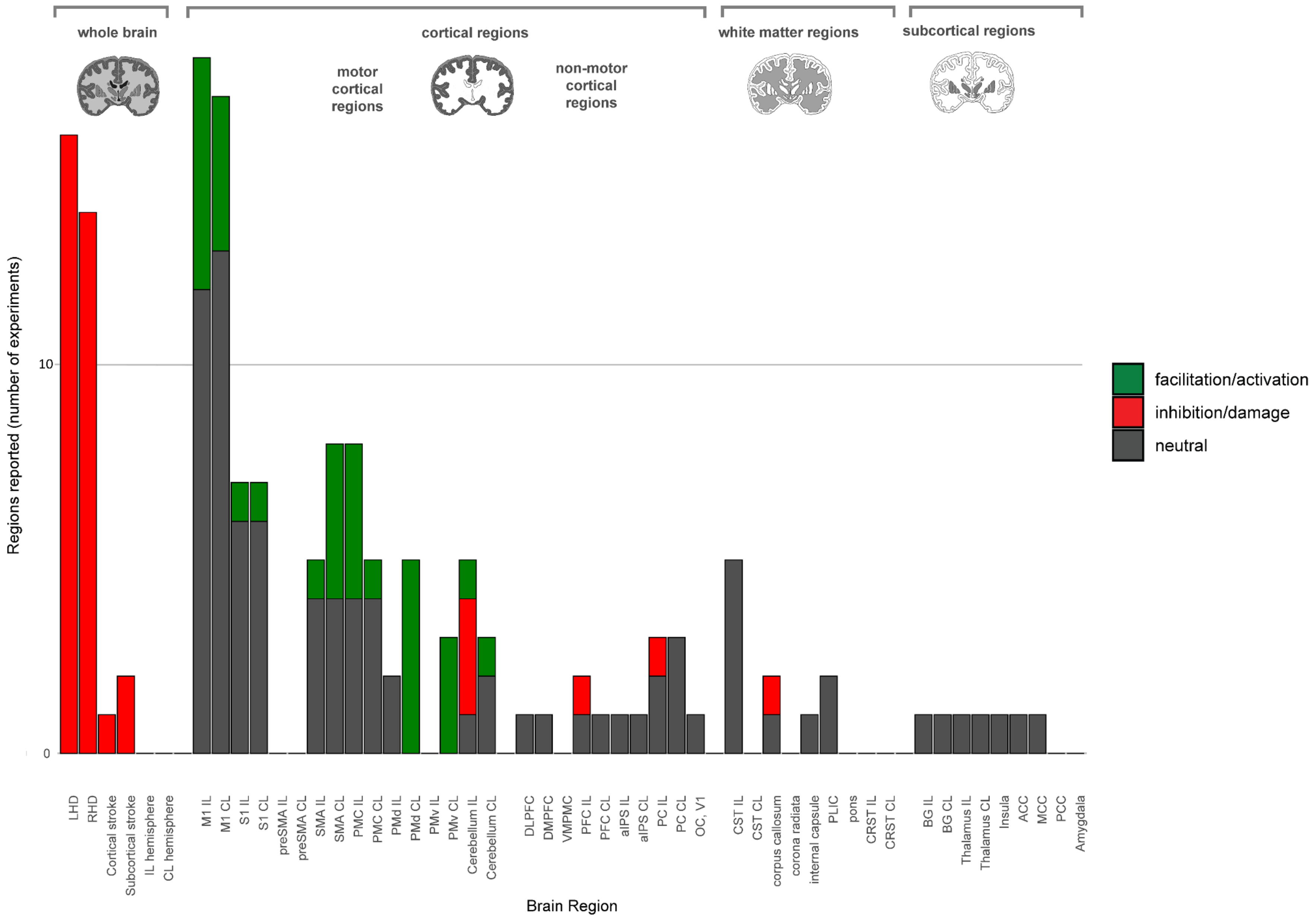

Legend: Green parts of the barplot indicate experiments, where the region of interest was stimulated or showed activations by means of brain-related metrics (e.g. motor evoked potentials, functional magnetic resonance imaging related contrasts), red parts indicate experiments, where the considered region of interest was damaged by the stroke lesion or showed decreased activation or inhibition, and the dark grey parts refer to experiments, where a region was mentioned in the methods but not further specified or did not show significant results and was therefore considered neutral. ACC, anterior cingulate cortex; aIPS, anterior intraparietal sulcus; BG, basal ganglia; CL, contralesional; CRST, corticoreticulospinal tract; CST, corticospinal tract; DLPFC, dorsolateral prefrontal cortex; DMPFC, dorsomedial prefrontal cortex; IL, ipsilesional; LHD, left hemispheric stroke; M1, primary motor cortex; MCC, midcingulate cortex; OC, occipital cortex; PC, parietal cortex; PCC, posterior cingulate cortex; PFC, prefrontal cortex; PLIC, posterior limb of the interal capsule, PMC, premotor cortex; PMd, dorsal premotor cortex; PMv, ventral premotor cortex; preSMA, pre-supplementary motor area; RHD, right hemispheric stroke; S1, primary sensory cortex; SMA, supplementary motor area; VMPFC, ventromedial prefrontal cortex.

SM Figure 4B. Combinations of brain-related and upper limb movement measures in studies on reach tasks

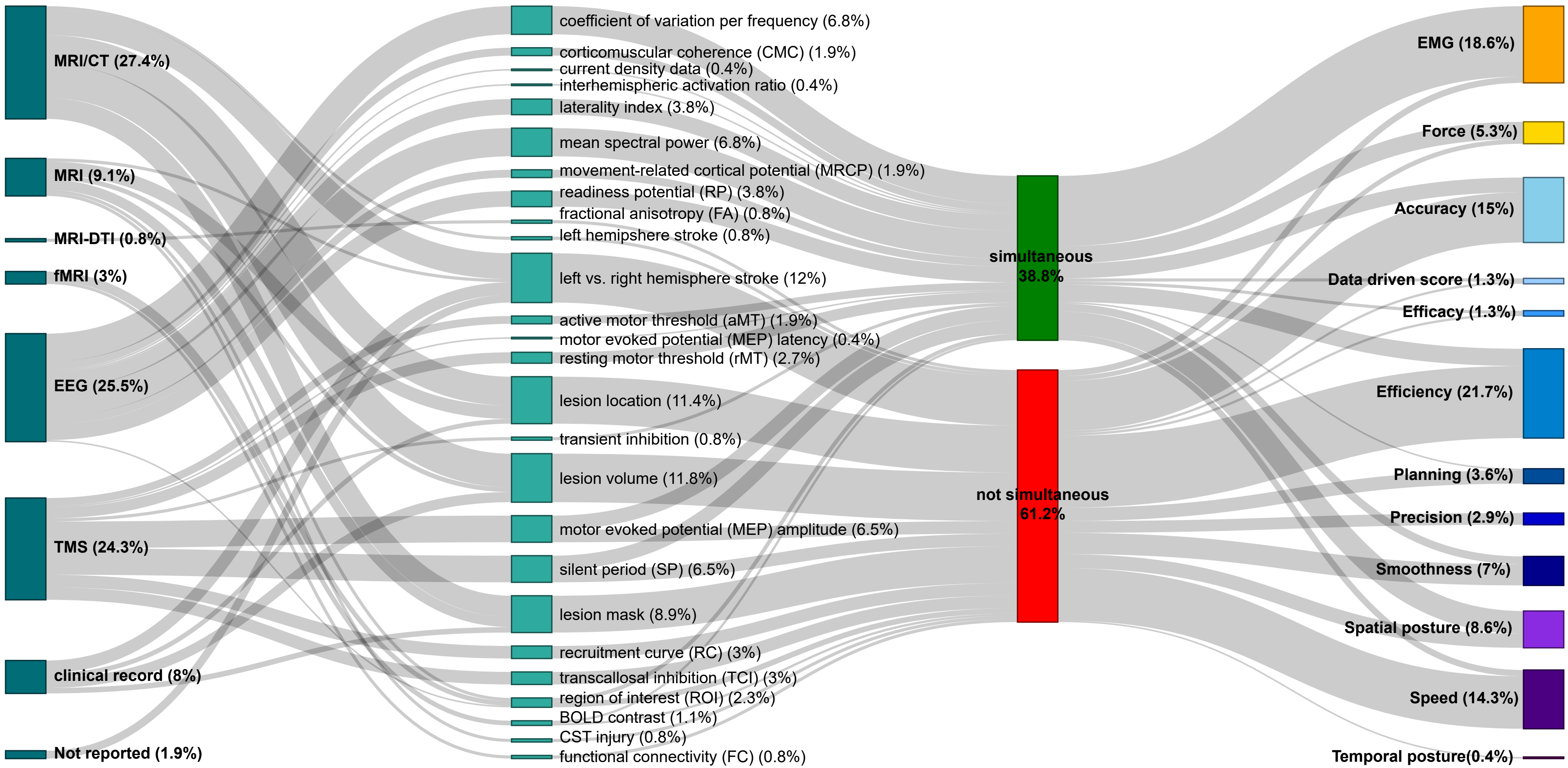

Legend: CST, corticospinal tract; CT, computer tomography, DTI, diffusion tensor imaging; EEG, electroencephalogram; EMG, electromyography; fMRI, functional magnetic resonance imaging; MRI, magnetic resonance imaging; tACS, transcranial alternating current stimulation; tDCS, transcranial direct current stimulation; TMS, transcranial magnetic stimulation. Connections represent co-occurrence of brain-related and UL movement-related measures within the same study. Links do not indicate that one measure was derived from another but reflect multimodal assessment within individual studies.

SM Figure 4C. Frequency of upper limb metrics in studies on reach tasks

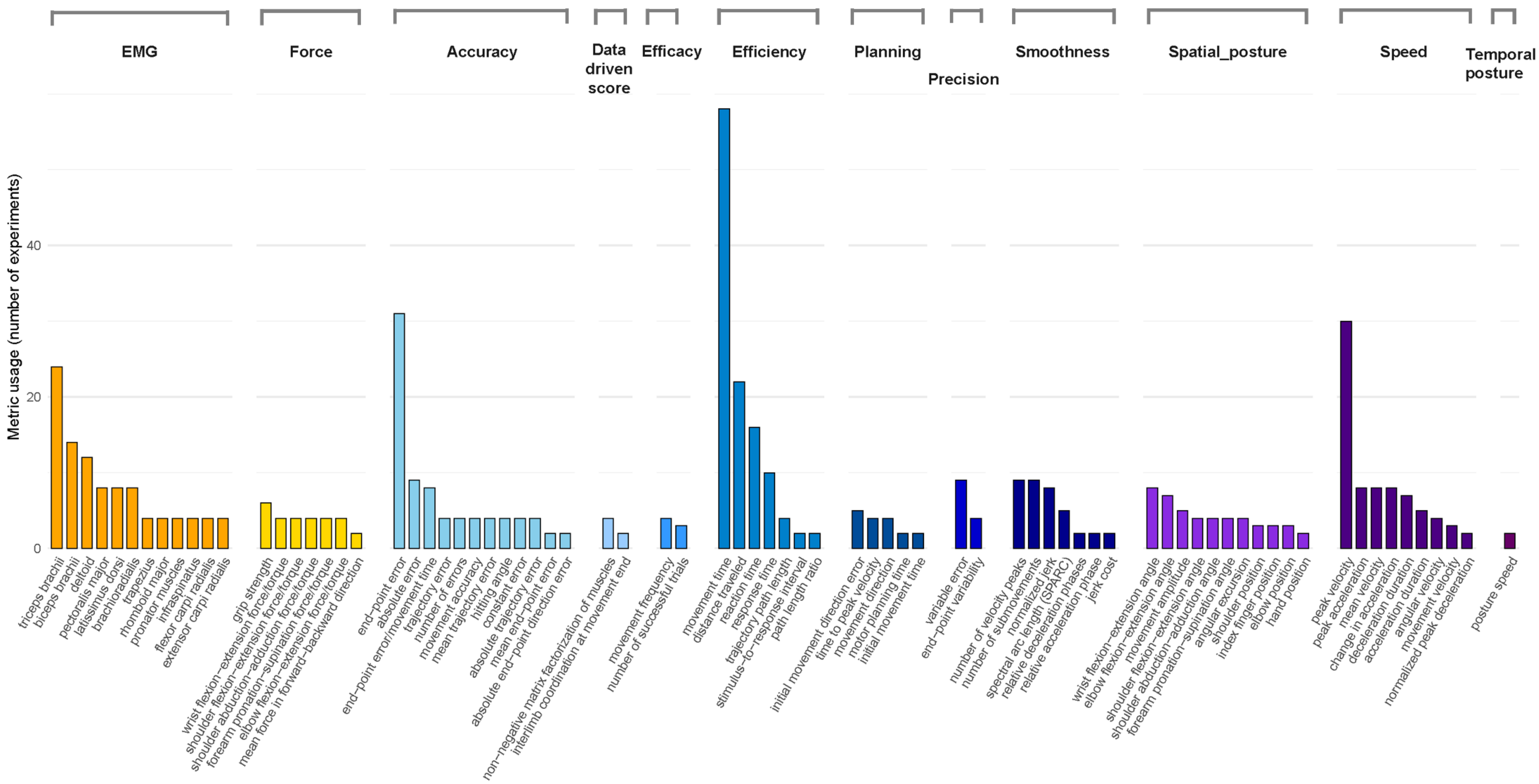

SM Figure 5A. Brain regions reported (including facilitation/activation or inhibition/damage) in studies on grasp tasks

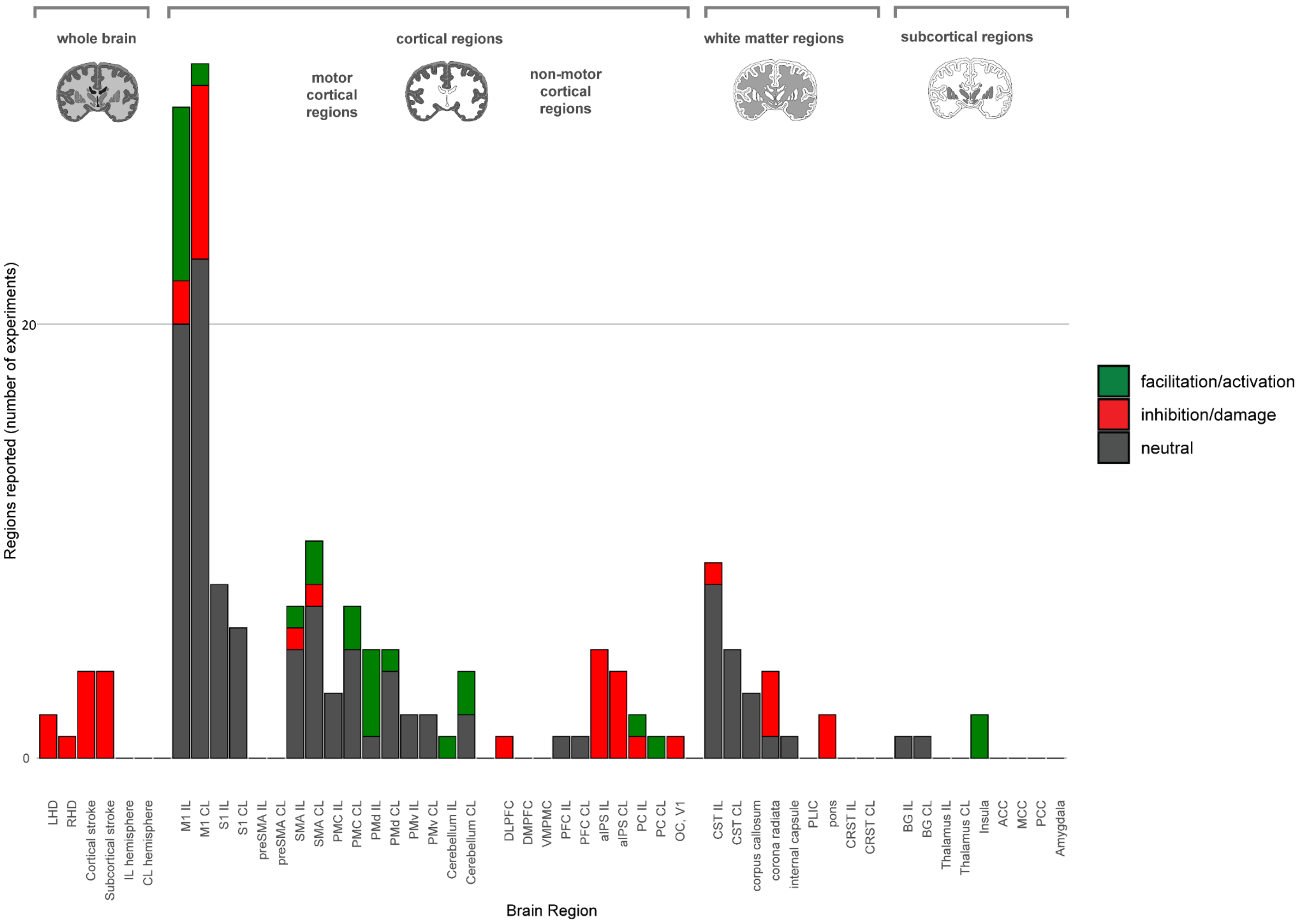

Legend: Green parts of the barplot indicate experiments, where the region of interest was stimulated or showed activations by means of brain-related metrics (e.g. motor evoked potentials, ffunctional magnetic resonance imaging related contrasts), red parts indicate experiments, where the considered region of interest was damaged by the stroke lesion or showed decreased activation or inhibition, and the dark grey parts refer to experiments, where a region was mentioned in the methods but not further specified or did not show significant results and was therefore considered neutral. ACC, anterior cingulate cortex; aIPS, anterior intraparietal sulcus; BG, basal ganglia; CL, contralesional; CRT, corticoreticulospinal tract; CST, corticospinal tract; DLPFC, dorsolateral prefrontal cortex; DMPFC, dorsomedial prefrontal cortex; IL, ipsilesional; LHD, left hemispheric stroke; M1, primary motor cortex; MCC, midcingulate cortex; OC, occipital cortex; PC, parietal cortex; PCC, posterior cingulate cortex; PFC, prefrontal cortex; PLIC, posterior limb of the interal capsule, PMC, premotor cortex; PMd, dorsal premotor cortex; PMv, ventral premotor cortex; preSMA, pre-supplementary motor area; RHD, right hemispheric stroke; S1, primary sensory cortex; SMA, supplementary motor area; VMPFC, ventromedial prefrontal cortex.

SM Figure 5B. Combinations of brain-related and upper limb movement measures in studies on grasp tasks

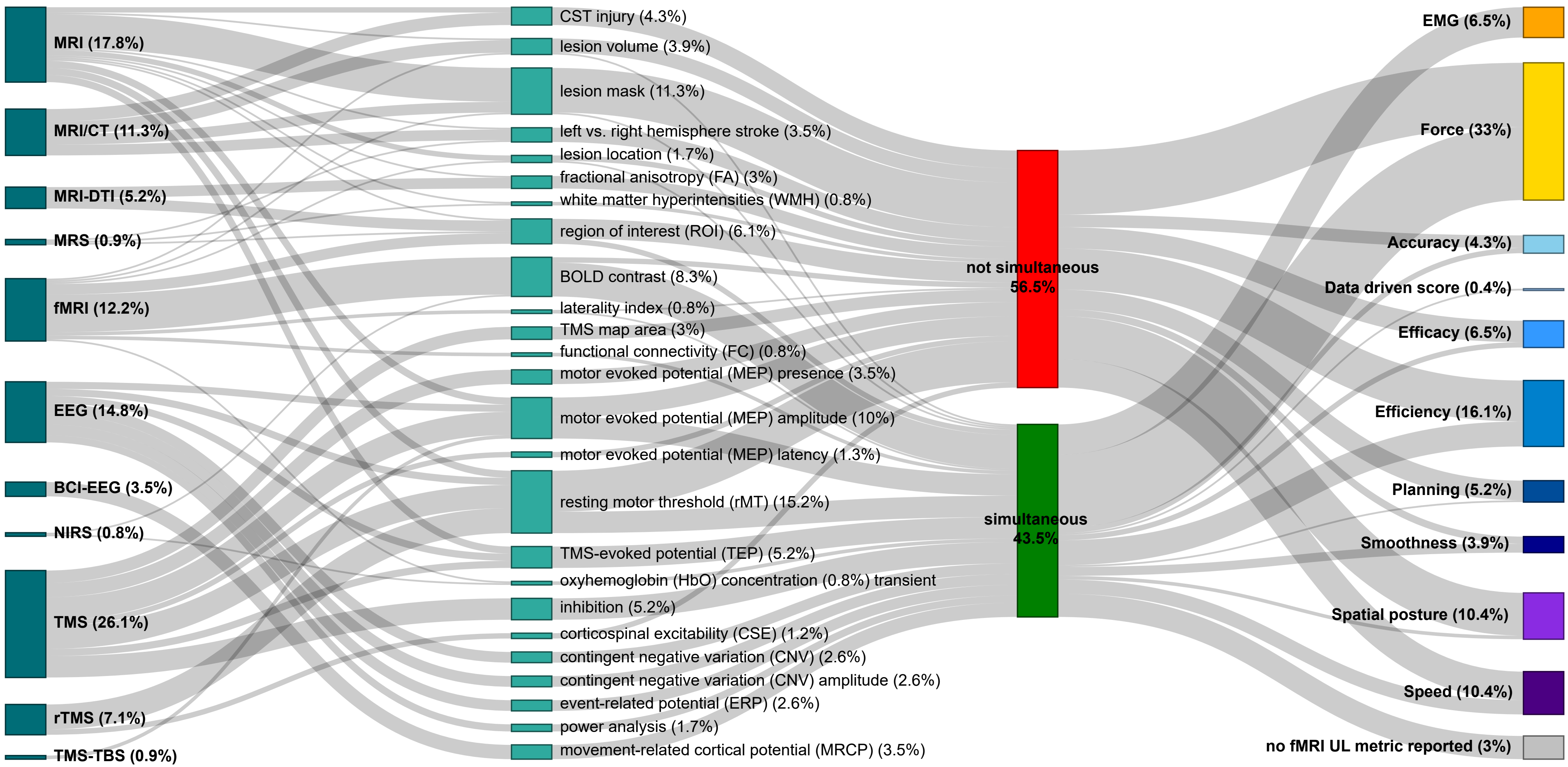

Legend: BCI, brain computer interface; CST, corticospinal tract; CT, computer tomography, DTI, diffusion tensor imaging; EEG, electroencephalogram; EMG, electromyography; fMRI, functional magnetic resonance imaging; MRI, magnetic resonance imaging; MRS, magnetic resonance spectroscopy; NIRS, near-infrared spectroscopy; rTMS, repetitive transcranial magnetic stimulation; TBS, theta burst stimulation; TMS, transcranial magnetic stimulation.

Connections represent co-occurrence of brain-related and UL movement-related measures within the same study. Links do not indicate that one measure was derived from another but reflect multimodal assessment within individual studies.

SM Figure 5C. Frequency of upper limb metrics in studies on grasp tasks

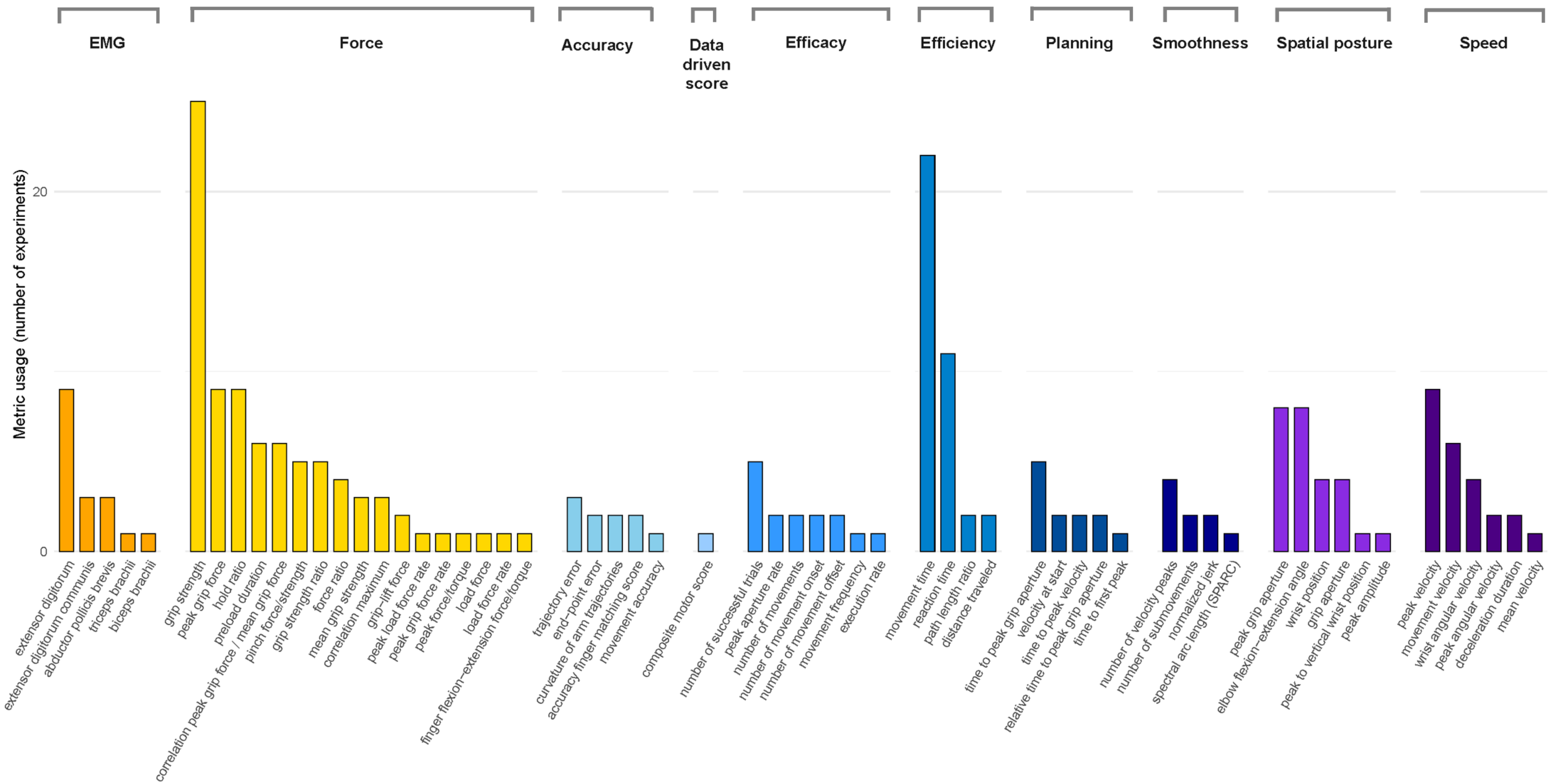

SM Figure 6A. Brain regions reported (including facilitation/activation or inhibition/damage) in studies on complex tasks

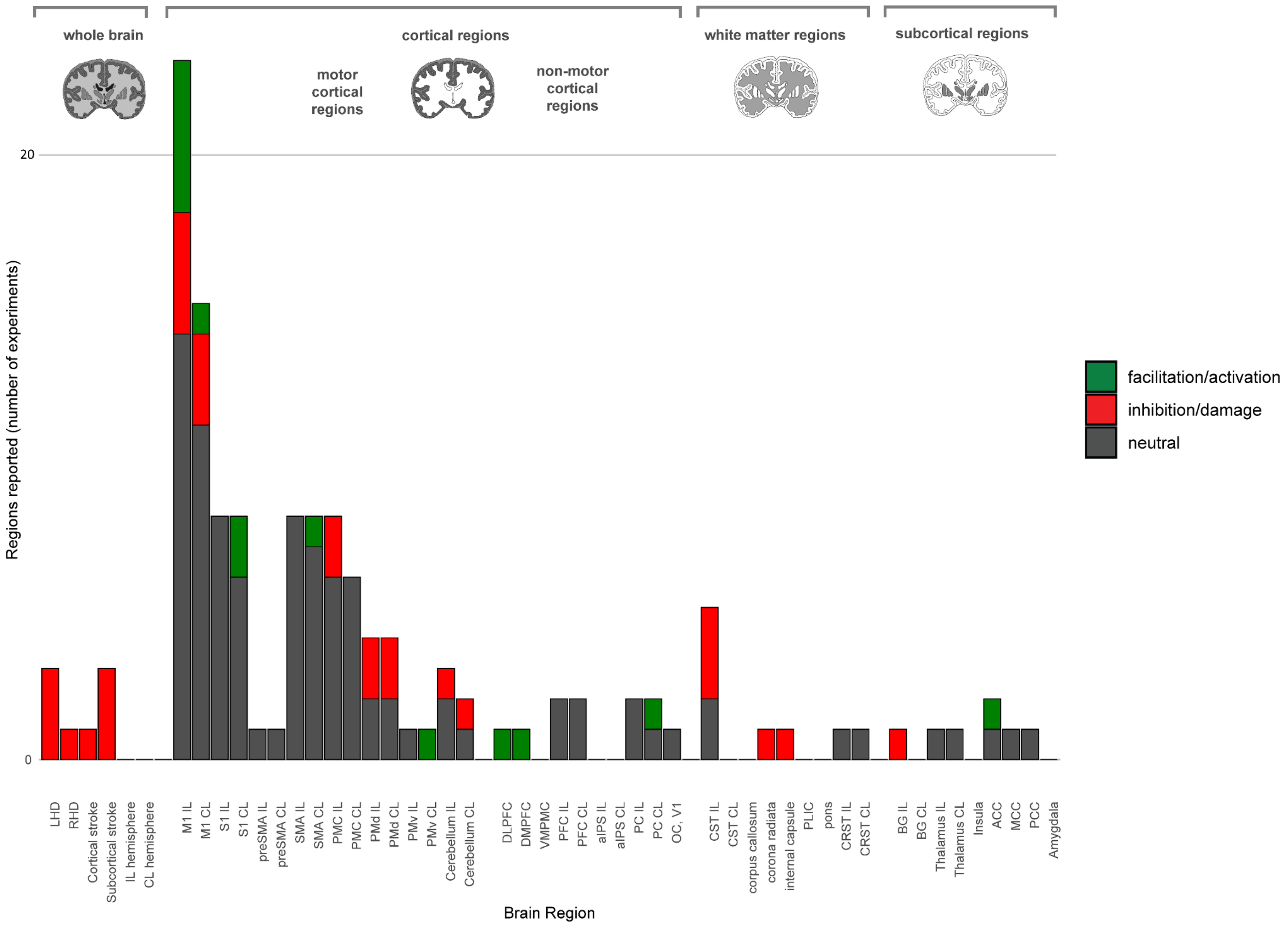

Legend: Green parts of the barplot indicate experiments, where the region of interest was stimulated or showed activations by means of brain-related metrics (e.g. motor evoked potentials, functional magnetic resonance imaging related contrasts), red parts indicate experiments, where the considered region of interest was damaged by the stroke lesion or showed decreased activation or inhibition, and the dark grey parts refer to experiments, where a region was mentioned in the methods but not further specified or did not show significant results and was therefore considered neutral. ACC, anterior cingulate cortex; aIPS, anterior intraparietal sulcus; BG, basal ganglia; CL, contralesional; CRT, corticoreticulospinal tract; CST, corticospinal tract; DLPFC, dorsolateral prefrontal cortex; DMPFC, dorsomedial prefrontal cortex; IL, ipsilesional; LHD, left hemispheric stroke; M1, primary motor cortex; MCC, midcingulate cortex; OC, occipital cortex; PC, parietal cortex; PCC, posterior cingulate cortex; PFC, prefrontal cortex; PLIC, posterior limb of the interal capsule, PMC, premotor cortex; PMd, dorsal premotor cortex; PMv, ventral premotor cortex; preSMA, pre-supplementary motor area; RHD, right hemispheric stroke; S1, primary sensory cortex; SMA, supplementary motor area; VMPFC, ventromedial prefrontal cortex.

SM Figure 6B. Combinations of brain-related and upper limb movement measures in studies on complex tasks

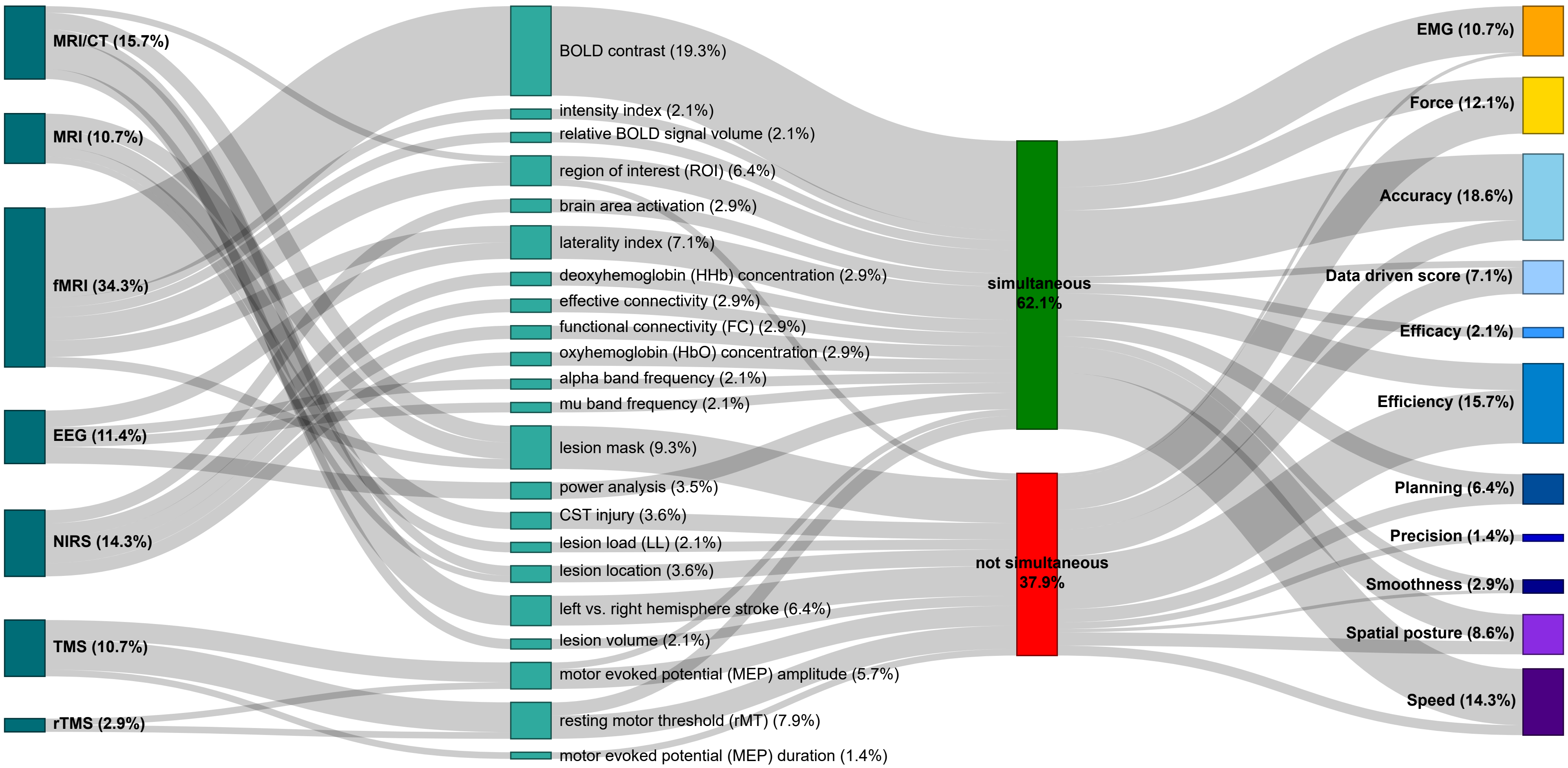

Legend: CST, corticospinal tract; CT, computer tomography, DWI, diffusion weighted imaging; EEG, electroencephalogram; EMG, electromyography; fPET, functional positron emission tomography; fMRI, functional magnetic resonance imaging; MRI, magnetic resonance imaging; NIRS, near-infrared spectroscopy; rTMS, repetitive transcranial magnetic stimulation; TBS, theta burst stimulation; tDCS, transcranial direct current stimulation; TMS, transcranial magnetic stimulation.
Connections represent co-occurrence of brain-related and UL movement-related measures within the same study. Links do not indicate that one measure was derived from another but reflect multimodal assessment within individual studies.

SM Figure 6C. Frequency of upper limb metrics in studies on complex tasks

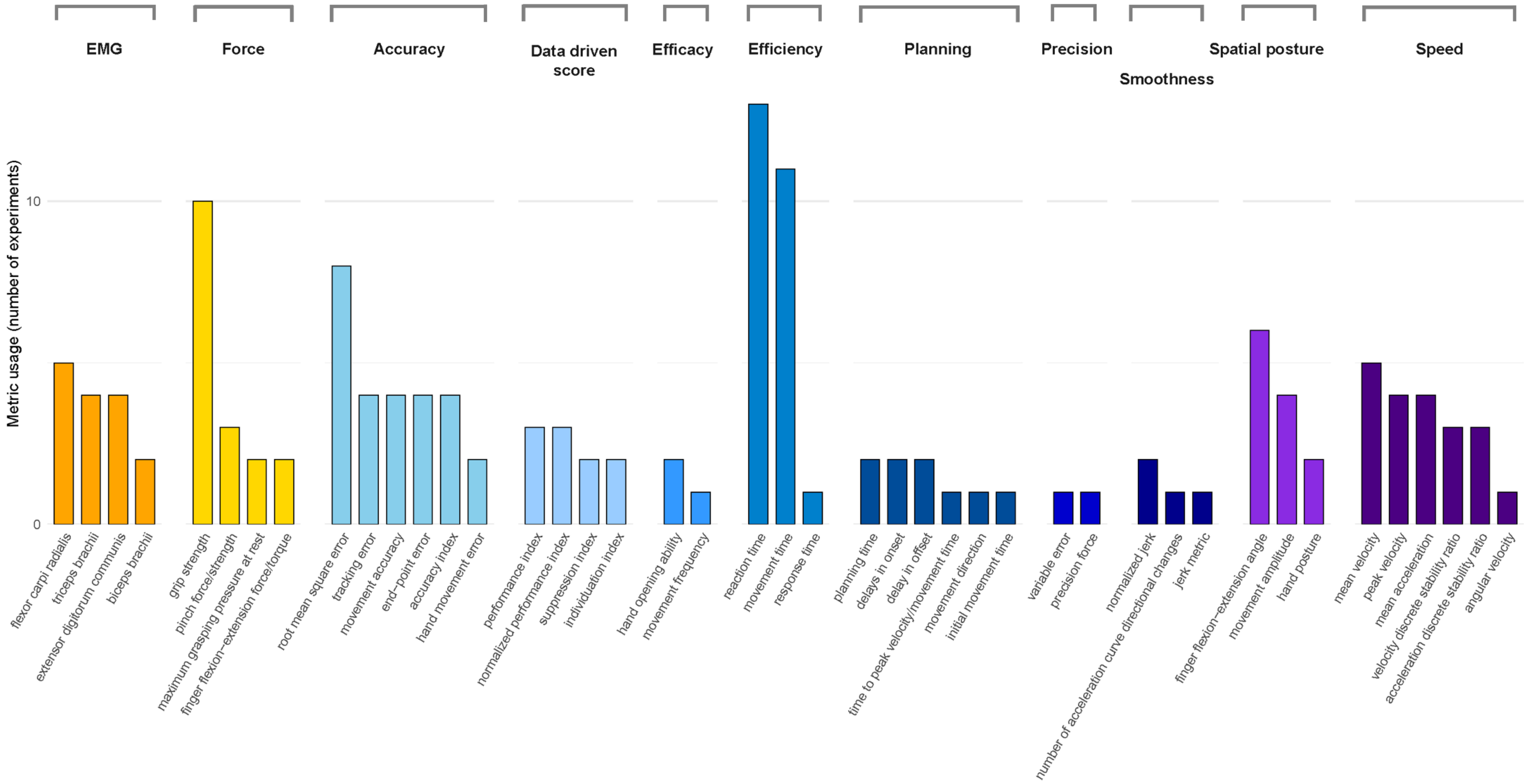

2c. SM Table 1. Study characteristics

First Author, Year	N (N_S/N_H)	Gender (female male)	Age in years, mean ±SD	Time since stroke	Clinical scales	FMA-UE and ARAT	Motor task group and description	Brain-related measurements			Upper limb related measurements			Timing of measures	Study type
								Brain measure system	Brain device	Brain-related metric	UL System influence category/ type	UL device	UL metric		
**Ackerley, 2010(6)**	10 (10/0)	S: 7/3	S: 60±11	**Chronic** >6 months	ARAT	FMA: n.a. ARAT: cTBS 46; iTBS 44; sham 43	**Grasp tasks** custom grip-lift device in pinch task	TMS-TBS	biphasic stimulator (MagStim, Dyfed, UK)	MEP presence	medium / force sensor	instrumented manipulandum	grip strength, load force	not simultaneous	I-L
**Ackerley, 2014(7)**	13 (13/0)	S: 6/6	S: 69 ±8	**Chronic** >6 months	ARAT	FMA: n.a. ARAT: 38 (6-57)	**Strength tasks** custom grip-lift device in pinch task	TMS-TBS	Magstim 200 magnetic stimulator (Magstim, Dyfed, UK)	MEP presence	medium / force sensor	force transducers (MLP-100, Transducer Techniques, Temecula, CA)	preload force, preload duration, precision grip	not simultaneous	I-L
**Altenmüller, 2009(8)**	62 (62/0)	S_E 55.7 ±12.3, S_C 53 ±11.8	S_E 16/16, S_C 6/24	**Early subacute** 1.9 ±1.3 months	ARAT, BBT, NHPT	FMA: n.a. ARAT: S_E 33.3 ±23.9 E_C 36.4 ±23.3	**aROM tasks** Finger and hand tapping, pronation-supination, point-to-point movement	EEG	26 electrodes embedded in elastic cap	ERD	minimal / button press/touch screen	musical instrument digital interface - piano, computerized movement analysis system (CMS 50)	movement frequency, number of velocity peaks, peak angular velocity, peak velocity	simultaneous	I-L
**Ambron, 2018(9)**	37 (25/12)	S: 14/11, H: not reported	not reported	**Chronic** >6 months	FMA-UE	FMA: 15-57 ARAT: n.a.	**Grasp tasks** reach and grasp, grip strength, finger tapping, finger matching	MRI/CT	not reported	lesion mask	minimal / sensor-based medium / grip device	3Delectromagnetic tracking system, (trackSTAR, Ascension Technologies), digital handgrip dynamometer (CAMRY 90 kg)	movement time, time to peak grip aperture, grip strength	not simultaneous	I-L
**Ameli, 2009(10)**	29 (29/0)	S:13/16	S: 56	**Late subacute** 22 ±26 (1-88) weeks	ARAT, MRC, mRS, NIHSS	FMA: n.a. ARAT: 27-57	**aROM tasks** index finger tapping and hand tapping, supination, pronation	fMRI rTMS	1.5T Philips scanner Magstim Super Rapid stimulator (Magstim Company, Dyfed, UK)	BOLD contrast, MEP presence, cortical vs. subcortical stroke	minimal / sensor-based	observer counted (MRI) ultrasonic motion analyzer	movement frequency, peak amplitude	simultaneous	I-C
**Anens, 2010(11)**	18 (7/11)	S: 4/3, H: "similar"	S 38.7 (range 22-48); H 37.3 (range 22-50)	**Late subacute** 3 months	PPT	FMA: n.a. ARAT: n.a.	**Strength tasks** pinch grip to restrain a moving handle	MRI/CT	not reported	cerebellar stroke	medium / force sensor	multiple-element strain gauge transducer system at each beam (0–120 Hz) load force and grip force at 2 plates as orthogonal components (DC 120 Hz).	grip strength, load force, execution rate	not simultaneous	O-C

**Archer, 2018(12)**	30 (15/15)	3//12	61.73 ± 12.90	**Chronic** > 6 months	FMA-UE	FMA: 44.1 ±18.4 ARAT: n.a.	**Strength tasks** pinch force task (15% MVC)	MRI, fMRI	Phillips 3T magnetic resonance scanner (Achieva, Best, the Netherlands) with 32 channel head coil	BOLD contrast, lesion volume, center of mass coordinates	medium / force sensor	custom fiber-optic force transducer with a resolution of 0.025 N (Neuroimaging Solutions, Gainesville, FL)	pinch force/strength, force error	simultaneous	O-C
**Barker, 2012(13)**	39 (28/11)	S: 8/20, C: 6/5	S 66 (12); C 30 (8)	**Chronic** > 6 months	MAS	FMA: n.a. ARAT: n.a.	**aROM tasks** elbow extension	TMS	Stimulation (Magstim 200)	MEP presence, MEP amplitude	medium / force sensor	sEMG, push against a SMART Arm device instrumented to measure elbow extension (‘reach’) force	elbow flexion-extension force/torque	simultaneous	I-L
**Barlow, 2020(14)**	32 (7/25)	S: 0/8, C: 0/25	S: 46.7 ±20.1, C: 30 ±14.9	**Chronic** Mean 70 months	FMA-UE	FMA: 44.9 (24-66) ARAT: n.a.	**Strength tasks** finger pinch (right and left hand) with ramp and hold and lower lip compression	MRI	3T anatomical MRI, T1-weighted image	MCA infarct vs. healthy control	medium / force sensor	forceWIN10 with wireless strain gage sensors for thumb index pinch force	peak force/torque, end-point error, reaction time, end-point variability	not simultaneous	O-C
**Bashir, 2016(15)**	16 (8/8)	S: 4/4, H: 5/3	S 67.5 ±13.8 H 57.4 ±9.6	**Chronic** > 2 years	NHPT	FMA: n.a. ARAT: n.a.	**aROM tasks** finger tapping and reaction time tasks	TMS	Nexstim TMS coil type (201514P) for monophasic pulses and (201383P) for biphasic pulses and navigation software (eXimia 3.1, Nexstim Ltd, Helsinki Finland)	motor cortex excitability contralateral	minimal / button press/touch screen	Pinch key, tap a button, on screen stimulus	pinch force/strength, number of taps, reaction time	simultaneous	I-C
**Bensmail, 2012(16)**	18 (18/0)	S: 3/15	57±14	**Early subacute** 3-330 days	ARAT, MRC	FMA: n.a. ARAT: 53.6±5.7	**Strength tasks** grip-lift task (thumb and index) 5cm for 4s, visually trialed	not reported	not reported	left vs. right hemisphere stroke	medium / force sensor	cylindrical cordless instrumented object (diameter 9 cm, width 3 cm, empty - filled 200g) grip forces (0–80 N) linear accelerations (50 m/s2).	peak grip force rate, peak grip force, peak lift force, grip strength ratio	not simultaneous	O-C
**Bestmann, 2010(17)**	12 (12/0)	S: 2/10	57.4 ± 11.6	**Late subacute** 4-104 months	NHPT, ARAT, MI	FMA: n.a. ARAT: 48 ± 14.5	**Strength tasks** hand grip tests, eg 20% MVC	fMRI paired-coil TMS	1.5T Magnetom Sonata system (Siemens Medical Solutions) MAGSTIM 200 stimulators (Magstim)	FC rMT	medium / grip device	MRI-compatible grip force manipulandum	peak force/torque, number of movement onset, movement time	simultaneous	O-C

**Bhagat, 2020(18)**	10 (10/0)	S: 3/7	S 54.9 ±9.2	**Late subacute** >3months	FMA-UE, ARAT	FMA: 37.8 ±11.7 ARAT: 25.3 ± 16.8	**GraspTask** center-out reaching task on a computer screen to train elbow flex-ext	BCI-EEG	not reported	MRCP amplitude, MRCP latency	minimal / sEMG high / robot-based	MAHI Exo-II exoskeleton (Fitle et al., 2015)	biceps brachii, triceps brachii, grip strength, pinch force/strength, time to first peak, spectral arc length (SPARC), number of velocity peaks, mean velocity	simultaneous	O-C
**Bolognini, 2015(19)**	12 (6/6)	S: 2/4, H: 5/1	S 72.16 ±7.2, H 66.7 ±6.68	**Early subacute** 1-55 months	JTHF	FMA: n.a. ARAT: n.a.	**Complex tasks** 12 of 24 gestures given by experimenter (specific finger positions)	MRI, tDCS	not reported	ROI, lesion mask	minimal / camera-based	video-recorded for offline analysis	movement time, planning time	not simultaneous	I-L
**Bolognini, 2020(20)**	32 (32/0)	not reported	S: 18-80	**Acute** 48-72 hrs	NIHSS, MI-UE	FMA: n.a. ARAT: n.a.	**Strength tasks** hand grip strength	MRI, tDCS	Battery-driven constant current stimulator (BrainStim; EMS, Bologna, Italy) through a saline soaked pair of sponge electrodes (7 x 9 x 5 cm, 35 cm2)	lesion mask, lesion volume	medium / grip device	hydraulic hand dynamometer	grip strength	not simultaneous	I-L
**Bönstrup, 2018(21)**	49 (30/19)	S: 11/19, H: 9/10	S 65 ±13, H 64.8 ±11.1	**Late subacute** 104 ± 17 days	FMA-UE, NHPT	FMA: 46 ±21 ARAT: n.a.	**Strength tasks** grip task, visually guided, isometric	EEG	63 cephalic active surface electrodes, referenced to a nose-tip or Cz-electrode during recording (interim replacement of recording setup)	power analysis, task-related power, task-related coherence, alpha band frequency, beta band frequency	medium / grip device	grip-force device (Grip Force Bimanual, Current Designs, Inc., Philadelphia, USA)	grip strength	simultaneous	O-C
**Brandauer, 2012(22)**	58 (20/38)	S: 4/16	S 57.5 ±10.1	**Chronic** 5.4 ± 2.6years	SARA	FMA: n.a. ARAT: n.a.	**Complex tasks** finger individuation task	MRI	1.5T Siemens Espree MR scanner (Erlangen, Germany)	lesion mask (used on VLSM), cerebellar stroke	medium / end-effector	a custom-made instrumented object with five force sensors	finger flexion-extension force/torque, individuation index	not simultaneous	O-C
**Bu, 2023(23)**	30 (10/20)	not reported	S: 60.95 +/- 6.42 ; H: 61.05 +/- 9	not reported	MoCA, MMSE	FMA: n.a. ARAT: n.a.	**Complex tasks** hold insertion bar and click (stick grip, light click)	fNIRS	fNIRS system (Nirsmart; Danyang Huichuang Medical Equipment Co, Ltd, PR China)	brain area activation, FC, effective connectivity	minimal / camera-based	camera-based with one marker	mean velocity, mean acceleration, acceleration discrete stability ratio, velocity discrete stability ratio	simultaneous	O-C
**Buckingham, 2018(24)**	19 (1/18)	S: 1/0, H: 12/6	S 43, H: 42.4	**Chronic** >14 years	not reported	FMA: n.a. ARAT: n.a.	**Grasp tasks** grasp and lift with thumb and index, 6 constant weights and	MRI	not reported	occipital stroke	medium / force sensor	handle contained a single ATI Nano17 force transducer	peak load force rate, load force rate, peak grip force rate	not simultaneous	O-C

							sizes from 5-15cm								
**Buetefisch, 2018(25)**	48 (18/30)	S: 8/10, H: not reported	S: 61.8 ±11.9, H wrist: 62.9 ±7 H TMS: 61.3 ±5.5	**Chronic** > 6 months	MAL JTHF	FMA: n.a. ARAT: n.a.	**aROM tasks** Wrist extension as fast as possible with auditory cue	TMS	two Magstim stimulators connected via a Bistim module (Magstim), Siemens 3T Trio scanner using a 12-channel head coil	M1 thickness, FA, motor MEP amplitude, SICI	minimal / sensor-based, sEMG	hand accelerometer, EMG of ECU	grip strength, reaction time, wrist flexion-extension angle, peak wrist acceleration	not simultaneous	O-C
**Buma, 2016(26)**	17 (17/0)	S: 3/14	S 59.9 ±12.6	**Early subacute** 6 weeks and 29 weeks	FMA-UE, ARAT, NHPT	FMA: 55 ±12.7 ARAT: 41.2 ±14.5	**Grasp tasks** finger-flexion extension during fMRI reach-to-grasp a block	fMRI	Philips Achieva 3T MRI scanners (Philips, Eindhoven, Netherlands)	BOLD contrast, ROI, FC	minimal / sEMG sensor-based	EMG electromagnetic motion-tracking device (Polhemus Liberty, Polhemus, Vermont)	extensor digitorum communis, abductor pollicis brevis movement time, normalized jerk	simultaneous not simultaneous	O-L
**Bundy, 2017(27)**	10 (10/0)	not reported	S 58.6 ±10.3	**Chronic** > 6 months	ARAT, COPM, MI, mAS	FMA: n.a. ARAT: 13.4 ±10.1	**aROM tasks** hand opening and closing (rest, unaffected, affected hand or bilateral motion)	BCI-EEG	8 channels g.LadyBird active electrodes system and g.Mobilab+ EEG amplifier (g.Tec, Graz, Austria)	power analysis	high / robot-based	powered exoskeleton with 3finger pinch opening and closing	grip strength, pinch force/strength, finger flexion-extension angle	simultaneous	I-L
**Buxbaum, 2005(28)**	19 (13/6)	S1: 6/2, S2: 2/3, H: 4/2	S1 53.6 ±13.6, S2 53.4 ±10.8, H 54.0 ± 7.9	**Chronic** > 6months	Western aphasia battery	FMA: n.a. ARAT: n.a.	**Grasp tasks** motor control task: finger dexterity, grip strength (left vs right in pounds)	MRI	not reported	lesion mask	minimal / camera-based	videotapes	movement accuracy, number of successful trials, peak grip aperture	not simultaneous	O-C
**Calabrò, 2016(29)**	35 (35/0)	H: 13/22	51.1 ±11	**Early subacute** > 2 months	FMA-UE, mAS	FMA: 20 ±1.5 ARAT: n.a.	**Reach tasks** pointing in 3D space	TMS	high-power Magstim 200 stimulator (Magstim, Dyfed, United Kingdom [UK])	MEP amplitude, rMT, aMT, SP	high / robot-based	Armeo Power 3D exeskeleton (Hocoma AG, Volketswil, Switzerland)	shoulder flex-ext force/torque, shoulder abd-add force/torque, elbow flex-ext force/torque, forearm pro-sup force/torque, wrist flex-ext force/torque, shoulder flex-ext angle, shoulder abd-add angle, elbow flex-ext angle, forearm pro-sup angle, wrist flex-ext angle	simultaneous	I-L
**Cantillo-Negrete, 2021(30)**	10 (10/0)	S: 5/5	59.9 ±12.8	**Early subacute** <2month	FMA-UE, ARAT	FMA: 17.5 ±15.3, ARAT: 4.3 ±6.4	**aROM tasks** motor imagination to trigger finger flexion-extension via an exoskelet	BCI-EEG, TMS	11 active electrodes (g.LadyBird, g.tec) Rapid2 MAGSTIM device	power analysis, ERD, ERS, rMT	medium / grip device	Dynamometer Biometrics E-link evaluation system	pinch force/strength, grip strength	not simultaneous	I-L

**Carey, 2002(31)**	19 (10/9)	S: 4/6	65.7 ± 13.3	**Chronic** 4.7 ±6.3years	BBT	FMA: n.a. ARAT: n.a.	**Complex tasks** Finger tracking test	fMRI	4T whole body magnet (Oxford UK) with Unity INOVA console (Varian, California, USA), body gradient insert (Siemens, Germany)	BOLD contrast, laterality index	minimal / sensor-based	potentiometer (Waters Manufacturing and Company, Wayland, Mass., USA) on index MCP	tracking error, finger flexion-extension angle	simultaneous	I-L
**Carey, 2007(32)**	20 (20/0)	E: 1/9, C: 4/6	E 65.9 ±7.4, C 67.4 ±11.8	**Chronic** E 42.5 ±24.3, C:35.6 ±26.1 months	BBT, JTHF	FMA: n.a. ARAT: n.a.	**Complex tasks** finger tracking	fMRI	3T magnet (Magnetom Trio, Siemens, Munich, Germany)	BOLD contrast, intensity index	minimal / button press / touch screen	custom-made electrogoniometer braces with 2 potentiometers at MP and wrist	accuracy index, root mean square error, finger flexion-extension angle	simultaneous	I-L
**Carino-Escobar, 2021(33)**	10 (10/0)	S: 5/5	S 59.9 ±12.8	**Late subacute** 4.6 ± 2.7 months	FMA-UE, ARAT	FMA: 12.5 (IQR=8) ARAT: 3 (IQR = 6)	**aROM tasks** Flexion and extension of paralyzed fingers (gripping and pinching task, grabbing objects in ARAT task)	EEG, TMS	g.USBamp biosignal acquisition unit and g.LADYbird 11 active electrodes Magstim Rapid2	ERD, ERS, coherence, rMT	medium / grip device	Dynamometer	grip strength, pinch force/strength	simultaneous	O-L
**Chalard, 2020(34)**	24 (15/9)	S: 4/11, C: 3/6	S 55 ±11, C:43 ±21	**Chronic** > 6 months	FMA-UE	FMA: 38 ± 9 ARAT: n.a.	**aROM tasks** elbow extension and flexion task	EEG	64 channel ActiveTwo system (BioSemi instrumentation, Amsterdam, The Netherlands)	ERD, beta band frequency, power analysis	minimal / sEMG camera-based	Ag-AgCl bipolar electrode with MP150 system and EMG100C amplifiers (Biopac Systems Inc., Goleta, CA, USA) eight OptiTrack infrared cameras	triceps brachii, biceps brachii, brachioradialis, brachialis movement time, normalized jerk, elbow flexion-extension angle, elbow angular velocity	simultaneous	O-C
**Chau, 2009(35)**	18 (18/0)	S: 7/11	S 67.8 ±8	**Early subacute** 22 ±6.1 days	FMA-UE, BI	FMA: 43.9 ± 27.2 after, 20.3 ± 15.6 before ARAT: n.a.	**Strength tasks** squeeze a rubber ball	fMRI	3T MRI scanner (MagnetomTrio Tim syngo, SiemensMedical Solutions, Erlangen, Germany)	ROI	medium / grip device	rubber ball, not further specified dynamometer	no metric reported (fMRI) grip strength	simultaneous	I-L
**Chen, 2022(36)**	36 (36/0)	S1: 8/10, S2: 8/10	S1 57.8 ±8.4, S2 58.4 ±9.3	**Early subacute** 1-3 months	FMA-UE, ARAT, NIHSS	FMA: S1 26.3 ± 6.0, S2 26.0 ± 7.1 ARAT: S1 18.6 ± 6.0, S2 18.3±5.2	**Grasp tasks** VR reaching and reach to grasp (fried egg, apple picking) and grip-target-release task (archery)	EEG	32-channel QuickAmp amplifier Ag/AgCl scalp electrodes (Brain Products, Germany).	CNV, ERP, CNV amplitude	minimal / sEMG, high / robot-based	EMG on extensor digitorum, grip sensor embedded in handlebar of exeoskelet with weight support at elbow and wrist to detect grip force.	extensor digitorum, reaction time	simultaneous	I-L

**Cheng, 2021(37)**	30 (30/0)	S: 11/19	S 62.3 ± 16.9	**Acute** 3-5 days	NIHSS	FMA: n.a. ARAT: n.a.	**Strength tasks** Strength JAMAR hand evaluation kit	MRI	32-channel head coil for DWI and high-resolution T1-weighted anatomical images	fixel based analysis (FBA)	medium / grip device	Jamar dynamometer	grip strength	not simultaneous	O-C
**Cirstea, 2018(38)**	10 (10/0)	S: 2/8	S 58.7 ±6.8	**Chronic** > 6months	FMA-UE	FMA: 35.6±18.6 ARAT: n.a.	**Grasp tasks** reach to grasp cylindrical objects	MRI, H-MRS	Siemens 3T Allegra MR system (Siemens Medical Solutions, Erlangen, Germany)	ROI, left vs. right hemisphere stroke, white matter hyperintensities	minimal / camera-based	VICON, Oxford Metrics with 5 markers, electrogoniometer (Exos, Inc., Woburn, MA, USA)	elbow flexion-extension angle	not simultaneous	O-C
**Classen, 1997(39)**	32 (16/16)	S: 8/8, H: 8/8	S: 62 ±13, C: 60 ±15	**Acute** ≤1 week	clinical descriptions	FMA: n.a. ARAT: n.a.	**aROM tasks** finger tapping and isometric contraction	TMS	Magstim 200 (Madaus, Freiburg, Germany) connected to circular coil (12 cm diameter)	MEP amplitude, SP, lesion mask	minimal / sEMG, medium / strain gauge	EMG, strain-gauge (range 0-100N, non-linearity <1%, contact surface area 0.7x1.8 cm2) for index finger abduction	first dorsal interosseous, % MVC	simultaneous	O-L
**Cramer, 2002(40)**	30 (11/19)	S: 7/4, H:8/11	S: 58, H: 49	**Late subacute** >3 months	FMA	FMA: 16	**aROM tasks** finger tapping, visuomotor task	fMRI	1.5T scanner	BOLD contrast, PSC, lesion mask	minimal / sEMG medium/ wrist splint and force sensor	not reported	tapping rate, mean muscle activity (pectoralis major, biceps brachii, extensor carpi radialis, extensor carpi ulnaris, flexor carpi radialis, flexor carpi ulnaris, first dorsal interosseus)	simultaneous	O-C
**Dafotakis, 2008(41)**	12 (12/0)	S: 4/8	S 45 ±9	**Early subacute** 1-4 months	ARAT, MRC, MMSE, NIHSS, mRS, SIS	FMA: n.a. ARAT: range 40-57	**Grasp tasks** grip-lift task	rTMS	Magstim Rapid stimulator (Magstim Company, Dyfed, UK)	lesion location, subcortical stroke	minimal / sensor-based	instrumented object (diameter of 9.0 cm and a depth of 4.0 cm, 350g incl. accelerometer and force sensors)	grip strength, pinch force/strength, force ratio	not simultaneous	I-C
**Dawson, 2016(42)**	20 (20/0)	VNS: 2/7, C: 2/9	VNS 57.9 ±17.2; C 60.7 ±10.7	**Chronic** > 6months	FMA-UE, ARAT, NHPT, BBT	FMA: VNS 40.1 ±9.7, C 45.3 ±8.4 ARAT: VNS 32.6 ±7.2, C 34 ±8.1	**Grasp tasks** reaching, gripping, hand wrist motions, grip force control	MRI	3T MRI scanners	lesion volume, LL CST, FA ratio	medium / end-effector	InMotion robot	grip strength	not simultaneous	I-L
**de Paiva Silva, 2014(43)**	30 (20/10)	LHD: 2/8, RHS: 2/8, H: 4/6	LHD 55.1 ± 17.5, RHD 62.9 ± 7, H 51.3 ± 17.4	**Chronic** >6 months, RHD 3.2 ±2.8, LHD 6 ±5.7	FMA-UE	FMA: LHD 42 ±18, RHD 29 ±20 ARAT: n.a.	**Complex tasks** discrete and sequential task grip and pinch strength	not reported	not reported	left vs. right hemisphere stroke, cerebellar stroke, lesion mask, lesion volume	minimal / button press / touch screen, medium / grip device	12-inch digitizing table (Wacom Intuos 2, WA) handgrip (Jamar; Asimow Engineering Co., LA, CA) and pinch (Preston Pinch Gauge; B & L Engineering Co., NJ), dynamometers	reaction time, movement time, time to peak velocity / movement time, initial movement time, variable error, number of acceleration curve directional changes, peak velocity grip strength, pinch force/strength	not simultaneous	O-C

**Delcamp, 2022(44)**	19 (10/9)	S: 2/8, C: 5/4	S 33-76, C: 37 (20-73)	**Chronic** > 6 months	FMA-UE, EmNSA	FMA: 21-53	**aROM tasks** elbow flexion/extension	EEG	not reported	CMC	minimal / camera-based, sEMG	125 Hz OptiTrack, NaturalPoint Inc., Corvallis, OR, USA EMG Biopac Systems Inc	elbow flexion-extension angle, peak velocity, muscle activity of triceps brachii, biceps brachii, brachialis, brachioradialis	simultaneous	I-L
**Di Lazzaro, 2013(45)**	12 (12/0)	S1: 3/3, S2: 2/4	S1 59.5 ± 12.4, S2 57.5 ± 12.3	**Chronic** > 1year	ARAT, NHPT, JTHF, NIHSS, BI	FMA: n.a. ARAT: TBS 21.8 ± 8.6, sham 23.6 ± 10.2	**Strength tasks** grasp and pinch force	TMS, TBS	MagPro stimulator (Medtronic A/S, Denmark) and coil (MCF-B65)	rMT, aMT, MEP amplitude	medium / grip device	digital dynamometer (Biometrics Ltd, Newport, UK)	grip strength, pinch force	simultaneous	I-L
**Dimyan, 2022(46)**	42 (42/0)	S: 15/27	S 59.8 ±11.28	**Chronic** ≥ 6 months	FMA-UE, ARAT, WMFT	FMA: 19.8 ±9.6 ARAT: 14.1 ±14.7	**Strength tasks** grip strength	MRI, TMS	MRI or TMS system not reported	lesion mask, LL CST, FA, MD TMS score	medium / grip device	dynamometry EMG for TMS not reported	first dorsal interosseous, extensor carpi radialis, deltoid, grip strength	not simultaneous	I-L
**Fang, 2007(47)**	20 (12/8)	not reported	S: 56.52±7.73, H: 60.62±6.25	**Chronic** >12 months	not reported	FMA: n.a. ARAT: n.a.	**Reach tasks** circle drawing task	EEG	64-channel NeuroScan EEG system (NeuroScan Labs, El Paso, TX)	MRCP	medium / end-effector	InMotion2 planar robot	triceps brachii, deltoid, movement time, motor planning time, jerk cost	simultaneous	O-C
**Fang, 2009(48)**	29 (21/8)	S: 5/16, H: 3/5	S: 59.57 ±8.61; H 60.62 ±6.26	**Chronic** 12–109 (40.5 ±29.1) months	FMA-UE	FMA: range 15-41	**Reach tasks** planar reaching with MIT manus	EEG	64-channel NeuroScan EEG system (NeuroScan Labs, El Paso, TX)	CMC	medium / end-effector	InMotion2; Interactive Motion Technologies, Inc., Cambridge, MA)	deltoid, biceps brachii, triceps brachii, mean force in forward-backward direction, end-point error	simultaneous	O-C
**Fang, 2015(49)**	15 (10/5)	S 2/8, H: 1/4	S: 63.5 ±12.44, H: 62.4 ±8.91	**Chronic** >12 months	FMA-UE	FMA: range 14-49	**Reach tasks** planar pointing 2D	EEG	64-channel NeuroScan EEG system (NeuroScan Labs, El Paso, TX)	current density data, interhemispheric activation ratio, ROI	medium / end-effector	InMotion2 planar robot	end-point error, elbow flexion-extension angle	simultaneous	O-C
**Ferreiro de Andrade, 2018(50)**	22 (12/10)	S: 6/6, C: not reported	not reported	**Chronic** > 6months	JTHF	FMA: n.a. ARAT: n.a.	**Strength tasks** grip strength	TMS	two magnetic stimulators via a Bi-Stim 2002 module (Magstim, UK)	SICI	medium / grip device	Jamar dynamometer	pinch force/strength	not simultaneous	O-C
**Fisher, 2006(51)**	10 (5/5)	S: 0/5 ; H: 2/3	S: 26-75 ; H: 23-73	**Chronic** 6-90 months	Ataxia rating	FMA: n.a. ARAT: n.a.	**Reach tasks** reach to six locations (2 directions, 3 ampitudes)	MRI	not reported	lesion mask	medium / end-effector	instrumented manipulandum, a light-weight aluminum lever on horizontal plane	average error, number of successful trials, movement time, stimulus-to-response interval, variable error, variance in displacement, mean velocity, peak velocity	not simultaneous	O-C
**Foltys, 2003(52)**	10 (10/0)	S: 5/5	S: 45.6 ±4.3	**Early subacute** 14 ±3.1 days	NIHSS, ESS	FMA: n.a. ARAT: n.a.	**aROM tasks** hand open and close task; finger-thumb opposition	fMRI TMS	1.5T Gyroscan NT-scanner (Philips Medical Systems, Netherlands) Magstim 200 stimulato	BOLD contrast, MEP amplitude	minimal / camera-based	video recording and movement frequency was compared for affected and unaffected hand	movement frequency	simultaneous	O-C

**Frenkel-Toledo, 2014(53)**	38 (38/0)	S: 16/22	S: 55.4 ± 13.7	**Early subacute** 58.7±29. 7 days	FMA-UE, apraxia test	FMA: 38.6 ± 21.2 ARAT: n.a.	**Complex tasks** E-Prime test: left egocentric, right ego-centric, right allocentric, left allocentric	EEG	32 active electrodes mounted on elastic cap (BiosemiTM, Amsterdam, Netherlands)	alpha band frequency, mu band frequency	minimal / sEMG	EMG Ag-Ag Cl sintered pallet of 4mm diameter lying within a 11mm wide area	triceps brachii, flexor carpi radialis, suppression index	simulta neous	O-C
**Fujii, 2003(54)**	76 (46/3 0)	not reporte d	S: 60 (45-75)	**Late subacute** 1-6 months	BS	FMA: n.a. ARAT: n.a.	**Grasp tasks** self-paced grasp task approximately at one grasp/second	fMRI	MR imaging system (Signa; General Electric Medical Systems, Waukesha, WI) with 3T magnet (Magnex, Yarnton, UK)	BOLD contrast	minimal / camera-based	charge-coupled device camera and the grasp rate was recorded for each session	execution rate	simulta neous	O-C
**Gao, 2019(55)**	11 (5/6)	S/H: 11/0	S: 48 ±2.2 H: 24.7 ±1.2	**Early subacute** 1-2 months	not reported	FMA: n.a. ARAT: n.a.	**Strength tasks** Hand Grip (5kg, 10kg, 20kg, MVC)	EEG	32-channel EEG acquisition system (Brain Products GmbH, Germany)	power analysis	medium / grip device	spring grip meter (EH101, Lynx Mall, China). Test three levels of hand grip (5 kg, 10 kg, and 20 kg)	grip strength	simulta neous	O-C
**Garcea, 2019(56)**	29 (21/8)	S: 6/15, C: 4/4	S: 61.5 ±10.1, C: 64.7 ±9.4	**Chronic** >12mont hs	not reported	FMA: n.a. ARAT: n.a.	**Complex tasks** stroop like gesture task (grasp use vs. task)	fMRI MRI	3T (Siemens Trio, Erlangen, Germany)	lesion mask (used in VLSM)	minimal / camera-based	a digital camera and scored offline by two trained	hand movement error, movement amplitude, hand posture	not simulta neous	O-C
**Gera, 2016(57)**	32 (22/1 0)	S: 8/14 H: not reporte d	LHD: 65.4 ± 11.7; RHD, 61 ± 12.1 H: 67 ± 5	**Chronic** 1.9 ±2.1 years	FMA-UE	FMA: RCVA 43.9 ± 9.1, LCVA 47.9 ± 12.5 ARAT: n.a.	**Reach tasks** pointing movements towards screen (3D)	not reported	not reported	left vs. right hemisphere stroke	minimal / camera-based	eight-camera VICON motion measurement system	uncontrolled manifold analysis of elbow and wrist, movement time, hand path variability	not simulta neous	O-C
**Giacobbe, 2013(58)**	12 (12/0)	S: 6/6	S: 64.5 ±11.6	**Chronic** > 6 months	FMA-UE	FMA: 38.3 ± 16.8 ARAT: n.a.	**aROM tasks** wrist motion	TMS	MagPro × 100 stimulator (Mindcare Co.)	MEP presence	medium / end-effect	InMotion3 wrist robot, EMG	flexor carpi radialis, extensor carpi radialis, end-point error, movement time, mean velocity, peak velocity	simulta neous	I-C
**Groisser, 2014(59)**	22 (10/1 2)	S: 5/5, H: not reporte d	not reported	**Acute** 3–7 days	MI, NHPT	FMA: n.a. ARAT: n.a.	**Strength tasks** gripping task	MRI-DTI	Siemens 3T TIM Trio MRI scanner and 12-channel head coil	lesion volume, CST diffusion axial diffusivity, radial diffusivity, FA	medium / grip device	digital dynamometry	grip strength	not simulta neous	O-L
**Haaland, 1989(60)**	60 (29/3 1)	not reporte d	C_R 66.1 ±9.8; C_L 64.8 ±7.1; S_R 65 ±10; S_L 62±5	**Chronic** 19.6 ±24.4 S_R, 26 ±44.6 months S_L	grip (yes/no)	FMA: n.a. ARAT: n.a.	**Reach tasks** arm reaching task	not reported	not reported (CT scans)	left vs. right hemisphere stroke	minimal / button press / touch screen	TV monitor and HI-PAD digitizing table with Stylus	constant error, reaction time, movement time, mean velocity initial component, variable error, movement amplitude, mean velocity	not simulta neous	O-C

**Haaland, 1999(61)**	50 (26/24)	not reported	S_NA 60 ±14; S_A 67 ±7; C 62 ±10	**Chronic** 4.9 non-apraxic, 5.7 years apraxic	not reported	FMA: n.a. ARAT: n.a.	**Reach tasks** planar pointing movements, tapping	MRI/CT	Siemens or Picker 1.5T scanner	lesion volume, lesion mask	medium / end-effector	digitizing tablet with monitor	constant error, reaction time, movement time, distance traveled, initial movement time, variable error, mean velocity	not simultaneous	O-C
**Haaland, 2009(62)**	46 (22/24)	S_1 3/9; S_2 1/9; H 6/18	S_1 65 ±9.4; S_2 55.7 ±12.5; C 62.3 ±8.5	**Chronic** S_1: 5.8 ±4.9 S_2: 7.2 ±5.8 years	FMA-UE	FMA: S_1 61.2 ±3.2; S_2 32.7 ±20.1	**Reach tasks** planar pointing task	MRI/CT	1.5T system (Edge, Picker) or CT scan (Picker)	lesion volume, lesion location	minimal / sensor-based	Flock of Birds (FoB)® (Ascension-Technology) magnetic 6-DOF motion recording system, 103Hz	absolute error, movement time, variable error, normalized peak deceleration, deceleration duration, grip strength	not simultaneous	O-C
**Hammerbeck, 2019(63)**	17 (17/0)	not reported	S: 58.7 ±10.8	**Chronic** < 1 year	FMA-UE	FMA: 47.5 ±11.8	**Reach tasks** reaching forward	TMS	Magstim 200 magnetic stimulator (Magstim Company, Whitland, Dyfed, UK)	MEP latency, MEP presence	high / robot-based	robotic manipulandum, Ag/AgCl electrodes (Skintact)	end-point error, root mean square error	simultaneous	O-C
**Hannanu, 2020(64)**	59 (27/32)	S: 8/19, H: not reported	S: 52, H: 25	**Early subacute** day 31 (27-35)	PPT	FMA: n.a. ARAT: n.a.	**aROM tasks** passive wrist-ext (rest and 45° wrist flex-ext at 1 Hz)	fMRI	3T (Achieva 3.0T TX, Philips, The Netherlands)	BOLD contrast	medium / wrist splint	splint moved by examiner in scanner room, dynamometer	grip strength	simultaneous	O-L
**Hannanu, 2022(65)**	58 (29/29)	S 8/21; H 8/21	S: 52.1 ±9.8; H: 51.1 ±12.2	**Early subacute** 1 month	PPT	FMA: n.a. ARAT: n.a.	**Strength tasks** grip strength	MRI, MRI-DWI	3T Philips magnet (Achieva 3.0 TTX; Philips, the Netherlands) 32-channel head coil	FA	medium / grip device	Dynamometer (Lafayette Instrument Company, Indiana, US)	grip strength, movement time	not simultaneous	O-L
**Hara, 2013(66)**	16 (16/0)	S: 3/13	S: 49 (18-73)	**Chronic** mean interval : 18 months (range 12–24 months)	FMA-UE SIAS	FMA: 24 range 11-37 ARAT: n.a.	**Strength tasks** gripping task	NIRS	24-channel optical topography system (ETG-4000, Hitachi Medical Co., Tokyo, Japan)	Brain cortical perfusion, HbO concentration	medium / grip device	grip strength dynamometer (GRIP-D)	grip strength	not simultaneous	I-L
**Harrington, 2020(67)**	20 (20/0)	S: 8/12	S: 62 ±7	**Chronic** >6 months	FMA-UE mAS NIHSS MMSE	FMA: mild stroke 52.9 ±9.1, severe stroke 15.9 ±7.9 ARAT: n.a.	**Reach tasks** reaching task, reaction time task	MRI TMS	Phillips CV Intera 1.5T MRI or Siemens 3T Tim Trio MagProX100 with MagOption, MagVenture Inc, Atlanta, GA	lesion mask, ROI, CST injury rMT, transient inhibition	minimal / camera-based	Northern Digital Inc, Optotrak Certus movement capture system, Waterloo, Canada	reaction time, response time, movement time, distance traveled	not simultaneous	O-C
**Harris-Love, 2011(68)**	11 (11/0)	S: 5/5	S: 54.9 ±3.7	**Chronic** > 7 ± 1 years	FMA-UE	FMA: 26.7 ±6 ARAT: n.a.	**Reach tasks** planar reaching	TMS	Magstim 200 stimulator (The Magstim Company Ltd, Wales, UK)	MEP amplitude, SP	minimal / button press / touch screen, sEMG	sEMG, response pad with an adjustable 6-cm diameter circular button	triceps brachii, biceps brachii, movement time, response time	simultaneous	I-L

**Henriques, 2014(69)**	40 (21/19)	S: 4/17, H: 11/8	S: 64.3 ±12.2, H: 57.9 ±11.9	**Chronic** > 6months	Ataxia rating scale	FMA: n.a. ARAT: n.a.	**Reach tasks** custom-built track or railing	not reported	not reported	PICA and SCA stroke	minimal / button press / touch screen	digitizing pen location was recorded on a 21 in Wacom Cintiq 21UX digitizing tablet	movement time, peak velocity	not simultaneous	O-C
**Hensel, 2021(70)**	27 (14/13)	S: 2/11; H: 3/10	S: 65.7 ±12.1; H: 66.2 ±8.0	**Acute** 4.5 ±2.6 days	NIHSS ARAT	FMA: n.a. ARAT: S 51.7 ±6.5; C 57	**aROM tasks** finger tapping	fMRI TMS	3T Magnetom Prisma scanner with 64-channel head coil Magstim Super Rapid2 stimulator (The Magstim Co. Ltd, Whitland, UK)	BOLD contrast rMT, MEP amplitude	minimal / sensor-based	3D ultrasound-based motion analyzer	relative grip strength, movement amplitude, peak velocity	simultaneous	O-C
**Hensel, 2023(71)**	36 (18/18)	S: 5/13, H: 5/13	S: 66.2 ±13; H: 66.5 ±7.2	**Chronic** > 6 months	EHI NIHSS ARAT	FMA: n.a. ARAT: S 55.5 range 45.3-57; C 57	**Grasp tasks** finger tapping rapid pointing grasping	fMRI (rs), TMS	3T Magnetom Prisma scanner equipped with a 64-channel head coil (Siemens AG) Magstim® Super Rapid2 system (The Magstim Co. Ltd.)	BOLD contrast transient inhibition of M1 and iPS	minimal / sensor-based	not reported (fMRI) 3D movement kinematics using high-resolution motion analyser system (Zebris)	no metric reported (fMRI) trajectory error, movement time, number of velocity peaks, peak velocity	simultaneous	O-C
**Hermsdorf, 2020(72)**	49 (24/25)	S: 16/7, H: 15/8	S: 60.6 ±15.6, H:57.2 ±18.9	**Chronic** > 6 months	MMSE, BDI, SSS	FMA: n.a. ARAT: n.a.	**Complex tasks** motor sequence learning task fingers on keyboard	not reported	Not reported	cerebellar stroke, lesion mask, lesion volume	minimal / button press / touch screen	keyboard for 4 finger sequence	performance index, normalized performance index, movement time	not simultaneous	O-C
**Hermsdörfer, 2003(73)**	74 (37/37)	S: 8/11, H: 5/11	S_{LHD} 53 ±13.3; S_{RHD} 52.7 ±9.2	**Early subacute** >1 months	Paresis (no, slight, moderate, severe)	FMA: n.a. ARAT: n.a.	**Reach tasks** planar pointing movements, gesture imitation	not reported	not reported	left vs. right hemisphere stroke	minimal / sensor-based	ultrasonic motion measurement system (CMS 70, Zebris, Isny, Germany)	end-point error, movement time, relative acceleration phase, relative deceleration phases, number of velocity peaks, peak velocity	not simultaneous	O-C
**Hermsdörfer, 2013(74)**	52 (33/19)	S_{LHD} 6/17; S_{RHD} 0/10; H_L 4/6; H_R 1/8	S_{LHD} 57.6 ±10.6, S_{RHD} 57.6 ±10.6; H_L 52.6 ±12.4: H_R 56.9 ±9.9	**Early subacute** >1 months	repetitive movement yes/no	FMA: n.a. ARAT: n.a.	**Reach tasks** planar pointing task	not reported	not reported	lesion volume, lesion mask	minimal / sensor-based	electromagnetic movement registration system (Liberty, Polhemus), video-based scoring of tool-use-categories	hitting angle, movement time, trajectory path length, movement direction, end-point variability, wrist flexion-extension angle, peak velocity	not simultaneous	O-C
**Hirai, 2016(75)**	22 (10/12)	S: 5/5; H: 3/9	S: 43, H: 50	**Acute** <1 week	MI-UE NHPT	FMA: n.a. ARAT: n.a.	**Strength tasks** grip strength	MRI-DTI	Siemens 3T TIM TrioMRI scanner and 12-channel head coil	CST injury	medium / grip device	digital dynamometry	grip strength	not simultaneous	O-C
**Hordacre, 2019(76)**	23 (8/15)	S: 4/4; H: 10/5	S: 66 ±21; H: 53 ±10	**Chronic** 12-36 months	mAS mRS	FMA: n.a. ARAT: n.a.	**Grasp tasks** grip and lift task lifting task	TMS EEG	64-channel dense-array surface EEG cap (Waveguard, ANT Neuro,	MEP amplitude, rMT, TMS-EEG signal	medium / force sensor	customized grip-lift manipulandum with 2 force transducers (MLP-10, Transducer	peak grip force, hold ratio, mean grip strength, correlation maximum	simultaneous	O-C

									Enschede, the Netherlands)<br>Magstim 200 stimulator (Magstim Company, Dyfed, UK)			Technologies, Temecula, CA)			
**Hordacre, 2020(77)**	61 (36/25)	S: 10/26; H: 8/17	S: 64.4 ±11.1; H: 67.3 ±6.7	**Chronic** > 6months	FMA-UE ARAT	FMA: 43.6 ±13.7, 30.3 ±12.5<br>ARAT: 39.0 ±19.4, 18.7 ±19.0	**Strength tasks**<br>grip strength	MRI<br>TMS<br>EEG (rs)	Siemens 3T MAGNETOM Skyra<br>Magstim 200 stimulator, Magstim, Whitland, Dyfed, UK<br>64 channel Waveguard cap (ANT Neuro, Enschede, Netherlands)	FA, LL CST, LL corpus callosum, LL posterior limb internal capsule, MEP presence,<br>power analysis	medium / grip device	hand dynamometer (SH5001 Saehan Hydraulic Hand Dynamometer, Saehan Co, Masan, Korea)	grip strength	not simultaneous	O-L
**Hosomi, 2016(78)**	39 (39/0)	S: 16/23	62.9 ±13.8	**Early subacute** < 8 weeks	FMA-UE, BS, NIHSS, FIM	FMA: S_E 39.5 range 21.3-54; S_C 37 range 13-55<br>ARAT:	**aROM tasks**<br>finger tapping motion<br>grip strength	rTMS	magnetic stimulator (MagPro, Medtronic Functional Diagnostics A/S; or Magstim Rapid, Magstim)	rMT	minimal / sensor-based	magnetic sensors (UB-1; Finger Tapping Movement Analyzer; Hitachi Corporation, Tokyo, Japan), grip strength not reported	grip strength, number of successful trials, total distance traveled, mean amplitude, peak amplitude, mean velocity, peak velocity	not simultaneous	I-L
**Hsu, 2013(79)**	12 (12/0)	S: 4/8	S_E: 56.8 ±6.8; S_C: 62.3 ±8.5	**Early subacute** 2-4 weeks	FMA-UE, ARAT, NIHSS, mRS	FMA: S_E 35.8 ±11.8; S_C 37.0 ±12.4<br>ARAT: S_E 17.6 ±12.5; S_C 14.3 ±7.2	**aROM tasks**<br>finger-lifting each 7 seconds (during MEG)<br>voluntary contraction at 20% MVC (during TMS)	MEG<br>rTMS<br>iTBS	306-channel Elekta Neuromag MEG device (0.05-250 Hz band-pass, digitized at 1 kHz)<br>Magstim Rapid2 simulator (Magstim Co., Whitland, UK)	motor task-dependent cortical oscillations, ERD, ERS,<br>MEP amplitude, aMT	minimal / sEMG	EMG recording system (Synergy EMG/EP system using Ag-AgCl electrode)	extensor carpi radialis, abductor pollicis brevis, % MVC	simultaneous	I-L
**Hummel, 2006(80)**	11 (11/0)	S: 5/6	S: 57 ±16	**Chronic** 41.8 ± 26.4 months	JTHF, FMA-UE, MRC, mAS, MMSE	FMA: 81-99% of 66<br>ARAT: n.a.	**Complex tasks**<br>reaction time<br>maximum pinch force	tDCS	Phoresor® II Auto (Model No. PM850, IOMED®, Salt Lake City, Utah 84120 USA) and two electrodes (TransQE from IOMED®, surface area 25 cm2 per electrode)	FES cortical stimulation	medium / grip device<br>minimal / sEMG	Jamar dynamometer<br>EMG (Dantec Electronics Sklovlunde)	pinch force/strength, flexor carpi radialis, reaction time	not simultaneous	I-L
**Hummel, 2009(81)**	28 (14/14)	S:7/7, H:9/5	S 63.8 ±3, H 63.9 ±2.7	**Chronic** > 12 months	FMA-UE, JTHF,	FMA: n.a.<br>ARAT: n.a.	**Complex tasks**<br>index abduction	TMS	two Magstim 200 magnetic stimulators (Magstim	SAI	minimal / button press /	button press to signal on screen	reaction time	simultaneous	O-C

					mRS, mAS		reaction time task		Company, Whitland, Dyfed, UK).		touch screen				
**Ilić, 2016(82)**	26 (26/0)	S: 9/17	S: 60.0 ±6.4	**Chronic** > 9months	FMA-UE, JTHF	FMA: 48.85 ±6.9 range 36-58	**Strength tasks** Grip strength (secondary)	EEG, tDCS	tDCS with Endomed 482 device 215 (Enraf-Nonius B.V., Rotterdam, Netherlands) with settings ranging from 0.5 to 2mA in 0.2mA increments	lesion location	medium / grip device	hand dynamometer (DyN)	grip strength	not simultaneous	I-L
**Jacobs, 2009(83)**	26 (20/6)	S_A: 7/3, S_{NA}: 4/6, H: not reported	S_A: 51.4, 34-58 ; S_{NA}: 55.8, 45-71, H: 19.7 ±.82	**Early subacute** 26-217 days	Tool-use score	FMA: n.a. ARAT: n.a.	**Reach tasks** reaching/pointing task to short/long stick	MRI/CT	not reported	left hemipshere stroke	minimal / sensor-based	Fastrack System (Polhemus Inc., Colchester, USA)	end-point error, number of velocity peaks, number of submovements, peak velocity	not simultaneous	O-C
**Jang, 2016(84)**	48 (48/0)	S: 17/31	S: 52.9±10.6	**Early subacute** 23 ±15.4 days	MFT, PPT, mBI, EHI	FMA: n.a. ARAT: n.a.	**Strength tasks** grip strength	MRI-DTI	1.5T Philips Gyroscan Intera system with a synergy-L Sensitivity Encoding head coil was used for DTI scanning	FA, ADC, voxel number	medium / grip device	Jamar Hydraulic Hand Dynamometer, model-5030J1	grip strength	not simultaneous	O-C
**Jian, 2021(85)**	44 (11/33)	S: 7/4, H_Y: 15/5, H_O: 7/6	S: 45.7 ±7.6, H_Y: 23.9 ±1.9, H_O: 55.2 ±6.4	**Chronic** 11.1 ± 5.4 months	FMA-UE	FMA: range 22-42 ARAT: n.a.	**Complex tasks** tracking task via elbow flexion/extension	fNIRS	fNIRS braincap with 32 probes (16 sources, 16 detectors) in 36-channel (NirSmart, Danyang Huichuang Medical Equipment Co. Ltd, China)	HbO concentration, HHb concentration	minimal / sEMG	EMG data converter (DAQ-6341, National Instruments, Austin, TX, USA)	biceps brachii, triceps brachii root mean square error, tracking error	simultaneous	I-L
**Jochumsen, 2015(86)**	20 (5/15)	S: 3/2, H: 12/3	S: 51-72, H: 27 ±11	**Early subacute** 35-65 days	ARAT	FMA: n.a. ARAT: range 11-47	**Strength tasks** palmar grasping task, at 20% and 60% MVC and fast vs. slow execution	EEG	9 channels continuous monopolar system (EEG amplifiers, Nuamps Express, Neuroscan)	MRCP	medium / grip device	handgrip dynamometer with 2000Hz sampling rate and coppled with visual cueing	grip strength	simultaneous	O-C
**Johansen-Berg, 2002(87)**	27 (11/16)	S: 2/9, H: not reported	S 50.4 ±11.14; C 40.7 ±14	**Late subacute** 4-40 months	not reported	FMA: n.a. ARAT: n.a.	**Complex tasks** reaction time task (simple = index finger, choice = random out of 4 fingers)	fMRI TMS	3T VarianSiemens MRI system 2T Magstim Rapid Stimulator (Magstim, Camarthenshire, Wales, U.K.)	ROI, rMT	not reported	not reported	reaction time	simultaneous	O-C

**Kang, 2018(88)**	14 (14/0)	S: 10/4	S_{LHD}: 60.9 (52.3-79.8) S_{RHD}: 76.1 (73.0-76.8)	**Chronic** > 6 months	BBT	FMA: n.a. ARAT: n.a.	**Strength tasks** bilateral isometric force control, wrist and finger extension 5,25, 50% MVC	not reported	not reported	left vs. right hemisphere stroke	medium / force sensor	EMG, two force transducers (MLP-75,TransducerTechniques,4.16x1.27 x1.90cm, range = 75lbs, 0.1% sensitivity)	mean force, force/strength asymmetry, force error, force variability (SD)	not simultaneous	I-L
**Kantak, 2018(89)**	20 (10/10)	S: 5/5, H: 7/3	S: 60.4 ±6.80, H: 65 ±7.02	**Chronic** > 6 months	FMA-UE	FMA: 50.1 ±13.82 ARAT: n.a.	**Reach tasks** planar path tracing in kinereach setup	TMS	Magstim 200 stimulator	MEP amplitude, CSE, SP, TCI, RC	minimal / camera-based, sEMG	EMG, electromagnetic tracking system 3D Guidance trakSTAR NDI	triceps brachii, end-point error, end-point error/movement time, movement time, distance traveled, normalized jerk, number of submovements, change in acceleration	not simultaneous	O-C
**Karnath, 2005(90)**	32 (32/0)	S: 10/26	$S_{LHDoptic\ ataxia}$: 52.5 ±11.7, H_L: 65.2 ±11.7 $S_{RHDoptic\ ataxia}$: 64.2 ± 11.8, H_R: 64.8 ±10.5	**Early subacute** 45.1 and 7.1 days	paresis yes/no	FMA: n.a. ARAT: n.a.	**Reach tasks** 3D reaching with foveal and peripheral vision	MRI/CT	not reported	lesion mask	minimal / camera-based	video recordings in two reaching conditions	number of errors	not simultaneous	O-C
**Kemlin, 2019(91)**	40 (40/0)	S: 14/26	S: 63 (33–82)	**Late subacute** 3.5 (1.1–58) months	FMA-UE, JTHF	FMA: 52 range 5–58 ARAT: n.a.	**Grasp tasks** fMRI turning cards, picking up objects, stacking checkers, light and heavy can lifting	TMS fMRI MRI-DWI	MAGSTIM 2002 stimulator fMRI and MRI-DWI not reported	MEP amplitude, rMT, BOLD contrast ROI, FA	medium / grip device	EMG manipulandum	grip strength	simultaneous, not simultaneous, simultaneous	O-C
**Kemppainen, 1999(92)**	16 (16/0)	S: 5/11	S: 53 (31-70)	**Early subacute** 11 days (range 5-18)	Scandinavian Stroke Scale; mRS	FMA: n.a. ARAT: n.a.	**Strength tasks** finger-thumb grip force (compared to bite force)	MRI/CT	CT with a Siemens Somatom HiQ unit and 1T Siemens Magnetom scanner	lesion location	medium / force sensor	quartz force-transducer (type 9001, Kistler Instrument AG, CH 8408 Winterthur, Switzerland)	pinch force/strength	not simultaneous	O-C
**Kim, 2006(93)**	15 (15/0)	S: 2/13	S: 53.5±4.5	**Late subacute** > 3months	PPT	FMA: n.a. ARAT: n.a.	**complex tasks** block of sequential finger motor tasks	TMS	Magstim Rapid stimulator with 2 booster modules (Magstim Co, Ltd)	rMT, MEP amplitude	minimal / button press / touch screen	7-digit sequence with a customized keyboard	grip strength, movement accuracy, movement time	not simultaneous	I-L
**Kim, 2018a(94)**	20 (20/0)	rTMS: 4/4, C: 8/4	rTMS: 51±3, C: 74.1±2.9	**Early subacute** < 3months	BBT	FMA: n.a. ARAT: n.a.	strength tasks grip and pinch	rTMS, high-frequency	MagPro R30 (Medtronic, Skovlunde, Denmark) and DantecTM KeyPointVR (Natus, CA)	MEP amplitude, rMT, MEP latency, overall cortical exciability	medium / pinch gauge	KEYPOINT®.NET and software, JAMAR® Hand Evaluation	pinch force/strength, grip strength	not simultaneous	I-L

**Kim, 2018b(95)**	14 (14/0)	S: 4/10	S_E 52 (49-74), S_C 66 (49-72)	**Late subacute** ≥3 months	MAL	FMA: n.a. ARAT: n.a.	grasp tasks	TMS	MagPro R30 (Medtronic, Skovlunde, Denmark) and DantecTM KeyPointVR (Natus, CA)	MEP amplitudes, MEP latency	minimal / sensor-based	3D motion analysis system (Compact measuring system10, Zebris Medical GmbH, Isny, Germany), Ag/AgCl surface electrodes	movement time, number of submovements, peak angular velocity	not simultaneous	I-L
**Kimberley, 2004(96)**	16 (16/0)	S: 5/11	S 60.1 ±14.5	**Chronic** < 6months	BBT, MAL, JTHF	FMA: n.a. ARAT: n.a.	aROM tasks finger movement control	fMRI	4T whole-body research MRI system (Oxford, UK)	BOLD contrast	minimal / sensor-based	electrogoniometer	finger flexion-extension force/toque, tracking precision	simultaneous	I-L
**Koganemaru, 2010(97)**	18 (9/9)	S: 5/4, H: 5/4	S: 51.6 ±11.6, H: 53.2 ±13.8	**Late subacute** > 5 months	mAS	FMA: n.a. ARAT: n.a.	aROM tasks wrist and finger extension	TMS	Magstim Super Rapid Magnetic Stimulator (Magstim, Whitland, Dyfed, UK)	MEP amplitude, rMT, SP, lesion mask	minimal / sEMG, camera-based	sEMG, video-camera, force transducer	biceps brachii, pinch force/strength, wrist flexion-extension force/torque, finger flexion-extension angle, wrist flexion-extension angle	simultaneous	I-C
**Konczak, 2010(98)**	27 (16/11)	S: 5/11, H: 4/7	S: 60.1 ±14.4, H: 59 ±10.3	**Acute** mean: 14.5 days range: 1-33 days **Subacute** 2 weeks, 3 months	ICARS	FMA: n.a. ARAT: n.a.	reach tasks finger pointing 3D with and without target	MRI	1.5T Siemens Sonata scanner	lesion mask (used in VLSM)	minimal / sensor-based	3D ultrasound-based motion analysis system (Zebris Medizintechnik) at a sampling rate of 100 Hz	movement time, movement velocity, peak acceleration	not simultaneous	O-L
**Koob, 2023(99)**	44 (20/24)	S: 8/12, H: 12/12	S: 71.6 ±10.7, H: 67.0 ±7.8	**Early subacute** 7.7 ± 6.78 days	JTHF	FMA: n.a. ARAT: n.a.	strength tasks grip force task	MRI-DTI	not reported	lesion mask	medium / grip device	strain-gauge based isometric dynamometer with a 0–800 Newton (N) range	grip strength	not simultaneous	I-L
**Kou, 2013(100)**	74 (51/23)	not reported	S: 53.9 ±12.7, H: 50.6 ±14.7	**Late subacute** > 3 months	ARAT, NHPT	FMA: n.a. ARAT: n.a.	strength tasks grip strength	MRI-DTI	scanner not reported, automated lesion identification toolbox	lesion mask, CST injury	medium / grip device	Jamar Hand Dynamometer	grip strength	not simultaneous	O-C
**Küper, 2011(101)**	20 (20/0)	S: 4/16	S: 57	**Chronic** > 6months	ICARS	FMA: n.a. ARAT: n.a.	grasp tasks reach-to-grasp analysis, grip force	MRI	Siemens Espree 1.5T MR scanner	lesion mask (used in VLSM)	minimal / camera-based, sensor-based	custom-made instrumented objects, ultrasound-based motion-measurement system (CMS-HS, Zebris, Isny, Germany) with 3 markers	grip strength, number of successful trials, movement time, path length ratio, velocity at start, grip aperture, grip aperture, peak grip aperture, peak velocity	not simultaneous	O-C
**Kuppuswamy, 2015(102)**	70 (70/0)	S: 20/50	S: 60.36 ±12.4	**Chronic** 56.81 ±63 months	NHPT, ARAT	FMA: n.a. ARAT : RHD_{male} 97.6 ± 9.1; LHD_{male} 98.1 ± 3.5; RHD_{female}	aROM tasks voluntary activation affected elbow flexor group of muscles	TMS	BiStim magnetic stimulator (Magstim)	rMT, aMT, SICI, LICI	medium / grip device, minimal / sEMG	dynamometer, electrodes (1041PTS Neonatal Electrode, Kendell)	biceps brachii, grip strength	simultaneous	O-C

						99.5 ± 1.2; LHD$_{female}$ 98.4 ± 4.5									
**Kwon, 2007(103)**	31 (20/11)	S: 4/16, H: 2/9	S1: 60.1 ±3.1, S2: 57.6 ±2.6, H: 33.0 ±2.3	**Chronic** > 6months	MRC, PPT	FMA: n.a. ARAT. n.a.	**Grasp tasks** grasp-release hand movements, grip strength	fMRI	1.5T MR scanner (Vision, Siemens, Germany)	BOLD contrast, laterality index, ROI	medium / grip device	not reported, metronom-guided hand dynamometer (Baseline, USA)	no metric reported for fMRI grasp activity grip strength	simultaneous	O-C
**Kwon, 2014(104)**	14 (14/0)	S: 3/11	S 53.3±12.4	**Chronic** 22.5 ± 24.5 months	NHPT, PPT	FMA: n.a. ARAT. n.a.	**complex tasks** sequential finger tapping task on keyboard	rTMS (combined with training)	Rapid2 ® stimulator TMS system (Magstim, UK)	MEP amplitude, rMT	minimal / button press / touch screen	5 finger button push sequence shown on 15 inch monitor, Synergy Electromyography (EMG)/EPsystem (Medelec, UK),	movement accuracy, movement time	simultaneous	I-C
**Lafe, 2023(105)**	31 (17/14)	S: 7/10, H: 8/6	S: 47-74, H: not reported	**Chronic** 38-260 months	not reported	FMA: n.a. ARAT. n.a.	complex tasks pinch precision grip force tracing task	MRI	Prisma 3T scanner (Siemens, Erlangen, Germany) with 64-channel head / neck receiver radiofrequency (RF) coil	CST injury	medium / force sensor	precision force signals sampled at 200 Hz using a 6-axis force sensor (Mini40, ATI Industrial Automation)	pinch force/strength, precision force	not simultaneous	O-C
**Lai, 2015(106)**	72 (72/0)	S_1 4/17, S_2 3/14, S_3 4/13, S_4 4/13	S_1: 62.6 ±11.6 S_2: 60.4 ±10.4 S_3: 63.4 ±12.1 S_4: 62.1 ±10.5	**Chronic** S_1: 10.4 ±6 S_2: 9.7 ±5 S_3: 11.4 ±4 S_4: 10.6 ±5 months	MRC, WMFT	FMA: n.a. ARAT: n.a.	**aROM tasks** finger tapping task simple reaction time task	rTMS iTBS	Magstim Rapid2,	MEP amplitude, rMT	not available / not reported	not reported	grip strength	not simultaneous	O-L
**Lang, 2005(107)**	49 (39/10)	S: 21/18, H: 5/5	S: 65.0 ±13.4 H: 59.1 ±12.5	**Acute** 9.6 ±4.5 days	ARAT, FIM	FMA: n.a. ARAT: 21.5 ±14.7	grasp tasks Reach-to-grasp movements	MRI/CT	not reported	CST injury, lesion volume	minimal / camera-based	KinTrak software (Motion Analysis Corporation)	end-point error, peak aperture rate, movement time, time to peak grip aperture, wrist position, grip aperture, wrist angular velocity	not simultaneous	O-C
**Larivière, 2018(108)**	41 (17/24)	S: 10/14, H: 3/14	46.7 ± 17.5 / 53.2 ± 12.3	**Chronic** 44.9 ±56.6 months	BBT, NHPT	FMA: n.a. ARAT: n.a.	strength tasks isometric, visually paced hand grips	fMRI, DTI	not reported	functional connectivity (FC)	medium / grip device	MR-compatible manipulandum	grip strength	simultaneous	O-C
**Larsen, 2017(109)**	37 (19/18)	S: 2/17, H: not reported	S: 61 (31-86), H: not reported	**Acute** 1-8 days	Grooved pegboard test	FMA: n.a. ARAT: n.a.	**Strength tasks** grip task, dynamic pinch task	EEG	64 active electrodes (ActiveTwo, BioSemi, Amsterdam, The Netherlands)	CMC, IMC	medium / force sensor	load cell (UU2-K30, Dacell, Korea)	grip strength	simultaneous	O-L
**Larsen, 2018(110)**	33 (15/18)	S: 2/13; H: 5/13	S: 59 (31-86); H: 65(32-88)	**Acute** 1-8 days	Grooved pegboard test, MRC	FMA: n.a. ARAT: n.a.	**Strength tasks** pinch grip task, precision grip task	EEG	64 active electrodes (ActiveTwo, BioSemi,Amsterdam, NL) on cap (Headcap Bio-Semi, NL) 10-10	power analysis	medium / pinch gauge	pinch force with a load cell (UU2-K30, Dacell, Korea)	grip strength	simultaneous	O-L

									system using ActiView (version 6.05)						
**Lefebvre 2015(111)**	19 (19/0)	not reported	not reported	**Chronic** >6months	NIHSS, ABILHAND	FMA: n.a. ARAT: n.a.	**Complex tasks** moving a pointer along a complex circuit as quickly and accurately as possible	fMRI	3T scanner attached to a 32-channel head coil (Siemens Verio)	BOLD contrast	minimal / button press / touch screen, medium / grip device	MRI-compatible mouse, whole-hand Jamar dynamometer	end-point error, normalized jerk, mean velocity	simultaneous	I-L
**Lewis, 2007(112)**	24 (15/9)	S: 4/11, H: not reported	S: 58 ±14, H: 55 ±12	**Chronic** > 12 months	FMA-UE	FMA: 42 ±12 ARAT: n.a.	**aROM tasks** wrist pronation-supination unimanual, bimanual, phase and anti-phase	TMS	Magstim 200 (Magstim Company, Wales)	MEP amplitude, rMT	minimal / camera-based	Wrist support splint with four light-emitting diode (LED) markers Optotrak 3020 system (Northern Digital Incorporated, Waterloo, ON) sampled at 200 Hz.	movement frequency, coefficient of variation, in-phase synchronicity, anti-phase synchronicity, movement amplitude	simultaneous	O-C
**Liepert, 2005(113)**	16 (8/8)	S: 3/5, H: 3/5	S: 60.4 ±13, H: 59.2 ±11.6	**Early subacute** <3weeks	NHPT	FMA: n.a. ARAT: n.a.	**Strength tasks** grip strength	TMS	Magnetic stimulator (Magstim 200 HP), The Magstim Comp., Dyfed, UK	thalamus stroke, MEP latency, rMT, CMCT	medium / grip device	mechanical dynamometer	grip strength	not simultaneous	O-C
**Liepert, 2007(114)**	12 (12/0)	S: 4/7	S: 63	**Acute** 7.3 ± 4.5 days	NHPT	FMA: n.a. ARAT: n.a.	**Strength tasks** grip strength	TMS	MagLite-r25 stimulator (Medtronic Company, USA)	rMT	medium / grip device	dynamometer	grip strength	not simultaneous	I-C
**Lindberg, 2007(115)**	16 (7/9)	S: 2/5; H: 5/4	S: 55.4 (40-71), H: 50.8 (38-65)	**Chronic** > 6 months	BBT, NHPT, mAS	FMA: n.a. ARAT: n.a.	**Strength tasks** grip strength	MRI-DTI TMS	1.5T General Electric Signa Echospeed (Milwaukee, WI) MagPro R30 (Medtronic, serial number 108, Minneapolis, MN)	FA MEP	medium / grip device	Grippit	first dorsal interosseous, grip strength wrist-extension	not simultaneous	O-C
**Lindberg, 2009(116)**	23 (11/12)	S 2/9; C 6/6	S 59 (range 37-73); C 51 (range 32-68)	**Chronic** 20-115 months	mAS	FMA: n.a. ARAT: n.a.	**aROM tasks** wrist flex/ext slow and fast passive movements	fMRI	1.5T General Electric Signa Echospeed scanner (Milwaukee, WI)	BOLD contrast	medium / grip device minimal / sEMG	MRI compatible resistance trajectory during slow and fast movements, grip strength (Grippit instrument, AB Dektektor, Göteborg, Sweden), EMG	flexor carpi radialis, force trajectories, grip strength	simultaneous	O-C

**Liu, 2017(117)**	32 (32/0)	S: 6/26	S: 60.5 (7.8, 46–75)	**Chronic** >1 year post-stroke	FMA-UE, PPT	FMA: 58.4 ±8.9 range 29–66 ARAT: n.a.	**Strength tasks** pinch force	TMS	Magstim Company Ltd, Whitland, Dyfed, United Kingdom	rMT, RC slope, MEP amplitude, SP	medium / pinch gauge	Jamar Digital pinch gauge (Sammons Preston, Roylon, Bolingbrook, IL)	pinch force/strength	not simultaneous	I-L
**Lopez-Larraz, 2018(118)**	20 (20/0)	S: 7/13	S: 48.5 ± 14.5	**Chronic** 54.9 ±61.0 months	modified FMA-UE	FMA: 12.8 ±8.4 ARAT: n.a.	**aROM tasks** movement attempt to open and close hand	EEG	16-electrodes Acticap system (BrainProducts GmbH, Germany)	alpha band frequency, beta band frequency, EEG-EEG coherence, CMC, IMC	minimal / sEMG	bipolar Ag/AgCl electrodes (Myotronics-Noromed, USA)	extensor carpi ulnaris, extensor digitorum, biceps brachii, triceps brachii	simultaneous	O-C
**Lotze, 2006(119)**	14 (7/7)	S: 1/6, H: 5/2	S: 63.7 ± 8.6, H: 63.9 ± 4.5	**Chronic** < 8 months	n.a.	FMA: n.a. ARAT: n.a.	**aROM tasks** sequential finger tapping and hand tapping with recovered hand	fMRI, TMS	3T (Trio, 8 high-frequency head coil; Siemens, Erlangen, Germany) Magstim rapid stimulator (Magstim, Whitland, UK)	ROI, rMT	minimal / button press / touch screen	sequential finger-tapping task on MR-compatible keyboard to track movement	timing error, number of errors	simultaneous	I-C
**Lotze, 2012(120)**	38 (14/24)	S: 4/14; H: 14/10	S: 58.0 ± 14.4; H: 59.5 ± 16.0	**Late subacute** > 5months	MRC, NHPT, NIHSS	FMA: n.a. ARAT: n.a.	**Strength tasks** pressing a rubber ball, performance of maximal frequent voluntary	fMRI TMS DTI	1.5T MRI scanner (Siemens Symphony) Magstim, Whitland, Dyfed, UK 3T MRI scanner (Siemens Verio, Erlangen, Germany)	BOLD contrast FC FA	minimal / force sensor	pressure detector connected to an electro-optical biosignal recorder (Varioport-b, Becker Meditec, Karlsruhe, Germany). passive wrist flexion–extension (Neurobehavioral Systems, Albany, NY)	% MVC, peak velocity	simultaneous not simultaneous	O-C
**Lu, 2020(121)**	26 (26/0)	S: 3/23	S: 49±15.4	**Chronic** > 6 months	BI	FMA: n.a. ARAT: n.a.	**aROM tasks** wrist ROM task	EEG	24-Channel high-resolution EEG system (NGERP-P, NCC Medical Co., LTD, Shanghai, China)	ERD, ERS, power spectrum density	not available / not reported	not reported	wrist flexion-extension angle	not simultaneous	I-L
**Luft, 2004(122)**	21 (21/0)	S_E: 2/7, S_C: 7/5	S_E 63.3 ± 15.3/ S_C 59.6 ± 10.5	**Late subacute** >3 months	FMA-UE, WMFT	FMA: E 29.6 ± 3.5; C 28.3 ± 2.1 ARAT: n.a.	**aROM tasks** elbow flexion and extension	fMRI	not reported	BOLD contrast	minimal / camera-based, sEMG	Video monitoring with 2 cameras (one focused on the head, the other on the elbows), EMG on biceps and deltoid	biceps brachii, deltoid, elbow flexion-extension force/torque, shoulder flexion-extension force/torque	simultaneous	I-L
**Mani, 2013(123)**	38 (18/20)	S 8/10, H 4/16	S_L 68.6 ±11.3, S_R 63.2 ±10.6, H_L 63.6 ±6.3, H_R 64.2 ±9.5	**Chronic** >6 months	FMA-UE	FMA: S_L 57.3 ±6.3; S_R 57.3 ±6.5 ARAT: n.a.	**Reach tasks** target reaching task	MRI	not reported	lesion volume, lesion location	medium / end-effector	Kinreach 2D, Air sled system, Flock of birds	trajectory error, end-point error, absolute end-point direction error, movement time, initial movement direction error, peak velocity	not simultaneous	O-C
**Mansur, 2005(124)**	16 (10/6)	S: 7/3, H:3/3	S: 53.3 (range 37-73), H: 43.6	**Chronic** 6-12months	PPT	FMA: n.a. ARAT: n.a.	**aROM tasks** finger tapping test, simple reaction time	rTMS	Magstim Super Rapid Stimulator	lesion location	not available / not reported	not reported	finger tapping, reaction time	simultaneous	I-C

			(range 28-52)				task and choice reaction time task							
**Matarasso, 2021(125)**	14 (4/10)	S: 3/1, H: 4/6	S 46-67, H 43.8 ± 22.9	**Chronic** > 6 months	FMA-UE, AMAT	19.7 ± 1.7	**aROM tasks** wrist extension	fMRI fNIRS	3T Phillips Achieva scanner ETG-400 NIRS system (Hitachi Medical Systems, Japan)	ROI HbO concentration	not available / not reported	not reported	wrist flexion-extension angle	simultaneous	I-L
**Matsuura, 2015(126)**	20 (20/0)	S: 11/9	S_E 72.2 ±6.0, S_C 74.7 ±12.7	**Early subacute** 4-21 days	FMA-UE, PPT	50.0 ± 10.7, 50.8 ± 11.6	**aROM tasks** self-paced extension of the affected wrist	EEG TMS	6 channel EEG system Magstim Rapid stimulator (Magstim Co., Whitland, UK)	MRCP, RP, negative slope, rMT	minimal / sEMG, grip device	EMG, digital hand dynamometer	extensor carpi radialis, grip strength	simultaneous	I-C
**McKenzie, 2017(127)**	40 (40/0)	S: 11/29	S: 58 ±14	**Late subacute** 3-6 months	FMA-UE, ARAT, BBT, BI, SIS hand	FMA: 35.6 ±14.4 ARAT: 25.1 ±18.7	**Complex tasks** wrist flex-ext and finger movements, reaction time task, Robot based BBT	MRI	Achieva 3T TX MRI System	CST injury, injury of hand region of M1	high / robot-based	Hand Wrist Assistive Rehabilitation Device robot	end-point error, reaction time, peak velocity	not simultaneous	O-C
**McPherson, 2018(128)**	18 (10/8)	S: 3/7 H: 1/7	S: 60 ±9	**Chronic** 2-21 years	FMA-UE	23 ±8	**Reach tasks** 3D pointing (ACT3D table, floating, 25% arm load)	EEG	HD-EEG system with 160 active Ag/AgCl electrodes (Active II, Biosemi, Inc., Amsterdam, The Netherlands)	RP, laterality index	high / robot-based, sEMG	ACT-3D Robotic System, EMG, 3D magnetic digitizer (Polhemus, Colchester, VT, USA)	brachioradialis, biceps brachii, triceps brachii, deltoid, pectoralis major, latissimus dorsi, flexor carpi radialis, extensor carpi radialis, angular excursion, angular velocity	simultaneous	O-C
**Miller, 2019(129)**	13 (13/0)	S: 4/9	S: 65.9 ±8.7	**Chronic** 70.3 ± 48.1 months	FMA-UE, WMFT	28.8 ±19/66	**Strength tasks** gripping task	rTMS	Magstim Super Rapid[2] stimulator combined with BrainsightTM neuronavigation software pack B	CSE, TCI, MEP amplitude	medium / grip device	hand-held dynamometer	extensor carpi radialis, extensor carpi ulnaris, extensor digitorum communis, % maximum voluntary contraction (MVC)	not simultaneous	I-L
**Mutha, 2014(130)**	45 (27/18)	S_{LHD}: 6/8, S_{RHD}: 7/6	S_{LHD}: 60.9 ±1.1, S_{RHD}: 71 ±2.39	**Chronic** >6 months	not reported	not reported	**reach tasks** planar reaching task	MRI	not reported	lesion location	medium / end-effector	Kinereach 2D, Air sled system, Flock of birds	end-point error, movement time, peak velocity	not simultaneous	O-C
**Naros, 2017(131)**	20 (20/0)	S_{ctACS}: 2/8, $S_{i\text{-}tACS}$: 4/6	$S_{c\text{-}tACS}$: 56.6 ± 12.3, $S_{i\text{-}tACS}$: 55.6 ± 9.8	**Chronic** > 6 months	FMA-UE	35 ±18 and 52 ±13	**aROM tasks** rs, finger flex-ext, ERD-triggerted	EEG, tACS	32 Ag/AgCl electrodes, BCI2000 software platform	power analysis, beta band frequency, ERD, power variance	Group C / robot-based	hand robot (Amadeo®, Tyromotion, Graz, Austria) for finger flex-ext	extensor digitorum communis, digitorum superficialis	simultaneous	I-C
**Newton, 2006(132)**	15 (3/12)	S: 1/2, H: 4/8	S: 64.3 ±10.0, H: 48.5	**Chronic** >20 months	BI, MI, NHPT	Barthel : 20, MI : 86.6 ± 26.5, NHPT : 64.3 ± 10.0	**Strength tasks** dynamic isometric hand-grip task	fMRI	3T Siemens Allegra MRI System (Siemens, Erlangen, Germany)	BOLD contrast, FA	medium / grip device	MRIcompatible manipulandum	grip strength, % maximum voluntary contraction (MVC)	simultaneous	O-C

**Nicolo, 2018(133)**	41 (41/0)	S: 18/23	S_{cTBS} 62.4 ±12.3, S_{tDCS} 68.5 ±10.8, S_{Sham} 64.3 ±17.1	**Early subacute** 2-10 weeks	FMA-UE, BBT, NHPT	cTBS: 12.9 ±11.7, tDCS: 13.3 ±10.5 Sham:15.2 ±14.4	**Strength tasks** grip strength	rTMS-cTBS tDCS EEG	MagPro X100 stimulator coil (MCF-B65) or sham coil (MCF-P-B65), tDCS with two 35-cm2 electrodes, 128-channel Biosemi ActiveTwo EEG system	effective connectivity, partial directed coherence (PDC), FC, weighted node degree, EEG-EEG coherence, alpha band frequency, beta band frequency	medium / grip device	Jamar dynamometer	grip strength	not simultaneous	I-L
**Noh, 2019(134)**	22 (22/0)	S: 12/10	S: 65.2	**Chronic** S_E: 31.4 ± 16.9 days S_C: 22.6 ± 7 days	FMA-UE, BS, MFT	FMA: S_E: 28.5±21.2 (27, 40), S_C: 20.6±18.5 (13, 28) ARAT: n.a.	**Strength tasks** grip strength	rTMS with and without action observation therapy	Magstim Super Rapid stimulator (Magstim Co., Whitland, Dyfed, UK)	MEP amplitude, MEP latency, rMT	medium / grip device	dynamometer	grip strength	not simultaneous	I-L
**Nowak, 2008(135)**	15 (15/0)	S: 4/11	S: 46 ± 8	**Early subacute** 1-4months	ARAT, NIHSS, mRS	FMA: n.a. ARAT 45.2 ±4.7	**Grasp tasks** hand grip movements at 1.5 Hz, finger tapping, reach-to-grasp	fMRI rTMS	Trio 3T, Siemens, Erlangen, Germany Magstim Rapid stimulator (Magstim Company, Dyfed, Wales)	Boxcar vectors (neural activity), rMT	minimal / observer counts, camera-based	observer counted, documents and video taping	number of successful trials, movement time, wrist position, wrist angular velocity, movement velocity	simultaneous	I-L
**O'Shea, 2014(136)**	26 (13/13)	S: 3/10, H: 8/5;	S: 66 (30-80); H: 24.6 +/- 4.8	**Chronic** > 6 months	FMA-UE, WTMF	FMA: 43.2 ±17 (16-66) ARAT: n.a.	**Complex tasks** Reaction time task with paretic hand after tDCS	TMS, tCDS, MRS (magnet resonance spectroscopy)	monophasic stimulators (Magstim Company, Whitland, Dyfed, U.K.), tDCS (Magstim, Whitland, Dyfed, U.K.) 1mA, 20 min via 2 rubber electrodes	MEP amplitude, MEP duration	minimal / button press / touch screen	joystick wrist flexion response	reaction time	not simultaneous	I-L
**Ouin, 2022(137)**	1180 (394/786)	S: 151/243	S: 63.7 ±10.6	**Chronic** 6 months	NIHSS	FMA: n.a. ARAT: n.a.	**aROM tasks** finger tapping task, simple reaction time task	MRI	3T HDXt scanner (General Electric Medical Systems) with an eight-channel head coil	stroke type, stroke number, lesion volume, lesion mask (used in VLSM)	minimal / button press / touch screen	a laptop with a 15” screen, a micro-switch, and Vision 180 software (Godefroy et al., 2010b)	movement time, reaction time	not simultaneous	O-C
**Palmer, 2019(138)**	33 (19/14)	S: 8/11, H: 6/8	S: 66 ±11, H: 53 ±14	**Chronic** > 6 months	FMA-UE, WMFT, NHPT	FMA: 50.4 ±16.1 (14-66) ARAT: n.a.	**Strength tasks** gripping task	TMS EEG	TMS pulses (Magstim 2002, MagStim, Wales, UK), 32-channel electrode cap (Easy Cap, Brain Products GmbH, Gilching, Germany)	rMT, aMT, SP EEG-EEG coherence	medium / grip device	dynamometer	grip strength	not simultaneous	O-C

**Pan, 2018(139)**	12 (12/0)	S: 1/11	56.5 ± 9.5	**Chronic** ES: 38.5 ±25.8, SC: 37.8 ±15 months	FMA-UE	FMA: ES: 49 ±2, SC:47 ±2 ARAT: n.a.	**strength tasks** thumb isometric flexion at 50% MVC for 20 seconds	EEG	16-channel EEG actiCAP and BrainVision V-Amp (Brain Products GmbH, Germany)	CMC	medium / force sensor, minimal / sEMG	force transducer (FT10, Grass Technologies, RI, USA), EMG surface electrodes (11-mm Ag-AgCl, 3.0 cm inter electrode distance) both amplified in real time	force accuracy, force steadiness, thenar eminence muscle	simultaneous	I-L
**Park, 2015(140)**	11 (11/0)	S: 8/3	55.5±5.6	**Chronic** > 6 months	FMA-UE	FMA: 48.1 ±10.1 ARAT: n.a.	**aROM tasks** grasping and supination movement	EEG	64-channel active electrode EEG system (sampling rate: 2048 Hz; Active-two, Biosemi, Amsterdam, The Netherlands)	ERD, ERS, mu band frequency, beta band frequency	high / robot-based	robotic device with grasping handle	grip strength, forearm pronation-supination angle	simultaneous	O-C
**Pennati, 2020(141)**	103 (80/23)	S: 23/57, H: 22/11	S: 52.7 ±9.4, H: 46.9 ±13.1	**Early subacute** 2-6 weeks	FMA-UE, NIHSS, BBT	23.53 ±23.09	**strength tasks** precision grip force control task	MRI	Ingenia 3T MR system (www.usa.philips.com) with an 8HR head coil	lesion mask, LL CST	medium / grip device	digital dynamometer, dynamic index finger and thumb with 2 force sensors	force correlation, force correlation, thumb-index-dexterity score, repeatability score	not simultaneous	O-L
**Pierella, 2020(142)**	12 (6/6)	S 4/2; H 4/2	S 68 ±18; H 58 ±16	**Early subacute** 2-6 weeks	FMA-UE	range 5-54	reach tasks 3D pointing	EEG	64 pre-amplified (active) channel Active II EEG system (Biosemi, Amsterdam)	mean spectral power, coefficient of variation per frequency band	high / robot-based, minimal / sEMG	exoskeleton for the upper limb (Arm Light Exoskeleton Rehab Station, ALEx RS) from Wearable Robotics srl, Ag-AgCl electrodes (Kendall H124SG, ECG electrodes 30 × 24 mm)	trapezius, deltoid, pectoralis major, latissimus dorsi, infraspinatus, rhomboid major, biceps brachii, brachioradialis, triceps brachii, pronator muscles, movement accuracy, mean trajectory error, absolute trajectory error, non-negative matrix factorization of muscles, movement time, number of velocity peaks, spectral arc length (SPARC), mean velocity	simultaneous	O-L
**Piscitelli, 2020(143)**	44 (27/17)	S: 5/22	S: 58.7 ± 10.1, H: 59.8 ± 12.2	**Early subacute** > 1 month	FMA-UE	Group A TMS FM: 55.3 ± 11.3, Group B no TMS: 35.2 ± 14.2	aROM tasks elbow flexion and extension	TMS	Magstim 200 system (Magstim Company Ltd, Whitland, UK)	MEP amplitude, MEP latency	minimal / sEMG, sensor-based	sEMG,electro-goniometer (Novotechnik, USA)	biceps brachii, elbow flexion-extension angle, elbow angular velocity	simultaneous	O-C
**Pohl, 2000(144)**	30 (20/10)	S_{RHD}: 6/4; S_{LHD}: 6/4; H: 7/3	S_{RHD} 71.7 ±13.7, S_{LHD} 67.2 ±13.3, H: 68.7 ±8.1	**Late subacute** >5months	FMA-UE	S_{RHD} 59.4 ±4.9, S_{LHD} 62.0 ±5.1	reach tasks planar reaching with different difficulty levels	not reported	not reported	left vs. right hemisphere stroke	minimal / camera-based	custom-made tapping board and Optotrack for motion analysis	movement time, reaction time, acceleration duration, deceleration duration	not simultaneous	O-C
**Pohl, 2003(145)**	61 (31/30)	S_{RHD} 9/6, S_{LHD} 6/10, H:	S_{RHD} 72.7 ±6.8, S_{LHD} 69.9 ±7.8,	**Chronic** > 6 months	not reported	not reported	reach tasks planar reaching	not reported	not reported	left vs. right hemisphere stroke	minimal / button press /	instrumented board with 3x3 grids and keys	number of errors, reaction time, movement time	not simultaneous	O-C

		not reported	H: not reported								touch screen				
**Quandt, 2019(146)**	17 (17/0)	S:7/10	S: 63.9 ±8.3	**Chronic** > 6months	FMA-UE	FMA: 60.6 ±5.8 ARAT: n.a.	**Grasp tasks** repetitive reaching and grasping movements (pinch grip or whole hand grip)	MRI-DTI EEG	3T Skyra scanner (Siemens, Erlangen, Germany), 63-channel EEG system (actiCAP®, Brain Products GmbH, Gilching, Germany; Electro-Cap International, Inc., Eaton, OH, USA)	ROI, FA power analysis, time-frequency responses	medium / grip device minimal / sensor-based	Strength JAMAR hand evaluation kit ultrasound localization system (CMS20, Zebris, Isny, Germany),	grip strength, pinch force/strength, force ratio, composite motor score	not simultaneous simultaneous	O-C
**Ramos-Murguialday, 2013(147)**	32 (32/0)	S_{BCI} 7/9; S_C 5/9	S_{BCI} 49.3 ±12.5; S_C 50.3 ±12.2	**Chronic** > 10 months	cFMA (/54)	FMA: S_{BCI} 11.15 ±6.92; S_C 13.28 ±10.71	**aROM tasks** hand closing and opening	BCI-EEG fMRI	not reported	laterality index, mu rhythm / SMR	minimal / sEMG	EMG extensor digitorum	extensor digitorum	simultaneous	I-L
**Rehme, 2011a(148)**	22 (11/11)	S: 4/7, H: not reported	S: 65.18 ±9.62, H: not reported	**Acute** <72hrs	ARAT, NIHSS	FMA: n.a. ARAT: 35 ±22	**aROM tasks** fist closure at 1Hz (fMRI) grip strength (fMRI)	fMRI	Siemens Trio 3T scanner (Siemens Medical Solutions, Erlangen, Germany)	BOLD contrast	medium / grip device	observer counted number of fist closures, vigorimeter	flexor carpi radialis, force trajectories, number of successful trials, grip strength	simultaneous	O-C
**Rehme, 2011b(149)**	24 (12/12)	S: 4/8, H: not reported	S: 67 ±9, H: not reported	**Acute** ≤ 72 hours	ARAT	FMA: n.a. ARAT: 41 ±21	**aROM tasks** fist closure at 1Hz (fMRI) grip strength (fMRI)	fMRI	Siemens Trio 3T scanner (Siemens Medical Solutions, Erlangen, Germany)	cortical excitability / activation	minimal / observer counts	observer counted fist closures	composite motor score, number of successful trials	simultaneous	O-C
**Rehme, 2015(150)**	21 (21/0)	S: 5/16	S: 67 ±11	**Acute** 6±3 days	ARAT	FMA: n.a. ARAT: 31 ±22	**aROM tasks** fist closure at 1Hz (fMRI) grip strength (fMRI)	fMRI	3T scanner (Siemens Medical Solutions, Erlangen, Germany)	BOLD contrast	medium / grip device	not reported for fMRI (fist closure) vigorimeter	no metric reported for fMRI grasp activity grip strength	simultaneous	O-L
**Remsik, 2019(151)**	21 (21/0)	S: 12/9	S: 61.6 ±15	**Early subacute** range 44 -2767 days	ARAT, NHPT	FMA: n.a. ARAT: 26.6 ±9	**aROM tasks** attempted and imagined movement trials of cursor task	BCI-EEG	16-channel EEG cap and amplifier (Guger Technologies)	mu rhythm / SMR, mu band frequency	medium / grip device	dynamometer (Nam et al., 2011)	grip strength, hand muscle contraction, elbow muscle contraction	simultaneous	I-L
**Remsik, 2021(152)**	16 (16/0)	S: 8/8	S: 62.5 ±12.7	**Late subacute** >94 days	not reported	FMA: n.a. ARAT: n.a.	**Grasp tasks** grasping movements vs. rest	BCI-EEG	16-channel EEG cap and amplifier (Guger Technologies) visual display (Wilson et al., 2009) and FES adjuvant.	mu rhythm desynchronization, isolated effective coherence	medium / grip device	dynamometer (Boissy et al., 1999)	grip strength	not simultaneous	O-C

**Renner, 2005(153)**	27 (16/11)	S:8/8; H:5/6	S:62 (43-75), H: 30 (20-40)	**Early subacute** 3-13 weeks	RMA	FMA: n.a. ARAT: n.a.	**Strength tasks** pinch grip task at 5%, 10%, 50%, and100% MVC	TMS	Magstim 200 magnetic stimulator (Magstim Company Ltd., UK)	MEP amplitude, peak to peak amplitudes, rMT, aMT	medium / grip device, sEMG	Ag/AgCl electrodes (Medtronic L0202, Denmark)	first dorsal interosseous, grip strength	simultaneous	O-C
**Riga, 2022(154)**	55 (20/35)	S: 7/13, H: 21/14	S 64.5 ±13.9, H: 58.6 ±8.9	**Acute** 2.4 ±1 days	FMA-UE, BBT	FMA: 59 ±6.7	**Reach tasks** REAplan task course navigation in CIRCUIT and REACHING along target line	MRI	not reported	lesion mask (used in VLSM)	high / robot-based	REAplan neurorehabilitation robot (Axinesis, Wavre, Belgium)	spectral arc length (SPARC), peak velocity, grip strength	not simultaneous	I-L
**Rimmele, 2018(155)**	46 (20/26)	S: 11/9	S: 65.0±2.4	**Chronic** 370±6.9 days	NHPT	FMA: n.a. ARAT: n.a.	**Strength tasks** grip strength	MRI-DWI	3T Siemens Skyra magnetic resonance imaging scanner (Siemens, Erlangen, Germany)	CST injury, nigropallidal tract injury, FA	medium / grip device	dynamometer	grip strength	not simultaneous	O-C
**Rondina, 2017(156)**	30 (30/0)	S: 12/18	S: 55.4 ±10.05	**Chronic** > 6months	ARAT, MI, NHPT	ARAT: S_1 32.9 ±8.5, S_2 52.7 ±5.49	**Strength tasks** grip strength	MRI	3T Allegra scanner (Siemens AG, Erlangen, Germany)	LL CST, LL motor ROIs	not available / not reported	not reported	grip strength	not simultaneous	O-L
**Rosso, 2013(157)**	50 (22/28)	S_1: 8/5, S_2: 14/8, H: 14/5	S_1: 44 (35-53), S_2: 57 (45-64), H: 53 (42-64)	**Early subacute** at 3 weeks	NIHSS	FMA: n.a. ARAT: n.a.	**aROM tasks** self-paced whole-hand grip task	fMRI	3T Siemens Trio MR Scanner and a 12-channel head matrix coil	FA, FC	medium / force sensor	mri compatible pressure transducer for grip recognition	grip strength ratio, movement frequency	simultaneous	O-L
**Rosso, 2022(158)**	27 (27/0)	S: 6/21	S_1: 63 ± 14, S_2: 60 ± 11	**Early subacute** >30 days	FMA-UE, JTHF	FMA: S_1 43 ±14, S_2 38 ±19 ARAT: n.a.	**Complex tasks** visually cued, power grip ramp-and-hold force-tracking (visuomotor task)	fMRI, diffMRI, TMS	3T (Magnetom VERIO, Siemens, Erlangen, Germany) with 32-channel coil MAGSTIM 200^2 stimulator units (Magstim, Dyfed, UK)	BOLD contrast, FA, MEP ratio, rMT, MEP amplitude	not available / not reported	grip force device not reported	grip strength	simultaneous	I-L
**Rungsee thanakul, 2022(159)**	18 (18/0)	S_E: 4/5, S_C: 4/5	S_E: 57.9+9.3, S_C: 59.9+8.1	**Early subacute** 1-3 months	FMA-UE	FMA: SE: 51.7+3.1, SC: 49.4+4.3 ARAT: n.a.	**Grasp tasks** reach to grasp cylindrical object with and without barrier condition	TMS	single-pulse TMS (Magstim 200, Magstim Co., Dyfed, UK)	rMT, MEP presence	minimal / sensor-based	electromagnetic tracking motion system (Motion Monitor, Innsport, Inc.), with 6DOF Mini-Bird sensors (Ascension technologies, Chicago, IL, USA) on wrist, thumb and index	movement time, distance traveled, time to peak velocity, relative time to peak grip aperture, peak grip aperture, peak velocity, deceleration duration, movement velocity	not simultaneous	I-L

**Sakurada, 2017(160)**	56 (28/28)	S: 10 /18 H: 10 /18	S: 64.9 ±10.6 H: 65.1 ±10.2	**Early subacute** 11 ±7.1 days	FMA-UE	FMA: 58.0 ±5.4 ARAT: n.a.	**aROM tasks** forward shoulder flexion, thumb−finger opposition, forward trunk flexion, hip abduction, and foot tapping	MRI/CT	not reported	lesion location	minimal / button press / touch screen	visuomotor task: tracing a circular trajectory (radius of 7 cm) viewed on a monitor using a cursor controlled by a wireless computer mouse	normalzed hand movement error, peak velocity	not simultaneous	O-C
**Saleh, 2014(161)**	15 (15/0)	S: 6/9	S: 54 ±12	**Chronic** > 6 months	CMA, WMFT	FMA: n.a. ARAT: n.a.	**aROM tasks** whole hand flex-ext, and finger flexion-extension	fMRI	3T Siemens Allegra and head-only scanner	BOLD contrast	minimal / sensor-based	MRI-compatible recording gloves (Fifth Dimension Technologies Inc, Pretoria, South Africa)	movement time, wrist flexion-extension angle, finger flexion-extension angle	simultaneous	O-C
**Salehi Dehno, 2022(162)**	19 (19/0)	S: 9/10	S: 51.6 ±11.3	**Subacute** 1-6 months	FMA-UE	FMA: 20.57 ±5.61 (range 4-29)	**Strength tasks** grip strength	TMS	Single TMS pulses (Neurosoft, Ivanovo, Russia)	rMT, aMT, TCI	medium / grip device	electronic dynamometer (Digital Pinch/Grip Analyser, MIE Medical Research, Leeds, UK)	grip strength, pinch force/strength	not simultaneous	O-C
**Sawaki, 2008(163)**	30 (30/0)	not reported	S_E: 54.4 ±3.8; S_C: 58.5 ±3.5	**Late subacute** 3-9 months	WMFT	FMA: n.a. ARAT: n.a.	**Grasp tasks** grasp, manipulation and release of objects	TMS	Magstim 200 stimulator (Magstim, Whitland, Dyfed, UK)	rMT, aMT, TMS map area, TMS map center, RC, SP	not available / not reported	force device not reported	grip strength	not simultaneous	I-L
**Schaechter, 2008(164)**	20 (10/10)	S: 4/6	S: 59 ±11	**Chronic** 2.6 ± 1.9 years	PPT	FMA: n.a. ARAT: n.a.	**Complex tasks** 5 digits in kinematically synergistic and nonsynergistic motor tasks	fMRI	3T Siemens Trio magnetic resonance scanner	ROI	minimal / sensor-based	microelectromechanical system (MEMS) gyroscopes to measure angular velocity of each of the 10 digits.	movement frequency, movement time, jerk metric, movement amplitude, mean acceleration, angular velocity	simultaneous	O-C
**Schaefer, 2007(165)**	26 (10/16)	S: 0/10, H: 0/16	S_{LHD}: 53.6±9.6, S_{RHD}: 59.8 ±13.2, H_L: 59.6 ±9.0, H_R: 67.6 ±9.2	**Chronic** S_{LHD}: 9.8 ±5.6; S_{RHD}: 7.8 ±6.3 years	FMI 100; apraxia (Haaland and Flaherty, 1984)	FMA: n.a. ARAT: n.a.	**Reach tasks** planar pointing task; grip strength	MRI/CT	Phillips Edge 1.5T scanner	left vs. right hemisphere stroke, lesion volume, lesion location	minimal / sensor-based	Flock of Birds (FoB) (Ascension-Technology) magnetic sixdegree-of-freedom (6-DOF) movement recording system	grip strength, absolute error, end-point error, movement time, variable error, index finger position, elbow position, shoulder position, peak velocity, acceleration duration, deceleration duration, peak acceleration	not simultaneous	O-C
**Schaefer, 2009(166)**	36 (14/22)	S_{LHD}: 0/7, S_{RHD}: 1/6, H_L: 8 /7, H_R: 10/11	S_{LHD} 58.1 ±11.1; S_{RHD} 60.3 ±10.4; H_L: 62.6 ±7.6; H_R: 61.6 ±8.6	**Chronic** S_{LHD}: 9.8 ±6.1, S_{RHD}: 8.2 ±5.6 years	FMA-UE, JTHF	FMA: S_{LHD} 29.3 ±19.1 S_{RHD} 12.3 ±16.6	**Reach tasks** planar reaching task	MRI/CT	Phillips Edge 1.5T scanner or CT scan with Phillips PQ 6000 scanner	left vs. right hemisphere stroke, lesion volume, lesion location	minimal / sensor-based	Flock of Birds (FoB)® (Ascension-Technology) magnetic 6-DOF) movement recording system	absolute error, reaction time, movement time, peak velocity	not simultaneous	O-C

**Schaffer, 2020(167)**	24 (16/8)	not reported	S_{LHD}: 63.3 ± 13.5, and S_{RHD} 63.2 ± 9.8, H: 65.4 ±10.2	not reported	FMA-UE	FMA: S_{LHD} 58.7 ±5.9, S_{RHD} 62.0 ±5.9 ARAT: n.a.	**Reach tasks** bimanual reach to target (move virtual bar)	MRI	not reported	left vs. right hemisphere stroke, lesion mask	medium / end-effector	Kinereach 2D VR, 6 DOF magnetic sensors (Ascension TrackStar) placed on the hand and upper arm.	mean end-point error, end-point error, interlimb coordination at movement end, distance traveled	not simultaneous	O-C
**Schranz, 2023(168)**	22 (22/0)	S: 8/14	S: 61.6 ±12.6	**Chronic** > 6months	FMA-UE	FMA: 43.9 ±11.9 ARAT: n.a.	**Strength tasks** hand grip performance	MRI	3T Prisma scanner (Siemens Healthcare, Erlangen, Germany)	weighted connectivity pairs	minimal / force sensor	thumb and index finger force sensors (Mini40, ATI Industrial Automation Inc, NC)	grip strength, force direction control, reaction time, relaxation time	not simultaneous	O-C
**Schulz, 2012(169)**	22 (13/9)	not reported	not reported	**Chronic** > 6months	not reported	not reported	**Strength tasks** grip strength	MRI	not reported	FA	medium / grip device	digital dynamometer, dynamic index finger and thumb with 2 force sensors	grip strength ratio	not simultaneous	O-C
**Schulz, 2015(170)**	45 (25/20)	S: 8/17, H: 10/10	S: 64 ±8.8, H: 67 ±2.4	**Chronic** >12months	FMA-UE, mRS	FMA: range 16-66	**Strength tasks** grip strength	MRI-DWI	3T Siemens Skyra MRI scanner	probabilistic tractography	not available / not reported	not reported	grip strength, pinch force/strength	not simultaneous	O-C
**Semrau, 2017(171)**	227 (227/0)	S_1: 17/25, S_2: 35/66, S_3: 23/61	S_1 60.7 ±14.4, S_2 57.8 ±15.9, S_3 65.7 ±12.8	**Acute** <1 week	CMSA, PPT, BIT	FMA: n.a. ARAT: n.a.	**Reach tasks** visually guided reaching (VGR) task	MRI/CT	General Electric 1.5T or 3T MRI scanner	lesion volume, cortical vs. subcortical stroke	high / robot-based	Kinarm robotic exosceleton uni and bilateral arm motions	movement time, reaction time, path length ratio, initial movement direction error, number of velocity peaks, peak velocity, movement velocity, posture speed	not simultaneous	O-C
**Shang, 2021(172)**	76 (76/0)	S_E: 21/16; S_C: 20/19	S_E: 60.6–66.7, S_C: 61.1–67.1	**Early subacute** ≤ 10 days	NIHSS, MoCA	FMA: n.a. ARAT: n.a.	**Strength tasks** grip strength	MRI	3T MR machine (Verio, Siemens, Germany)	FA	medium / grip device	electronic hand dynamometer (Model WCS10000, Wanqing Inc., Shanghai, China)	grip strength	not simultaneous	O-C
**Shin, 2008(173)**	14 (14/0)	S: 12/2	S: 57.6 ±9.5	**Chronic** 18.6±4.2, 19.7±7.7 months	BBT	FMA: n.a. ARAT: n.a.	**complex tasks** Tracking Test	fMRI, NMES, EMG-stim	1.5T magnetic resonance imaging scanner (Vision, Siemen, Germany)	BOLD contrast	minimal / sensor-based, sEMG	Double axis electrogoniometer (Biometrics Ltd., USA)	extensor digitorum communis, grip strength, root mean square error, delays in onset, delay in offset	simultaneous	I-L
**Small, 2002(174)**	12 (12/0)	S: 5/7	S: 54 (44-74)	**Early subacute** < 1months	NHPT	FMA: n.a. ARAT: n.a.	**aROM tasks** index-thumb opposition, wrist flexion-extension, grip strength	fMRI	not reported	BOLD contrast	medium / grip device, minimal / sEMG	dynamometer, EMG on extensor digitorum communis	flexor digitorum superficialis, extensor digitorum communis, pinch force/strength, grip strength	simultaneous	O-L
**Stagg, 2012(175)**	17 (17/0)	S: 4/13	S: 64 ±13.7	**Chronic** >6months	FMA-UE	FMA: 47.1 ±16.9 ARAT: n.a.	**Complex tasks** wrist flexion-extension in response to circle or square	fMRI, tDCS	3T Siemens/ Varian MRI system, DC-Stimulator (Eldith GmbH)	BOLD contrast	medium / end-effector	joystick with a maximum of 10degrees wrist ext	grip strength, response time	simultaneous	I-C

**Stewart, 2014(176)**	20 (14/6)	S_{RHD}: 4/2 S_{LHD}:5/3 H: 4/2	S_{RHD}: 59.5, S_{LHD}: 58.9 H: 63.8 ±14.4	**Chronic** > 6 month	FMA-UE, ARAT	FMA: S_{RHD} 54.8, S_{LHD} 51.0 ARAT: S_{RHD} 46.7; S_{LHD} 42.9	**Reach tasks** movement distance for contralesional and ipsilesional targets	not reported	not reported	left vs. right hemisphere stroke, lesion volume, lesion location	minimal / camera-based	immersive virtual display (Innovative Sport Training, Inc., Chicago, IL) with marker at index finger	end-point error, movement time, distance traveled, time to peak velocity, peak velocity, peak acceleration	not simultaneous	O-C
**Stewart, 2017(177)**	11 (11/0)	S: 5/6	S: 60.3	**Early subacute** >3 months	FMA-UE, ARAT	FMA: 54.2 ±7.6 ARAT: 46.9	**Reach tasks** planar reaching task	MRI-DTI	3T GE MRI scanner (General Electric, Fairfield, CT)	mean FA	minimal / sensor-based	immersive virtual environment (VE) (Innovative Sports Training, Inc, Chicago, IL)	end-point error, movement time, peak velocity, peak acceleration	not simultaneous	O-C
**Stewart, 2022(178)**	16 (16/0)	S: 5/11	S: 56.1	**Chronic** >6months	FMA-UE, BBT, SIS hand domain	FMA: 43.3 (range 18-61) ARAT: n.a.	**Complex tasks** motor action selection task using joystick with rules (e.g. small blocks / big circles from left to right / left to right)	fMRI	3T Siemens Prisma MRI scanner	BOLD contrast	medium / end-effector / touch screen	joystick	reaction time, movement direction, movement amplitude, peak velocity	simultaneous	I-L
**Stinear, 2007(179)**	21 (21/0)	S: 6/15	S: 54 ±12	**Chronic** > 6 months	FMA-UE	FMA: 16 (4-25) ARAT: n.a.	**aROM tasks** hand grip hand opening and closing, uni- and bilateral (fMRI)	TMS MRI fMRI	MagStim 200 stimulator (MagStim Company, Dyfed, UK) 1.5T MRI system Siemens Magnetom Avanto	rMT, MEP presence, MEP amplitude, mean MEP latency, FA asymmetry, BOLD contrast	medium / grip device	custom-built force transducer saline drip bag connected to a pressure transducer	grip strength, force/strength asymmetry	not simultaneous / simultaneous	O-L
**Strens, 2004(180)**	41 (25/16)	S: 5/20, H: 5/11	S: 54 ±13.1, H: 51.3 ±11.4	**Chronic** >12months	mRS, BI, MRC	FMA: n.a. ARAT: n.a.	**Strength tasks** grip strength at 20-25%MVC	EEG	64 electrodes scalp EEG cap (FMS Easy Cap, Falk Minow Services, Herrsching-Breitbrunn, Germany)	EEG-EEG coherence, task-related coherence, task-related power	medium / force sensor minimal / sEMG	grip force transducer with aluminum in U-shape, EMG	flexor carpi radialis, flexor carpi ulnaris, extensor carpi radialis, extensor carpi ulnaris, first dorsal interosseous, abductor pollicis brevis, grip strength	simultaneous	O-C
**Sugarman, 2002(181)**	16 (11/5)	S: 2/9, H: 1/4	S: 66.1, H: 67.4	**Chronic** 1-3 years	not reported	FMA: n.a. ARAT: n.a.	**aROM tasks** finger-flexion extension in frontal plane	not reported	not reported	left vs. right hemisphere stroke	high / robot-based	linear potentiometer, finger flexion-extension apparatus	movement time, percentage of time spent in pause, number of motion pauses, velocity profile	not simultaneous	O-C
**Summers, 2007(182)**	12 (12/0)	S_E 2/4, S_C 3/3	S_E 63 ±16, S_C 60 ±14	**Chronic** > 6 months	MAS	FMA: n.a. ARAT: n.a.	**Grasp tasks** dowel lifting and placement task	TMS	TMS (Magstim 200, Magstim Co., Whitland, Dyfed, UK)	MEP amplitude, TMS map area	minimal / camera-based	OPTOTRAK three-dimensional motion analysis system tracking infrared emitting diodes (IREDS)	curvature of arm trajectories, number of movement onset, number of movement offset, movement time, elbow flexion-extension angle, movement velocity	not simultaneous	I-L

**Sun, 2018(183)**	24 (24/0)	S: 6/18	not reported	**Chronic** > 6 months	FMA-UE, WMFT	FMA: 26.2 ±20.6 ARAT: n.a.	**Strength tasks** max voluntary contraction of wrist extension isometric	TMS spinal cord and cortical plasticity	Magstim 2002 stimulator unit with coil (P/N 9790, Magstim Co. Ltd., Whitland, Carmarthenshire, UK)	SP, TCI, SICI, ICF	medium / force sensor	6-axis force sensor (ATI, IndustrialnAutomation Gamma DAQ F/T Transducer, Apex, NC, USA).	wrist flexion-extension force/torque	not simultaneous	I-L
**Szameit at, 2012(184)**	26 (5/21)	S: 2/3, H: 10/11	S: 62 H: 25	**Chronic** > 6 months	handedness, SIS, Frenchay arm test, mAS	FMA: n.a. ARAT: n.a.	**aROM tasks** wrist flex-ext (maximal extension until 40 degree)	fMRI	3T scanner (Trio, Siemens, Erlangen, Germany)	BOLD contrast	medium / force sensor, end-effector	moveable flap with two non-magnetic force sensors	wrist flexion-extension angle	simultaneous	O-C
**Taga, 2021(5)**	31 (16/15)	S: 10/6; H: 7/8	S:62 (35−85), H:54 (36−81)	**Chronic** >6 months	FMA-UE, visual analogue scale	FMA: S 49.3 (23-64) H:, 65.0 (63−66) ARAT: n.a.	**Strength tasks** MVC, 20-45% MVC isometric for BIC and FDI	TMS	Magstim 2002 stimulator (Magstim Company Limited), remote-control coil (max output 2.2T; Magstim Company Limited, Whitland, UK)	MEP presence, rMT, aMT	medium / grip device	dynamometer	force production	simultaneous	O-C
**Takahashi 2008(186)**	13 (13/0)	S: 7/6	S: 63 ±16	**Early subacute** < 3 months	FMA-UE, NIHSS, ARAT, BBT, NHPT, SIS	FMA: 44.6 ±10.4 ARAT: 34 ±20	**aROM tasks** hand opening /closing	fMRI	1.5T Philips scanner	BOLD contrast	high / grip device robot-based, minimal / sEMG	Hand Wrist Assistive Rehabilitation Device (HWARD) pneumatically 3DOF paired with three muscles, dynamometer, plastic non-actuated robot copy	extensor carpi radialis, extensor carpi ulnaris, biceps brachii, grip strength, pinch force/strength	simultaneous	I-L
**Takeuchi, 2008(187)**	20 (20/0)	S_E: 2/8, S_C: 2/8	S_E: 61.2 ±9.7, S_C: 63.4 ±7.4	**Chronic** > 6 months	FMA-UE	%of max FMA: S_E 65.7 ±18.5, S_C 69.2 ±21.2 ARAT: n.a.	**aROM tasks** Thumb movement, pinch force	rTMS	Magstim 200 (Magstim Co., dyfed, uK)	rMT, MEP amplitude, ICI	medium / pinch gauge, minimal / sensor-based	pinch gauge (Pinch Meter SPR-641; Sakai Medical, Tokyo, Japan), electrodes, accelerometer (model MP110-10-101; Medisens, Sayama, Japan)	pinch force/strength, grip strength, peak acceleration	not simultaneous	I-L
**Takeuchi, 2005(188)**	20 (20/0)	S_E: 3/7 S_C: 2/8	S: 59.0 ±9.6	**Chronic** 6-60 months	FMA-UE	S_E: 59.9 ±21.2, S_C: 63.7 ±24.7 ARAT: n.a.	**Strength tasks** pinch force	rTMS	Magstim 200 for single TMS and a Magstim Rapid stimulator (Magstim Company	MEP amplitude	medium / pinch gauge minimal / sensor-based	pinch gauge (Pinch Meter SPR-641; Sakai Medical), monoaxial accelerometer (model MP110-10-101, Medisens)	pinch force/strength, thumb acceleration	simultaneous	I-L

**Takeuchi, 2007(189)**	20 (20/0)	S: 7/13	S: 52.5 ±14.4	**Chronic** 27.4 ±18.2 months	FMA-UE	%of max FMA: 65.1 ±4.3 ARAT: n.a.	**Complex tasks** finger flex reaction time task	TMS	Magstim 200 stimulator (Magstim Co, Dyfed, UK)	rMT	minimal / button press / touch screen	Labview (National Instruments, Austin, Tex)	reaction time	simultaneous	O-C
**Taylor, 2012(190)**	28 (13/15)	S: 2/11, H: not reported	S 67 ±16; C 29.8 ±6	**Chronic** > 1 year	FMA-UE, BBT	FMA: 53 ±12.3 ARAT: n.a.	**aROM tasks** wrist extension	TMS	Magstim 2002; Magstim Company, Wales, UK	MEP amplitude, MEP latency, rMT, ICI, ICF	minimal / force sensor, sEMG	force transducer	extensor carpi radialis, wrist flexion-extension force/torque	simultaneous	I-L
**Thickbroom, 2002(191)**	23 (23/0)	S: 6/17	S: 56.1 ± 14.6	**Early subacute** 1 months	MRC, MAS, McCarron test battery	FMA: n.a. ARAT: n.a.	**Strength tasks** grip strength	TMS	Magstim 200 stimulator with a 50 mm figure-of-8 coil	MEP amplitude, MEP threshold	medium / grip device	hand-held dynamometer (Sammons Preston, Bolingbrook, IL)	grip strength ratio	not simultaneous	O-C
**Thickbroom, 2004(192)**	67 (27/40)	S: 8/19, H: not reported	24-72	**Chronic** ≥ 6 months	Motor Assessment Scale	FMA: n.a. ARAT: n.a.	**Strength tasks** grip strength	TMS	Magstim 200 stimulator with a 50 mm figure-of-eight coil	aMT, MEP amplitude, MEP latency, peak-to-peak amplitude, TMS map center, TMS map area	medium / grip device	hand-held dynamometer (Sammons Preston, Bolingbrook, IL)	grip strength	simultaneous	O-C
**Tian, 2021(193)**	16 (8/8)	S: 2/6, H: 2/6	S: 64.4 ±8, H: 60.1 ±7.5	**Chronic** 11.1 ±7.1 years	FMA-UE	FMA: 21.5 ±10 (range 10-40) ARAT: n.a.	**Strength tasks** Shoulder Abd at 20 or 40% of maximum voluntary torque (MVT)	EEG	32-channel EEG with active electrodes (Biosemi, Inc, Active II, Amsterdam, the Netherlands)	corticomuscular coherence (CMC), cross-spectral connectivity (CSC), laterality index	high / force sensor and cast	cast by fiberglass to line up the forearm, wrist, and hand and then attached to a beam connected to a JR3 6-DOF load cell (JR3, Woodland, CA).	shoulder abduction-adduction force/torque, elbow flexion-extension force/torque	simultaneous	O-C
**Tilsley, 2021(194)**	19 (3/16)	S: 1/2, H_A: 3/5, H_B: 0/8	S: 29-50, H_A: 52 ± 4, H_B: 29 ± 4	not reported	not reported	FMA: n.a. ARAT: n.a.	**Reach tasks** arm reaching movements toward visual target, back to the start	MRI	not reported	corpus callosum stroke	minimal / camera-based	optical motion tracking system (Codamotion cx1 and MiniHub, Charnwood Dynamics Ltd)	end-point error, end-point direction error, trajectory error, movement time, reaction time, initial movement direction, time to peak velocity, peak velocity	not simultaneous	O-C
**Tombari, 2004(195)**	18 (8/10)	S: 3/5, H: 7/3	S: 62 ±11; H: 49 ±7	**Early subacute** 20 ± 9 days	mAS, MI UE	FMA: n.a. ARAT: n.a.	**aROM tasks** finger tapping, grip strength	fMRI	1.5T scanner (Siemens Vision, Erlangen, Germany)	BOLD contrast	medium / grip device	hand dynamometer	grip strength, finger tapping	simultaneous	O-L
**Tscherpel, 2020(196)**	30 (14/16)	S: 6/8, H: 3/13	S: 70.3 ±9.3, H: 65.1 ±9.1	**Acute** 3.6 ±1.7 days	NIHSS	FMA: n.a. ARAT: n.a.	**aROM tasks** finger tapping, hand tapping, and index pointing during TMS	TMS	Magstim Super Rapid2 stimulator (The Magstim Co. Ltd, Whitland, United Kingdom)	rMT	minimal / sensor-based	Zebris CMS20 motion analyser system (Zebris Medical Company, Isny, Germany)	movement amplitude, peak velocity, tapping and pointing velocity,	simultaneous	O-L
**Turolla, 2013(197)**	15 (15/0)	S: 8/7	S: 54 ±18.8	**Chronic** < 6months	FMA-UE, NHPT	FMA: 45.7 ±12.9 ARAT: n.a.	**aROM tasks** index finger flex-ext, both alternating, VR tracking task discrete and continuous	fMRI	1.5T Philips (Achieva) scanner	BOLD contrast	minimal / sensor-based	haptic finger / hand device with virtual reality arranged in a planar way	mean movement time, jerk metric, normalized jerk, mean velocity	not simultaneous	I-L

**Urbin, 2021(198)**	14 (14/0)	S: 3/11	S: 64.8 ±6.8	**Chronic** 21-244 months	MMT scale	FMA: n.a. ARAT: n.a.	**Strength tasks** pinch force	TMS FES	Magstim 200 (The Magstim Company Ltd, Whitland, UK), constant-current stimulator (DS7R, Digitimer Ltd, Welwyn Garden City, UK)	MEP latency, spike timing-dependent plasticity, FES cortical stimulation	minimal / force sensor	triple stimulation force sensor (Mini-40, ATI Industrial Automation, Apex, NC,USA range ±2400 N)	pinch force/strength	simultaneous	O-C
**Vahdat, 2019(199)**	10 (10/0)	S: 2/8	S: 62.5 ±8.8	**Chronic** > 6months	FMA-UE, NSA	FMA: 32 ±26.7 ARAT: n.a.	**Reach tasks** Reaching movement	fMRI	3T Siemens Trio MR scanner	FC	medium / end-effector	In Motion2, Interactive Motion Technologies	trajectory error, end-point error, hand position, peak velocity	not simultaneous	I-C
**van Assche, 2021(200)**	32 (16/16)	S: 6/10 H: 6/10	S: 72.9 ±11.9, H: 70.3 ±9.7	**Early subacute** <10 days for 1visit	FMA-UE, NIHSS, NHPT	FMA: 62.31 ±7.05 ARAT: n.a.	**Strength tasks** grip strength	MRI	3T MRI (Magnetom Prisma, Siemens, Erlangen, Germany; 64-channel head-coil)	lesion mask, FC	medium / grip device	(JAMAR dynamometer, Asimow Engineering Co., Los Angeles, CA)	grip strength	not simultaneous	O-C
**van Dokkum, 2018(201)**	34 (21/13)	S: 6/15 H: not reported	S: 59 ±13.2	**Chronic** 19 ±12 months	FMA-UE, BBT	FMA: 11.6 ±9.1 ARAT: n.a.	**aROM tasks** elbow flexion/extension task of ipsilesional	fMRI	1.5T whole-body MRI system (Magnetom Avanto, Siemens, Erlangen, Germany), with 12-channel receive-only head coil	BOLD contrast	minimal / camera-based	1.5-T MRI compatible 3D ultrasound motion capture system (Zebris, CMS-20, Medical GmbH, Isny, Germany)	movement frequency, trajectory path length, number of velocity peaks, movement amplitude	simultaneous	O-L
**Velicki, 2000(202)**	12 (6/6)	S: 2/4 H: 4/2	S: 62.7 ±8.9, H: 62.5 ±8.0	**Chronic** > 6months	not reported	FMA: n.a. ARAT: n.a.	**Reach tasks** reaching to 4 targets (short and long by elbow flex-ext) with time-response paradigm	MRI/CT	not reported	lesion location	medium / end-effector	instrumented manipulandum, IBM compatible CPU, and monitor (with lever potentiometer)	constant error, absolute error, stimulus-to-response interval, response time, initial movement direction error, hand position	not simultaneous	O-C
**Verleger, 2003(203)**	21 (13/8)	S: 7/6, H: 4/4	S: 65 ±8, H:62 (53-73)	**Chronic** 20 months–9 years	not reported	FMA: n.a. ARAT: n.a.	**Strength tasks** key press with both index fingers, spontaneous force, graded force	EEG	26 EEG electrode	CNV amplitude, movement-accompanying negativity	minimal / force sensor	force sensitive keys, index finger, sampled with 100 Hz from 0.1 s before to 3.9 s after the cue (1.9 s after IS)	force-related response time, finger flexion-extension force/torque, error types, movement time, slope between reaction time and peak force	simultaneous	O-C
**Volz, 2016(204)**	26 (26/0)	S: 9/17	S: 67.2 ±13.1	**Acute** 7.3 ±3.6 days	JTT	FMA: n.a. ARAT: n.a.	**aROM tasks** visually cued rhythmic fist closures, grip strength	fMRI TMS, iTBS	3T scanner (Siemens Trio, Erlangen, Germany), Magstim 2002 stimulator (The Magstim Co. Ltd, Whitland, UK)	BOLD contrast, rMT, MEP amplitude	not available / not reported	not reported	number of successful trials	simultaneous	I-L

**Volz, 2017(205)**	26 (12/14)	S: 4/8, H: 9/5	S: 69.3 ±11.4, H:61.8 ±6.6	**Acute** 5.0 ± 3.2days	NIHSS	FMA: n.a. ARAT: n.a.	**aROM tasks** maximum finger tapping and grip strength task	TMS MRI-DWI	Magstim SuperRapid2 stimulator (The Magstim Co. Ltd., Whitland, UK), 1.5 T MRI scanner (Philips, Guildford, UK)	rMT, lesion mask (used in VLSM)	minimal / button press, medium / grip device	computer keyboard button dynamometer	grip strength, movement frequency, speeded movement initiation	simultaneous	I-L
**Vongvaivanichakul, 2014(206)**	14 (14/0)	not reported	S: 57.8	**Chronic** > 6months	FMA-UE	FMA: 45.1 ±3.7 ARAT: n.a.	**Grasp tasks** reach-to-grasp activities	rTMS	Magstim rapid(2) (Magstim Co., Dyfed, UK)	CSE, rMT	medium / force sensor	metronome for timing, blood pressure cuff for feedback and grasp force set at 10 mmHg	abductor pollicis brevis, grip strength, movement time, peak grip aperture	not simultaneous	I-C
**Wang, 2011(207)**	22 (11/11)	S: 1/10, H: 3/8	S: 62.4 ±10.9, H: 63.1	**Acute** 4-275 weeks	ARAT	FMA: n.a. ARAT: S_E 42±12, S_C 43±11	**reach tasks** finger tapping index for 4s, 3times, grip strength, finger pointing (during fMRI)	fMRI	3T Siemens Trio scanner (Erlangen, Germany)	BOLD contrast, ROI	minimal / observer counts	observer counted inside scanner room, vigorimeter, Zebris CMS20S system (Zebris Medical GmbH, Isny, Germany)	number of successful trials, movement frequency, movement amplitude, grip strength, movement frequency, movement amplitude	simultaneous	I-L
**Ward, 2003a(208)**	46 (20/26)	S: 3/17, H: 9/17	S: 53.2 ±14.5, H: 47.6 ±15.8	**Late subacute** > 3months	ARAT, NHPT, mAS, BI, mRS	FMA: n.a. ARAT: 11.36	**Strength tasks** grip strength, isometric; surface EMG	fMRI	2T Siemens Vision system (Siemens, Erlangen, Germany)	BOLD contrast	medium / grip device, minimal / sEMG	manipulandum, EMG	biceps brachii, triceps brachii, latissimus dorsi, flexor carpi radialis, flexor carpi ulnaris, grip strength, associated movements, mirror movements	simultaneous	O-C
**Ward, 2003b(209)**	12 (8/4)	S: 1/7, H: 2/2	S: 53.2 ±14.5, H: 47.6 ±15.8	**Early subacute** 10±14 days	ARAT, NHPT	FMA: n.a. ARAT: 47.6 ±15.8	**Strength tasks** Isometric hand gripping task	fMRI	2T Siemens VISION system (Siemens, Erlangen, Germany)	BOLD contrast, lesion mask	medium / grip device	manipulandum with 2 force transducers (Honeywell FSG15N1A; Honeywell, NJ, USA) between 2 plastic bars	grip strength	simultaneous	O-L
**Ward, 2007(210)**	18 (9/9)	S: 0/9, H: 0/9	S 48.1 ±14.3, H 49.3 ±15.6	**Late subacute** 4-116 months	NHPT	FMA: n.a. ARAT: n.a.	**Strength tasks** grip task, grip strength	fMRI TMS	3T Siemens ALLEGRA system (Siemens, Erlangen, Germany) TMS not reported	BOLD contrast, MEP amplitude, motor response M-wave	medium / grip device, sEMG	MRI-compatible manipulandum, manipulandum, EMG	grip strength, first dorsal interosseous	simultaneous	O-C
**Watanabe, 2018(211)**	21 (21/0)	S: 7/14	S_{E1}: 72.5, S_{E2}: 67.6, S_C: 75.2	**Acute** < 7days	FMA-UE, SIAS, mAS	S_{E1}: 14 (2–38), S_{E2}: 8 (4–30), S_C: 2.5 (0–39)	**Strength tasks** grip strength	rTMS, iTBS	Magstim Rapid Square System, (Magstim Inc., Whitland, UK)	MEP amplitude	medium / grip device	mechanical hand dynamometer in kg	grip strength	not simultaneous	I-L
**Weitnauer, 2021(212)**	122 (55/67)	S: 20/25, H: 23/44	S: 46.7 ±13.9; H: 44.3 ±11.3	**Chronic** 10.6 ±10.8 months	not reported	FMA: n.a. ARAT: n.a.	**Strength tasks** gripping task	MRI	3T Trio (Siemens Medical Systems, Germany), 3T Magnetom Prisma (Siemens Medical Systems, Germany) with	voxel-based morphometry, lesion location, lesion volume	medium / grip device	Seahan Hydraulic Hand Dynamometer (SH5001)	grip strength	not simultaneous	O-C

									64-channel head coil						
**Weller, 2006(213)**	45 (36/9)	S: 17/19 H: 7/1	S: 64 ±10	**Acute** < 6hrs poststroke	ESS	FMA: n.a. ARAT: n.a.	**Strength tasks** pinch precision grip movement	MRI-DWI	SIEMENS scanner, Erlangen, Germany	lesion volume, lesion location, diffusion/perfusion mismatch volume	medium / grip device	piezoelectric pinch grip testing device of 120 g with 2 aluminum branches	movement frequency	not simultaneous	O-L
**Wenzelburger, 2005(214)**	36 (18/18)	S: 5/13, H: age, sex matched	S: 60.9 (40-81); H: age/sex matched	**Chronic** 2.4 ±1.9 years	MRC, MAS, FIM, RMA, CUE	FMA: n.a. ARAT: n.a.	**Reach tasks** reaching task, grip force	MRI/CT	not reported	lesion mask	minimal / camera-based	passive infrared movement analysis system (Mac Reflex 3.2; Qualisys, Sweden)	grip strength, peak velocity, acceleration duration, deceleration duration	not simultaneous	O-C
**Whitall, 2011(215)**	111 (111/0)	S_E: 16/26, S_C: 26/24	S_E: 59.8 ±9.9, S_C: 57.7 ±12.5	**Chronic** > 6 months	FMA-UE, WMFT	FMA: S_E: 32.3 ±2.2, S_C: 31.0 ±2.1	**aROM tasks** elbow flexion-extension 45° to 60° to 75°	fMRI	1.5 T scanner (Philips, Eindhoven, The Netherlands)	ROI	minimal / camera-based medium / grip device	video monitor, 2 cameras, Kincom dynamometer (Chattanooga, TN), Chatillon dynamometer (The Scale People, Maryland), hydraulic hand dynamometer (Kom Kare, NY)	elbow flexion-extension force/torque, shoulder flexion-extension force/torque, wrist flexion-extension force/torque, grip strength	simultaneous	I-L
**Wiese, 2005(216)**	24 (12/12)	S: 0/12, H: not reported	S: 61.8 ±5.5, H: 55.9 ±5.7	**Chronic** 12-51 months	MRC	FMA: n.a. ARAT: n.a.	**aROM tasks** self-initiated brisk abductions of their right index finger	EEG	30-channel EEG, referenced to linked earlobe electrodes (A1/A2), vertical electro-oculogram (EOG)	movement-related potential	minimal / sEMG	EMG on right index finger (m. interosseus)	first dorsal interosseous	simultaneous	O-C
**Wilkins, 2020(217)**	29 (17/12)	S: 3/14, H: 5/7	S: 58.9 ±7.6; H: 59.8 ±7.7	**Chronic** 11.2 ±7.1 years	FMA-UE	FMA: 20.8 ± 8.4 ARAT: n.a.	**Complex tasks** hand opening and closing with sh-abd load ACT-3D	EEG MRI	160-channel HD system active electrodes fabric cap (Active II; Biosemi, Inc., Amsterdam, The Netherlands), 3T scanner with 64-channel head coil (Siemens Prisma, Munich, Germany)	laterality index, power analysis, grey matter volume	minimal / sEMG high / robot-based	EMG, robotic device (ACT3D) instrumented with a six degree of freedom load cell (JR3 Inc., Woodland, CA, USA)	extensor digitorum communis, flexor carpi radialis, grip strength, maximum grasping pressure at rest, hand opening ability	simultaneous	O-C
**Winstein, 1995(218)**	30 (20/10)	S_{RHD}: 4/6, S_{LHD}: 5/5, H: 8/2	S_{RHD}: 60.3 ±8.2, S_{LHD}: 62.1 ±8.9, H: 67.2 ±8.3	**Chronic** mean 3.3 years (S_{RHD} 1.3-7.2, S_{LHD} 0.5-9)	not reported	FMA: n.a. ARAT: n.a.	**Reach tasks** planar arm reaching in two distances and target sizes as fast as possible	not reported	not reported	left vs. right hemisphere stroke	minimal / button press / touch screen	timer, hand-held stylus, a tapping board (Lafayette Instruments 32012), impulse counter (Lafayette Instruments 58022), video-taped Pulnix camera (model TM-640)	number of errors, movement time, distance traveled, peak velocity	not simultaneous	O-C

**Wittmann, 2001(219)**	33 (33/0)	S: 10/23	S: 50	**Late subacute** 3 months	hemiparesis yes/no	FMA: n.a. ARAT: n.a.	**aROM tasks** finger tapping (preferred and max speed)	not reported	not reported	left vs. right hemisphere stroke, cortical vs. subcortical stroke	minimal / button press / touch screen	button press (not specified)	inter response intervals, preferred tapping speed, peak velocity	not simultaneous	O-C
**Wolbrecht, 2018(220)**	26 (26/0)	S: 6/20	S: 58.3 ±13.3	**Chronic** 31.7 ±48.2 months	FMA-UE, BBT, NHPT	FMA: 47.0 ±11.6;	**Grasp tasks** index and middle finger force and individuation	MRI	3T Philips scanner (Best, the Netherlands) Achieva system	CST injury	high / robot-based	FINGER robotic exoskeleton	finger flexion-extension force/torque, peak force/torque	not simultaneous	O-L
**Woldag, 2008(221)**	30 (15/15)	S: 7/8, H: 11/4	S: 59.1 ±12.4 H: 47.3 ±6.9	**Early subacute** 20-136 days	MRC, MI	FMA: n.a. ARAT: n.a.	**aROM tasks** isotonic and isometric voluntary wrist extensions	rTMS	Magstim Rapid for 25 minutes (1500 pulses)	rMT	not available / not reported	not reported	10, 20, 30% MVC	simultaneous	O-C
**Woldag, 2004(222)**	21 (11/10)	S: 7/4 ; H: 5/5	S: 62.7 43-81) ; H: 26 (24-34)	**Early subacute** 4-14 weeks	RMA arm section 0-15	FMA: n.a. ARAT: n.a.	**Strength tasks** index finger abduction, pinch grip, power grip	TMS	Magstim 200 magnetic stimulator (Magstim Company Ltd)	MEP amplitude	medium / strain gauge	strain-gauge for index finger abduction	index finger abduction force level	simultaneous	O-C
**Xi, 2022(223)**	56 (27/29)	S: 13/14, H: 13/16	S: range:29-66, H:24-60	**Early subacute** 1-2 months	NIHSS	FMA: n.a. ARAT: n.a.	**Strength tasks** 15, 25 and 50% MVC grip strength	EEG	64-channel (NeuSen.W64, Neuracle, China) EEG signals	functional CMC, CMC	medium / grip device	spring-loaded grip meter (China, Lynx Mall, EH101)	flexor digitorum superficialis, brachioradialis, biceps brachii, extensor digitorum, flexor carpi ulnaris, extensor carpi ulnaris, grip strength	simultaneous	O-C
**Zemke, 2003(224)**	35 (21/14)	not reported	not reported	**Early subacute** >10 weeks	FMA	FMA: 62.7 ARAT: n.a.	**aROM tasks** finger tapping	fMRI	not reported	BOLD contrast, PSC, laterality index	medium/ splint + force sensor	Wrist splint + force transducer	finger tapping frequency, frequency, range of motion, muscle activity	simultaneous	O-C
**Zhang, 2022(225)**	42 (42/0)	S_1: 5/9, S_2: 7/7, S_3: 6/8	S_1: 58.2 ±9, S_2: 59.5 ±8.6, S_3: 64 ±5.4	**Chronic** > 6 months	FMA-UE, ARAT	FMA: S_1 33.9 ±15.5, S_2 40.4 ±20.1, S_3 38.2 ±16.2 ARAT: S_1 17 ±14.3, S_2 24.6 ±20.1, S_3 22.3 ±21.8	**aROM tasks** finger tapping in S1 (primed iTBS), S2 (non-primed iTBS) and S3 (sham stimulation)	EEG TMS-TBS	EEG not reported, Standard 600-pulse TBS6 using the MagPro X100 stimulator (MagVenture, Denmark)	ERD, rMT, beta band frequency	high / robot-based, button press / touch screen	Keyboard button for tapping, end-effector robot (ArmMotus, Fourier Intelligence Co. Ltd, Shanghai)	mean velocity	simultaneous	I-L

Legend: AMAT, Arm Motor Ability Assessment; aMT, active motor threshold; ARAT, Action Research Arm Test; ADC, apparent diffusion coefficient; BBT, box and blocks test; BCI, brain computer interface; BDI, Beck Depression Inventory; BI, Barthel index; BIT, Behavioral Inattention Test; BOLD, blood oxygen level dependent; BS, Brunnstrom stages; CE, corticomotor excitability; CMC, corticomuscular coherence; CMCT, central motor conduction time; CMSA, Chedoke-McMaster Stroke Assessment; CNV, contingent negative variation; COPM, Canadian Occupational Performance Measure; $_C$, control group; CSE, corticospinal excitability; CUE, Capability of Upper Extremity Test; DOF, degree of freedom; DTI, diffusion tensor imaging; DWI, diffusion weighted imaging; $_E$, experimental group; EEG, electroencephalogram; EHI, Edinburgh Handedness Inventory; EmNSA, Erasmus-modified Nottingham Sensory Assessment; ERD, event-related desynchronization; ERP, event-related potential; ERS,

event-related synchronization; ESS, European Stroke Scale; f, functional; FA, fractional anisotropy; FC, functional connectivity; FIM, Functional Independence Measure; FMA-UE, Fugl-Meyer Assessment of the Upper Extremity; FMI, Fugl-Meyer Index; H, healthy subjects; HbO, oxyhemoglobin; HHb, deoxyhemoglobin; I-C, cross-sectional interventional study; ICARS, International Cooperative Ataxia Rating Scale; ICF, intracortical facilitation; ICI, intracortical inhibition; I-L, longitudinal interventional study; IMC, intermuscular coherence; IPS, intraparietal sulcus; JTHF, Jebsen-Taylor Hand Function Test; LL, lesion load; MAL, Motor Activity Log; mAS, modified Ashworth Scale; MAS, Motor Assessment Scale; mBI, modified Barthel index; MEP, motor evoked potential; MFT, Manual Function Test; MI, motricity index; MMT, Manual Muscle Testing; MRC, Medical Research Council; MRCP, movement-related cortical potential; MRI, magnet resonance imaging; MoCA, Montreal Cognitive Assessment; mRS, modified Rankin Scale; MVC, maximum voluntary contraction; NIHSS, National Institute of Health Stroke Scale; NIRS, near-infrared spectroscopy; NSA, Nottingham Sensory Assessment, O-C, observational cross-sectional study; O-L, observational longitudinal study; PPT, Purdue Pegboard Test; PSC, percentage signal change; RC, recruitment curve; rMT, resting motor threshold; RMA, Rivermead Motor Assessment; ROI, region of interest; RP, readiness potential; rs, resting-state; S, stroke subjects; SAI, short latency intracortical inhibition; SARA, Scale for Assessment and Rating of Ataxia; SIAS, Stroke Impairment Assessment Set; SICI, short-interval intracortical inhibition; SIS; Stroke Impact Scale; SMR, sensorimotor rhythm; SP, silent period; SSS, Scandinavian Stroke Scale; T, tesla; tACS, transcranial alternating current stimulation; TCI, transcallosal inhibition; tDCS, transcranial direct current stimulation; TBS, theta burst stimulation; TCD, transcranial doppler ultrasound; TMS, transcranial magnetic stimulation; VLSM, voxel lesion symptom mapping; WMFT, Wolf Motor Function Test.

UL measurement systems were categorized into minimal (e.g., sensor-based or camera-based systems), medium (e.g., end-effector systems) and high (e.g., exoskeletons or splints with weight support) influence groups based on their presumed level of interaction forces with the person being assessed. “Time since stroke” refers to the time post-stroke at study entry.

3. REFERENCES

1. Sacco RL, Kasner SE, Broderick JP, Caplan LR, Connors JJ, Culebras A, et al. An updated definition of stroke for the 21st century: a statement for healthcare professionals from the American Heart Association/American Stroke Association. Stroke. 2013;44(7):2064-89.
2. Bernhardt J, Hayward KS, Kwakkel G, Ward NS, Wolf SL, Borschmann K, et al. Agreed definitions and a shared vision for new standards in stroke recovery research: The Stroke Recovery and Rehabilitation Roundtable taskforce. Int J Stroke. 2017;12(5):444-50.
3. Schwarz A, Kanzler CM, Lambercy O, Luft AR, Veerbeek JM. Systematic Review on Kinematic Assessments of Upper Limb Movements After Stroke. Stroke. 2019;50(3):718-27.
4. Whiting PF, Weswood ME, Rutjes AW, Reitsma JB, Bossuyt PN, Kleijnen J. Evaluation of QUADAS, a tool for the quality assessment of diagnostic accuracy studies. BMC Med Res Methodol. 2006;6:9.
5. Whiting PF, Rutjes AW, Westwood ME, Mallett S, Deeks JJ, Reitsma JB, et al. QUADAS-2: a revised tool for the quality assessment of diagnostic accuracy studies. Ann Intern Med. 2011;155(8):529-36.
6. Ackerley SJ, Stinear CM, Barber PA, Byblow WD. Combining theta burst stimulation with training after subcortical stroke. Stroke. 2010;41(7):1568-72.
7. Ackerley SJ, Stinear CM, Barber PA, Byblow WD. Priming sensorimotor cortex to enhance task-specific training after subcortical stroke. Clin Neurophysiol. 2014;125(7):1451-8.
8. Altenmuller E, Marco-Pallares J, Munte TF, Schneider S. Neural reorganization underlies improvement in stroke-induced motor dysfunction by music-supported therapy. Ann N Y Acad Sci. 2009;1169:395-405.
9. Ambron E, Jax S, Schettino LF, Coslett HB. Magnifying vision improves motor performance in individuals with stroke. Neuropsychologia. 2018;119:373-81.
10. Ameli M, Grefkes C, Kemper F, Riegg FP, Rehme AK, Karbe H, et al. Differential effects of high-frequency repetitive transcranial magnetic stimulation over ipsilesional primary motor cortex in cortical and subcortical middle cerebral artery stroke. Ann Neurol. 2009;66(3):298-309.
11. Anens E, Kristensen B, Hager-Ross C. Reactive grip force control in persons with cerebellar stroke: effects on ipsilateral and contralateral hand. Exp Brain Res. 2010;203(1):21-30.
12. Archer DB, Kang N, Misra G, Marble S, Patten C, Coombes SA. Visual feedback alters force control and functional activity in the visuomotor network after stroke. Neuroimage Clin. 2018;17:505-17.
13. Barker RN, Brauer SG, Barry BK, Gill TJ, Carson RG. Training-induced modifications of corticospinal reactivity in severely affected stroke survivors. Exp Brain Res. 2012;221(2):211-21.
14. Barlow S, Custead R, Lee J, Hozan M, Greenwood J. Wireless Sensing of Lower Lip and Thumb-Index Finger 'Ramp-and-Hold' Isometric Force Dynamics in a Small Cohort of Unilateral MCA Stroke: Discussion of Preliminary Findings. Sensors (Basel). 2020;20(4).
15. Bashir S, Vernet M, Najib U, Perez J, Alonso-Alonso M, Knobel M, et al. Enhanced motor function and its neurophysiological correlates after navigated low-frequency repetitive transcranial magnetic stimulation over the contralesional motor cortex in stroke. Restor Neurol Neurosci. 2016;34(4):677-89.
16. Bensmail D, Sarfeld AS, Ameli M, Fink GR, Nowak DA. Arbitrary visuomotor mapping in the grip-lift task: dissociation of performance deficits in right and left middle cerebral artery stroke. Neuroscience. 2012;210:128-36.
17. Bestmann S, Swayne O, Blankenburg F, Ruff CC, Teo J, Weiskopf N, et al. The role of contralesional dorsal premotor cortex after stroke as studied with concurrent TMS-fMRI. J Neurosci. 2010;30(36):11926-37.
18. Bhagat NA, Yozbatiran N, Sullivan JL, Paranjape R, Losey C, Hernandez Z, et al. Neural activity modulations and motor recovery following brain-exoskeleton interface mediated stroke rehabilitation. Neuroimage Clin. 2020;28:102502.
19. Bolognini N, Convento S, Banco E, Mattioli F, Tesio L, Vallar G. Improving ideomotor limb apraxia by electrical stimulation of the left posterior parietal cortex. Brain. 2015;138(Pt 2):428-39.
20. Bolognini N, Russo C, Souza Carneiro MI, Nicotra A, Olgiati E, Spandri V, et al. Bi-hemispheric transcranial direct current stimulation for upper-limb hemiparesis in acute stroke: a randomized, double-blind, sham-controlled trial. Eur J Neurol. 2020;27(12):2473-82.
21. Bonstrup M, Schulz R, Schon G, Cheng B, Feldheim J, Thomalla G, et al. Parietofrontal network upregulation after motor stroke. Neuroimage Clin. 2018;18:720-9.
22. Brandauer B, Hermsdorfer J, Geissendorfer T, Schoch B, Gizewski ER, Timmann D. Impaired and preserved aspects of independent finger control in patients with cerebellar damage. J Neurophysiol. 2012;107(4):1080-93.
23. Bu L, Qu J, Zhao L, Zhang Y, Wang Y. A neuroergonomic approach to assessing motor performance in stroke patients using fNIRS and behavioral data. Appl Ergon. 2023;109:103979.

24. Buckingham G, Holler D, Michelakakis EE, Snow JC. Preserved Object Weight Processing after Bilateral Lateral Occipital Complex Lesions. J Cogn Neurosci. 2018;30(11):1683-90.
25. Buetefisch CM, Revill KP, Haut MW, Kowalski GM, Wischnewski M, Pifer M, et al. Abnormally reduced primary motor cortex output is related to impaired hand function in chronic stroke. J Neurophysiol. 2018;120(4):1680-94.
26. Buma FE, van Kordelaar J, Raemaekers M, van Wegen EEH, Ramsey NF, Kwakkel G. Brain activation is related to smoothness of upper limb movements after stroke. Exp Brain Res. 2016;234(7):2077-89.
27. Bundy DT, Souders L, Baranyai K, Leonard L, Schalk G, Coker R, et al. Contralesional Brain-Computer Interface Control of a Powered Exoskeleton for Motor Recovery in Chronic Stroke Survivors. Stroke. 2017;48(7):1908-15.
28. Buxbaum LJ, Johnson-Frey SH, Bartlett-Williams M. Deficient internal models for planning hand-object interactions in apraxia. Neuropsychologia. 2005;43(6):917-29.
29. Calabro RS, Russo M, Naro A, Milardi D, Balletta T, Leo A, et al. Who May Benefit From Armeo Power Treatment? A Neurophysiological Approach to Predict Neurorehabilitation Outcomes. PM R. 2016;8(10):971-8.
30. Cantillo-Negrete J, Carino-Escobar RI, Carrillo-Mora P, Rodriguez-Barragan MA, Hernandez-Arenas C, Quinzanos-Fresnedo J, et al. Brain-Computer Interface Coupled to a Robotic Hand Orthosis for Stroke Patients' Neurorehabilitation: A Crossover Feasibility Study. Front Hum Neurosci. 2021;15:656975.
31. Carey JR, Kimberley TJ, Lewis SM, Auerbach EJ, Dorsey L, Rundquist P, et al. Analysis of fMRI and finger tracking training in subjects with chronic stroke. Brain. 2002;125(Pt 4):773-88.
32. Carey JR, Durfee WK, Bhatt E, Nagpal A, Weinstein SA, Anderson KM, et al. Comparison of finger tracking versus simple movement training via telerehabilitation to alter hand function and cortical reorganization after stroke. Neurorehabil Neural Repair. 2007;21(3):216-32.
33. Carino-Escobar RI, Valdes-Cristerna R, Carrillo-Mora P, Rodriguez-Barragan MA, Hernandez-Arenas C, Quinzanos-Fresnedo J, et al. Prognosis of stroke upper limb recovery with physiological variables using regression tree ensembles. J Neural Eng. 2021;18(4).
34. Chalard A, Amarantini D, Tisseyre J, Marque P, Gasq D. Spastic co-contraction is directly associated with altered cortical beta oscillations after stroke. Clin Neurophysiol. 2020;131(6):1345-53.
35. Chau ACM, Cheung RTF, Jiang X, Au-Yeung P, Li LSW. Acupuncture of Motor-Implicated Acupoints on Subacute Stroke Patients: An fMRI Evaluation Study. Medical Acupuncture. 2009;21(4):233-41.
36. Chen L, Chen Y, Fu WB, Huang DF, Lo WLA. The Effect of Virtual Reality on Motor Anticipation and Hand Function in Patients with Subacute Stroke: A Randomized Trial on Movement-Related Potential. Neural Plast. 2022;2022:7399995.
37. Cheng B, Petersen M, Schulz R, Boenstrup M, Krawinkel L, Gerloff C, et al. White matter degeneration revealed by fiber-specific analysis relates to recovery of hand function after stroke. Hum Brain Mapp. 2021;42(16):5423-32.
38. Cirstea CM, Lee P, Craciunas SC, Choi IY, Burris JE, Nudo RJ. Pre-therapy Neural State of Bilateral Motor and Premotor Cortices Predicts Therapy Gain After Subcortical Stroke: A Pilot Study. Am J Phys Med Rehabil. 2018;97(1):23-33.
39. Classen J, Schnitzler A, Binkofski F, Werhahn KJ, Kim YS, Kessler KR, et al. The motor syndrome associated with exaggerated inhibition within the primary motor cortex of patients with hemiparetic. Brain. 1997;120 (Pt 4):605-19.
40. Cramer SC, Mark A, Barquist K, Nhan H, Stegbauer KC, Price R, et al. Motor cortex activation is preserved in patients with chronic hemiplegic stroke. Ann Neurol. 2002;52(5):607-16.
41. Dafotakis M, Grefkes C, Eickhoff SB, Karbe H, Fink GR, Nowak DA. Effects of rTMS on grip force control following subcortical stroke. Exp Neurol. 2008;211(2):407-12.
42. Dawson J, Pierce D, Dixit A, Kimberley TJ, Robertson M, Tarver B, et al. Safety, Feasibility, and Efficacy of Vagus Nerve Stimulation Paired With Upper-Limb Rehabilitation After Ischemic Stroke. Stroke. 2016;47(1):143-50.
43. de Paiva Silva FP, Freitas SM, Silva PV, Banjai RM, Alouche SR. Ipsilesional arm motor sequence performance after right and left hemisphere damage. J Mot Behav. 2014;46(6):407-14.
44. Delcamp C, Cormier C, Chalard A, Amarantini D, Gasq D. Botulinum toxin combined with rehabilitation decrease corticomuscular coherence in stroke patients. Clin Neurophysiol. 2022;136:49-57.
45. Di Lazzaro V, Rothwell JC, Talelli P, Capone F, Ranieri F, Wallace AC, et al. Inhibitory theta burst stimulation of affected hemisphere in chronic stroke: a proof of principle, sham-controlled study. Neurosci Lett. 2013;553:148-52.
46. Dimyan MA, Harcum S, Ermer E, Boos AF, Conroy SS, Liu F, et al. Baseline Predictors of Response to Repetitive Task Practice in Chronic Stroke. Neurorehabil Neural Repair. 2022;36(7):426-36.
47. Fang Y, Yue GH, Hrovat K, Sahgal V, Daly JJ. Abnormal cognitive planning and movement smoothness control for a complex shoulder/elbow motor task in stroke survivors. J Neurol Sci. 2007;256(1-2):21-9.

48. Fang Y, Daly JJ, Sun J, Hvorat K, Fredrickson E, Pundik S, et al. Functional corticomuscular connection during reaching is weakened following stroke. Clin Neurophysiol. 2009;120(5):994-1002.
49. Fang Y, Daly JJ, Hansley J, Yao WX, Yang Q, Sun J, et al. Hemispheric activation during planning and execution phases in reaching post stroke: a consort study. Medicine (Baltimore). 2015;94(3):e307.
50. Ferreiro de Andrade KN, Conforto AB. Decreased short-interval intracortical inhibition correlates with better pinch strength in patients with stroke and good motor recovery. Brain Stimul. 2018;11(4):772-4.
51. Fisher BE, Boyd L, Winstein CJ. Contralateral cerebellar damage impairs imperative planning but not updating of aimed arm movements in humans. Exp Brain Res. 2006;174(3):453-66.
52. Foltys H, Krings T, Meister IG, Sparing R, Boroojerdi B, Thron A, et al. Motor representation in patients rapidly recovering after stroke: a functional magnetic resonance imaging and transcranial magnetic stimulation study. Clin Neurophysiol. 2003;114(12):2404-15.
53. Frenkel-Toledo S, Bentin S, Perry A, Liebermann DG, Soroker N. Mirror-neuron system recruitment by action observation: effects of focal brain damage on mu suppression. Neuroimage. 2014;87:127-37.
54. Fujii Y, Nakada T. Cortical reorganization in patients with subcortical hemiparesis: neural mechanisms of functional recovery and prognostic implication. J Neurosurg. 2003;98(1):64-73.
55. Gao Y, Su H, Li R, Zhang Y. Synchronous analysis of brain regions based on multi-scale permutation transfer entropy. Comput Biol Med. 2019;109:272-9.
56. Garcea FE, Stoll H, Buxbaum LJ. Reduced competition between tool action neighbors in left hemisphere stroke. Cortex. 2019;120:269-83.
57. Gera G, Freitas SM, Scholz JP. Relationship of diminished interjoint coordination after stroke to hand path consistency. Exp Brain Res. 2016;234(3):741-51.
58. Giacobbe V, Krebs HI, Volpe BT, Pascual-Leone A, Rykman A, Zeiarati G, et al. Transcranial direct current stimulation (tDCS) and robotic practice in chronic stroke: the dimension of timing. NeuroRehabilitation. 2013;33(1):49-56.
59. Groisser BN, Copen WA, Singhal AB, Hirai KK, Schaechter JD. Corticospinal tract diffusion abnormalities early after stroke predict motor outcome. Neurorehabil Neural Repair. 2014;28(8):751-60.
60. Haaland KY, Harrington DL. Hemispheric control of the initial and corrective components of aiming movements. Neuropsychologia. 1989;27(7):961-9.
61. Haaland KY, Harrington DL, Knight RT. Spatial deficits in ideomotor limb apraxia. A kinematic analysis of aiming movements. Brain. 1999;122 (Pt 6):1169-82.
62. Haaland KY, Schaefer SY, Knight RT, Adair J, Magalhaes A, Sadek J, et al. Ipsilesional trajectory control is related to contralesional arm paralysis after left hemisphere damage. Exp Brain Res. 2009;196(2):195-204.
63. Hammerbeck U, Hoad D, Greenwood R, Rothwell JC. The unsolved role of heightened connectivity from the unaffected hemisphere to paretic arm muscles in chronic stroke. Clin Neurophysiol. 2019;130(5):781-8.
64. Hannanu FF, Goundous I, Detante O, Naegele B, Jaillard A. Spatiotemporal patterns of sensorimotor fMRI activity influence hand motor recovery in subacute stroke: A longitudinal task-related fMRI study. Cortex. 2020;129:80-98.
65. Hannanu FF, Naegele B, Hommel M, Krainik A, Detante O, Jaillard A. White matter tract disruption is associated with ipsilateral hand impairment in subacute stroke: a diffusion MRI study. Neuroradiology. 2022;64(8):1605-15.
66. Hara Y, Obayashi S, Tsujiuchi K, Muraoka Y. The effects of electromyography-controlled functional electrical stimulation on upper extremity function and cortical perfusion in stroke patients. Clin Neurophysiol. 2013;124(10):2008-15.
67. Harrington RM, Chan E, Rounds AK, Wutzke CJ, Dromerick AW, Turkeltaub PE, et al. Roles of Lesioned and Nonlesioned Hemispheres in Reaching Performance Poststroke. Neurorehabil Neural Repair. 2020;34(1):61-71.
68. Harris-Love ML, Morton SM, Perez MA, Cohen LG. Mechanisms of short-term training-induced reaching improvement in severely hemiparetic stroke patients: a TMS study. Neurorehabil Neural Repair. 2011;25(5):398-411.
69. Henriques DY, Filippopulos F, Straube A, Eggert T. The cerebellum is not necessary for visually driven recalibration of hand proprioception. Neuropsychologia. 2014;64:195-204.
70. Hensel L, Tscherpel C, Freytag J, Ritter S, Rehme AK, Volz LJ, et al. Connectivity-Related Roles of Contralesional Brain Regions for Motor Performance Early after Stroke. Cereb Cortex. 2021;31(2):993-1007.
71. Hensel L, Lange F, Tscherpel C, Viswanathan S, Freytag J, Volz LJ, et al. Recovered grasping performance after stroke depends on interhemispheric frontoparietal connectivity. Brain. 2023;146(3):1006-20.
72. Hermsdorf F, Fricke C, Stockert A, Classen J, Rumpf JJ. Motor Performance But Neither Motor Learning Nor Motor Consolidation Are Impaired in Chronic Cerebellar Stroke Patients. Cerebellum. 2020;19(2):275-85.
73. Hermsdorfer J, Blankenfeld H, Goldenberg G. The dependence of ipsilesional aiming deficits on task demands, lesioned hemisphere, and apraxia. Neuropsychologia. 2003;41(12):1628-43.

74. Hermsdorfer J, Li Y, Randerath J, Roby-Brami A, Goldenberg G. Tool use kinematics across different modes of execution. Implications for action representation and apraxia. Cortex. 2013;49(1):184-99.
75. Hirai KK, Groisser BN, Copen WA, Singhal AB, Schaechter JD. Comparing prognostic strength of acute corticospinal tract injury measured by a new diffusion tensor imaging based template approach versus common approaches. J Neurosci Methods. 2016;257:204-13.
76. Hordacre B, Ghosh R, Goldsworthy MR, Ridding MC. Transcranial Magnetic Stimulation-EEG Biomarkers of Poststroke Upper-Limb Motor Function. J Stroke Cerebrovasc Dis. 2019;28(12):104452.
77. Hordacre B, Goldsworthy MR, Welsby E, Graetz L, Ballinger S, Hillier S. Resting State Functional Connectivity Is Associated With Motor Pathway Integrity and Upper-Limb Behavior in Chronic Stroke. Neurorehabil Neural Repair. 2020;34(6):547-57.
78. Hosomi K, Morris S, Sakamoto T, Taguchi J, Maruo T, Kageyama Y, et al. Daily Repetitive Transcranial Magnetic Stimulation for Poststroke Upper Limb Paresis in the Subacute Period. J Stroke Cerebrovasc Dis. 2016;25(7):1655-64.
79. Hsu YF, Huang YZ, Lin YY, Tang CW, Liao KK, Lee PL, et al. Intermittent theta burst stimulation over ipsilesional primary motor cortex of subacute ischemic stroke patients: a pilot study. Brain Stimul. 2013;6(2):166-74.
80. Hummel FC, Voller B, Celnik P, Floel A, Giraux P, Gerloff C, et al. Effects of brain polarization on reaction times and pinch force in chronic stroke. BMC Neurosci. 2006;7:73.
81. Hummel FC, Steven B, Hoppe J, Heise K, Thomalla G, Cohen LG, et al. Deficient intracortical inhibition (SICI) during movement preparation after chronic stroke. Neurology. 2009;72(20):1766-72.
82. Ilic NV, Dubljanin-Raspopovic E, Nedeljkovic U, Tomanovic-Vujadinovic S, Milanovic SD, Petronic-Markovic I, et al. Effects of anodal tDCS and occupational therapy on fine motor skill deficits in patients with chronic stroke. Restor Neurol Neurosci. 2016;34(6):935-45.
83. Jacobs S, Bussel B, Combeaud M, Roby-Brami A. The use of a tool requires its incorporation into the movement: evidence from stick-pointing in apraxia. Cortex. 2009;45(4):444-55.
84. Jang SH, Jang WH. Change of the Corticospinal Tract in the Unaffected Hemisphere by Change of the Dominant Hand Following Stroke: A Cohort Study. Medicine (Baltimore). 2016;95(6):e2620.
85. Jian C, Deng L, Liu H, Yan T, Wang X, Song R. Modulating and restoring inter-muscular coordination in stroke patients using two-dimensional myoelectric computer interface: a cross-sectional and longitudinal study. J Neural Eng. 2021;18(3).
86. Jochumsen M, Niazi IK, Taylor D, Farina D, Dremstrup K. Detecting and classifying movement-related cortical potentials associated with hand movements in healthy subjects and stroke patients from single-electrode, single-trial EEG. J Neural Eng. 2015;12(5):056013.
87. Johansen-Berg H, Rushworth MF, Bogdanovic MD, Kischka U, Wimalaratna S, Matthews PM. The role of ipsilateral premotor cortex in hand movement after stroke. Proc Natl Acad Sci U S A. 2002;99(22):14518-23.
88. Kang N, Cauraugh JH. Right Hemisphere Contributions to Bilateral Force Control in Chronic Stroke: A Preliminary Report. J Stroke Cerebrovasc Dis. 2018;27(11):3218-23.
89. Kantak S, McGrath R, Zahedi N, Luchmee D. Behavioral and neurophysiological mechanisms underlying motor skill learning in patients with post-stroke hemiparesis. Clin Neurophysiol. 2018;129(1):1-12.
90. Karnath HO, Perenin MT. Cortical control of visually guided reaching: evidence from patients with optic ataxia. Cereb Cortex. 2005;15(10):1561-9.
91. Kemlin C, Moulton E, Lamy JC, Houot M, Valabregue R, Leder S, et al. Elucidating the Structural and Functional Correlates of Upper-Limb Poststroke Motor Impairment. Stroke. 2019;50(12):3647-9.
92. Kemppainen P, Waltimo A, Palomaki H, Salonen O, Kononen M, Kaste M. Masticatory force and function in patients with hemispheric brain infarction and hemiplegia. J Dent Res. 1999;78(12):1810-4.
93. Kim YH, You SH, Ko MH, Park JW, Lee KH, Jang SH, et al. Repetitive transcranial magnetic stimulation-induced corticomotor excitability and associated motor skill acquisition in chronic stroke. Stroke. 2006;37(6):1471-6.
94. Kim J, Yim J. Effects of High-Frequency Repetitive Transcranial Magnetic Stimulation Combined with Task-Oriented Mirror Therapy Training on Hand Rehabilitation of Acute Stroke Patients. Med Sci Monit. 2018;24:743-50.
95. Kim H, Yoo EY, Jung MY, Kim J, Park JH, Kang DH. The effects of mental practice combined with modified constraint-induced therapy on corticospinal excitability, movement quality, function, and activities of daily living in persons with stroke. Disabil Rehabil. 2018;40(20):2449-57.
96. Kimberley TJ, Lewis SM, Auerbach EJ, Dorsey LL, Lojovich JM, Carey JR. Electrical stimulation driving functional improvements and cortical changes in subjects with stroke. Exp Brain Res. 2004;154(4):450-60.
97. Koganemaru S, Mima T, Thabit MN, Ikkaku T, Shimada K, Kanematsu M, et al. Recovery of upper-limb function due to enhanced use-dependent plasticity in chronic stroke patients. Brain. 2010;133(11):3373-84.
98. Konczak J, Pierscianek D, Hirsiger S, Bultmann U, Schoch B, Gizewski ER, et al. Recovery of upper limb function after cerebellar stroke: lesion symptom mapping and arm kinematics. Stroke. 2010;41(10):2191-200.

99. Koob JL, Viswanathan S, Mustin M, Mallick I, Krick S, Fink GR, et al. To engage or not engage: Early incentive motivation prevents symptoms of chronic post-stroke depression - A longitudinal study. Neuroimage Clin. 2023;37:103360.
100. Kou N, Park CH, Seghier ML, Leff AP, Ward NS. Can fully automated detection of corticospinal tract damage be used in stroke patients? Neurology. 2013;80(24):2242-5.
101. Kuper M, Brandauer B, Thurling M, Schoch B, Gizewski ER, Timmann D, et al. Impaired prehension is associated with lesions of the superior and inferior hand representation within the human cerebellum. J Neurophysiol. 2011;105(5):2018-29.
102. Kuppuswamy A, Clark EV, Turner IF, Rothwell JC, Ward NS. Post-stroke fatigue: a deficit in corticomotor excitability? Brain. 2015;138(Pt 1):136-48.
103. Kwon YH, Lee MY, Park JW, Kang JH, Yang DS, Kim YH, et al. Differences of cortical activation pattern between cortical and corona radiata infarct. Neurosci Lett. 2007;417(2):138-42.
104. Kwon TG, Kim YH, Chang WH, Bang OY, Shin YI. Effective method of combining rTMS and motor training in stroke patients. Restor Neurol Neurosci. 2014;32(2):223-32.
105. Lafe CW, Liu F, Simpson TW, Moon CH, Collinger JL, Wittenberg GF, et al. Force oscillations underlying precision grip in humans with lesioned corticospinal tracts. Neuroimage Clin. 2023;38:103398.
106. Lai CJ, Wang CP, Tsai PY, Chan RC, Lin SH, Lin FG, et al. Corticospinal integrity and motor impairment predict outcomes after excitatory repetitive transcranial magnetic stimulation: a preliminary study. Arch Phys Med Rehabil. 2015;96(1):69-75.
107. Lang CE, Wagner JM, Bastian AJ, Hu Q, Edwards DF, Sahrmann SA, et al. Deficits in grasp versus reach during acute hemiparesis. Exp Brain Res. 2005;166(1):126-36.
108. Lariviere S, Ward NS, Boudrias MH. Disrupted functional network integrity and flexibility after stroke: Relation to motor impairments. Neuroimage Clin. 2018;19:883-91.
109. Larsen LH, Zibrandtsen IC, Wienecke T, Kjaer TW, Christensen MS, Nielsen JB, et al. Corticomuscular coherence in the acute and subacute phase after stroke. Clin Neurophysiol. 2017;128(11):2217-26.
110. Larsen LH, Zibrandtsen IC, Wienecke T, Kjaer TW, Langberg H, Nielsen JB, et al. Modulation of task-related cortical connectivity in the acute and subacute phase after stroke. Eur J Neurosci. 2018;47(8):1024-32.
111. Lefebvre S, Dricot L, Laloux P, Gradkowski W, Desfontaines P, Evrard F, et al. Neural substrates underlying stimulation-enhanced motor skill learning after stroke. Brain. 2015;138(Pt 1):149-63.
112. Lewis GN, Perreault EJ. Side of lesion influences bilateral activation in chronic, post-stroke hemiparesis. Clin Neurophysiol. 2007;118(9):2050-62.
113. Liepert J, Restemeyer C, Munchau A, Weiller C. Motor cortex excitability after thalamic infarction. Clin Neurophysiol. 2005;116(7):1621-7.
114. Liepert J, Zittel S, Weiller C. Improvement of dexterity by single session low-frequency repetitive transcranial magnetic stimulation over the contralesional motor cortex in acute stroke: a double-blind placebo-controlled crossover trial. Restor Neurol Neurosci. 2007;25(5-6):461-5.
115. Lindberg PG, Skejo PH, Rounis E, Nagy Z, Schmitz C, Wernegren H, et al. Wallerian degeneration of the corticofugal tracts in chronic stroke: a pilot study relating diffusion tensor imaging, transcranial magnetic stimulation, and hand function. Neurorehabil Neural Repair. 2007;21(6):551-60.
116. Lindberg PG, Gaverth J, Fagergren A, Fransson P, Forssberg H, Borg J. Cortical activity in relation to velocity dependent movement resistance in the flexor muscles of the hand after stroke. Neurorehabil Neural Repair. 2009;23(8):800-10.
117. Liu H, Au-Yeung SSY. Corticomotor Excitability Effects of Peripheral Nerve Electrical Stimulation to the Paretic Arm in Stroke. Am J Phys Med Rehabil. 2017;96(10):687-93.
118. Lopez-Larraz E, Figueiredo TC, Insausti-Delgado A, Ziemann U, Birbaumer N, Ramos-Murguialday A. Event-related desynchronization during movement attempt and execution in severely paralyzed stroke patients: An artifact removal relevance analysis. Neuroimage Clin. 2018;20:972-86.
119. Lotze M, Markert J, Sauseng P, Hoppe J, Plewnia C, Gerloff C. The role of multiple contralesional motor areas for complex hand movements after internal capsular lesion. J Neurosci. 2006;26(22):6096-102.
120. Lotze M, Beutling W, Loibl M, Domin M, Platz T, Schminke U, et al. Contralesional motor cortex activation depends on ipsilesional corticospinal tract integrity in well-recovered subcortical stroke patients. Neurorehabil Neural Repair. 2012;26(6):594-603.
121. Lu RR, Zheng MX, Li J, Gao TH, Hua XY, Liu G, et al. Motor imagery based brain-computer interface control of continuous passive motion for wrist extension recovery in chronic stroke patients. Neurosci Lett. 2020;718:134727.
122. Luft AR, McCombe-Waller S, Whitall J, Forrester LW, Macko R, Sorkin JD, et al. Repetitive bilateral arm training and motor cortex activation in chronic stroke: a randomized controlled trial. JAMA. 2004;292(15):1853-61.

123. Mani S, Mutha PK, Przybyla A, Haaland KY, Good DC, Sainburg RL. Contralesional motor deficits after unilateral stroke reflect hemisphere-specific control mechanisms. Brain. 2013;136(Pt 4):1288-303.
124. Mansur CG, Fregni F, Boggio PS, Riberto M, Gallucci-Neto J, Santos CM, et al. A sham stimulation-controlled trial of rTMS of the unaffected hemisphere in stroke patients. Neurology. 2005;64(10):1802-4.
125. Matarasso AK, Rieke JD, White K, Yusufali MM, Daly JJ. Combined real-time fMRI and real time fNIRS brain computer interface (BCI): Training of volitional wrist extension after stroke, a case series pilot study. PLoS One. 2021;16(5):e0250431.
126. Matsuura A, Onoda K, Oguro H, Yamaguchi S. Magnetic stimulation and movement-related cortical activity for acute stroke with hemiparesis. Eur J Neurol. 2015;22(12):1526-32.
127. McKenzie A, Dodakian L, See J, Le V, Quinlan EB, Bridgford C, et al. Validity of Robot-Based Assessments of Upper Extremity Function. Arch Phys Med Rehabil. 2017;98(10):1969-76 e2.
128. McPherson JG, Chen A, Ellis MD, Yao J, Heckman CJ, Dewald JPA. Progressive recruitment of contralesional cortico-reticulospinal pathways drives motor impairment post stroke. J Physiol. 2018;596(7):1211-25.
129. Miller KJ, Gallina A, Neva JL, Ivanova TD, Snow NJ, Ledwell NM, et al. Effect of repetitive transcranial magnetic stimulation combined with robot-assisted training on wrist muscle activation post-stroke. Clin Neurophysiol. 2019;130(8):1271-9.
130. Mutha PK, Stapp LH, Sainburg RL, Haaland KY. Frontal and parietal cortex contributions to action modification. Cortex. 2014;57:38-50.
131. Naros G, Gharabaghi A. Physiological and behavioral effects of beta-tACS on brain self-regulation in chronic stroke. Brain Stimul. 2017;10(2):251-9.
132. Newton JM, Ward NS, Parker GJ, Deichmann R, Alexander DC, Friston KJ, et al. Non-invasive mapping of corticofugal fibres from multiple motor areas--relevance to stroke recovery. Brain. 2006;129(Pt 7):1844-58.
133. Nicolo P, Magnin C, Pedrazzini E, Plomp G, Mottaz A, Schnider A, et al. Comparison of Neuroplastic Responses to Cathodal Transcranial Direct Current Stimulation and Continuous Theta Burst Stimulation in Subacute Stroke. Arch Phys Med Rehabil. 2018;99(5):862-72 e1.
134. Noh JS, Lim JH, Choi TW, Jang SG, Pyun SB. Effects and safety of combined rTMS and action observation for recovery of function in the upper extremities in stroke patients: A randomized controlled trial. Restor Neurol Neurosci. 2019;37(3):219-30.
135. Nowak DA, Grefkes C, Dafotakis M, Eickhoff S, Kust J, Karbe H, et al. Effects of low-frequency repetitive transcranial magnetic stimulation of the contralesional primary motor cortex on movement kinematics and neural activity in subcortical stroke. Arch Neurol. 2008;65(6):741-7.
136. O'Shea J, Boudrias MH, Stagg CJ, Bachtiar V, Kischka U, Blicher JU, et al. Predicting behavioural response to TDCS in chronic motor stroke. Neuroimage. 2014;85 Pt 3(Pt 3):924-33.
137. Ouin E, Roussel M, Aarabi A, Arnoux A, Tasseel-Ponche S, Andriuta D, et al. Poststroke action slowing: Motor and attentional impairments and their imaging determinants. Evidence from lesion-symptom mapping, disconnection and fMRI activation studies. Neuropsychologia. 2022;177:108401.
138. Palmer JA, Wheaton LA, Gray WA, Saltao da Silva MA, Wolf SL, Borich MR. Role of Interhemispheric Cortical Interactions in Poststroke Motor Function. Neurorehabil Neural Repair. 2019;33(9):762-74.
139. Pan LH, Yang WW, Kao CL, Tsai MW, Wei SH, Fregni F, et al. Effects of 8-week sensory electrical stimulation combined with motor training on EEG-EMG coherence and motor function in individuals with stroke. Sci Rep. 2018;8(1):9217.
140. Park W, Kwon GH, Kim DH, Kim YH, Kim SP, Kim L. Assessment of cognitive engagement in stroke patients from single-trial EEG during motor rehabilitation. IEEE Trans Neural Syst Rehabil Eng. 2015;23(3):351-62.
141. Pennati GV, Plantin J, Carment L, Roca P, Baron JC, Pavlova E, et al. Recovery and Prediction of Dynamic Precision Grip Force Control After Stroke. Stroke. 2020;51(3):944-51.
142. Pierella C, Pirondini E, Kinany N, Coscia M, Giang C, Miehlbradt J, et al. A multimodal approach to capture post-stroke temporal dynamics of recovery. J Neural Eng. 2020;17(4):045002.
143. Piscitelli D, Turpin NA, Subramanian SK, Feldman AG, Levin MF. Deficits in corticospinal control of stretch reflex thresholds in stroke: Implications for motor impairment. Clin Neurophysiol. 2020;131(9):2067-78.
144. Pohl PS, Luchies CW, Stoker-Yates J, Duncan PW. Upper extremity control in adults post stroke with mild residual impairment. Neurorehabil Neural Repair. 2000;14(1):33-41.
145. Pohl PS, Filion DL, Kim SH. Effects of practice and unpredictable distractors on planning and executing aiming after stroke. Neurorehabil Neural Repair. 2003;17(2):93-100.
146. Quandt F, Bonstrup M, Schulz R, Timmermann JE, Mund M, Wessel MJ, et al. The functional role of beta-oscillations in the supplementary motor area during reaching and grasping after stroke: A question of structural damage to the corticospinal tract. Hum Brain Mapp. 2019;40(10):3091-101.

147. Ramos-Murguialday A, Broetz D, Rea M, Laer L, Yilmaz O, Brasil FL, et al. Brain-machine interface in chronic stroke rehabilitation: a controlled study. Ann Neurol. 2013;74(1):100-8.
148. Rehme AK, Fink GR, von Cramon DY, Grefkes C. The role of the contralesional motor cortex for motor recovery in the early days after stroke assessed with longitudinal FMRI. Cereb Cortex. 2011;21(4):756-68.
149. Rehme AK, Eickhoff SB, Wang LE, Fink GR, Grefkes C. Dynamic causal modeling of cortical activity from the acute to the chronic stage after stroke. Neuroimage. 2011;55(3):1147-58.
150. Rehme AK, Volz LJ, Feis DL, Eickhoff SB, Fink GR, Grefkes C. Individual prediction of chronic motor outcome in the acute post-stroke stage: Behavioral parameters versus functional imaging. Hum Brain Mapp. 2015;36(11):4553-65.
151. Remsik AB, Williams L, Jr., Gjini K, Dodd K, Thoma J, Jacobson T, et al. Ipsilesional Mu Rhythm Desynchronization and Changes in Motor Behavior Following Post Stroke BCI Intervention for Motor Rehabilitation. Front Neurosci. 2019;13:53.
152. Remsik AB, Gjini K, Williams L, Jr., van Kan PLE, Gloe S, Bjorklund E, et al. Ipsilesional Mu Rhythm Desynchronization Correlates With Improvements in Affected Hand Grip Strength and Functional Connectivity in Sensorimotor Cortices Following BCI-FES Intervention for Upper Extremity in Stroke Survivors. Front Hum Neurosci. 2021;15:725645.
153. Renner CI, Woldag H, Atanasova R, Hummelsheim H. Change of facilitation during voluntary bilateral hand activation after stroke. J Neurol Sci. 2005;239(1):25-30.
154. Riga A, Gathy E, Ghinet M, De Laet C, Bihin B, Regnier M, et al. Evidence of Motor Skill Learning in Acute Stroke Patients Without Lesions to the Thalamus and Internal Capsule. Stroke. 2022;53(7):2361-8.
155. Rimmele DL, Frey BM, Cheng B, Schulz R, Krawinkel LA, Bonstrup M, et al. Association of Extrapyramidal Tracts' Integrity With Performance in Fine Motor Skills After Stroke. Stroke. 2018;49(12):2928-32.
156. Rondina JM, Park CH, Ward NS. Brain regions important for recovery after severe post-stroke upper limb paresis. J Neurol Neurosurg Psychiatry. 2017;88(9):737-43.
157. Rosso C, Valabregue R, Attal Y, Vargas P, Gaudron M, Baronnet F, et al. Contribution of corticospinal tract and functional connectivity in hand motor impairment after stroke. PLoS One. 2013;8(9):e73164.
158. Rosso C, Moulton EJ, Kemlin C, Leder S, Corvol JC, Mehdi S, et al. Cerebello-Motor Paired Associative Stimulation and Motor Recovery in Stroke: a Randomized, Sham-Controlled, Double-Blind Pilot Trial. Neurotherapeutics. 2022;19(2):491-500.
159. Rungseethanakul S, Tretriluxana J, Piriyaprasarth P, Pakaprot N, Jitaree K, Tretriluxana S, et al. Task Oriented Training Activities Post Stroke Will Produce Measurable Alterations in Brain Plasticity Concurrent with Skill Improvement. Top Stroke Rehabil. 2022;29(4):241-54.
160. Sakurada T, Nakajima T, Morita M, Hirai M, Watanabe E. Improved motor performance in patients with acute stroke using the optimal individual attentional strategy. Sci Rep. 2017;7:40592.
161. Saleh S, Adamovich SV, Tunik E. Mirrored feedback in chronic stroke: recruitment and effective connectivity of ipsilesional sensorimotor networks. Neurorehabil Neural Repair. 2014;28(4):344-54.
162. Salehi Dehno N, Kamali F, Shariat A, Jaberzadeh S. Comparison of Transcallosal Inhibition Between Hemispheres and Its Relationship with Motor Behavior in Patients with Severe Upper Extremity Impairment After Subacute Stroke. J Stroke Cerebrovasc Dis. 2022;31(6):106469.
163. Sawaki L, Butler AJ, Leng X, Wassenaar PA, Mohammad YM, Blanton S, et al. Constraint-induced movement therapy results in increased motor map area in subjects 3 to 9 months after stroke. Neurorehabil Neural Repair. 2008;22(5):505-13.
164. Schaechter JD, Perdue KL. Enhanced cortical activation in the contralesional hemisphere of chronic stroke patients in response to motor skill challenge. Cereb Cortex. 2008;18(3):638-47.
165. Schaefer SY, Haaland KY, Sainburg RL. Ipsilesional motor deficits following stroke reflect hemispheric specializations for movement control. Brain. 2007;130(Pt 8):2146-58.
166. Schaefer SY, Haaland KY, Sainburg RL. Hemispheric specialization and functional impact of ipsilesional deficits in movement coordination and accuracy. Neuropsychologia. 2009;47(13):2953-66.
167. Schaffer JE, Maenza C, Good DC, Przybyla A, Sainburg RL. Left hemisphere damage produces deficits in predictive control of bilateral coordination. Exp Brain Res. 2020;238(12):2733-44.
168. Schranz C, Srivastava S, Seamon BA, Marebwa B, Bonilha L, Ramakrishnan V, et al. Different aspects of hand grip performance associated with structural connectivity of distinct sensorimotor networks in chronic stroke. Physiol Rep. 2023;11(7):e15659.
169. Schulz R, Park CH, Boudrias MH, Gerloff C, Hummel FC, Ward NS. Assessing the integrity of corticospinal pathways from primary and secondary cortical motor areas after stroke. Stroke. 2012;43(8):2248-51.
170. Schulz R, Koch P, Zimerman M, Wessel M, Bonstrup M, Thomalla G, et al. Parietofrontal motor pathways and their association with motor function after stroke. Brain. 2015;138(Pt 7):1949-60.

171. Semrau JA, Herter TM, Kenzie JM, Findlater SE, Scott SH, Dukelow SP. Robotic Characterization of Ipsilesional Motor Function in Subacute Stroke. Neurorehabil Neural Repair. 2017;31(6):571-82.
172. Shang X, Meng X, Xiao X, Xie Z, Yuan X. Grip training improves handgrip strength, cognition, and brain white matter in minor acute ischemic stroke patients. Clin Neurol Neurosurg. 2021;209:106886.
173. Shin HK, Cho SH, Jeon HS, Lee YH, Song JC, Jang SH, et al. Cortical effect and functional recovery by the electromyography-triggered neuromuscular stimulation in chronic stroke patients. Neurosci Lett. 2008;442(3):174-9.
174. Small SL, Hlustik P, Noll DC, Genovese C, Solodkin A. Cerebellar hemispheric activation ipsilateral to the paretic hand correlates with functional recovery after stroke. Brain. 2002;125(Pt 7):1544-57.
175. Stagg CJ, Bachtiar V, O'Shea J, Allman C, Bosnell RA, Kischka U, et al. Cortical activation changes underlying stimulation-induced behavioural gains in chronic stroke. Brain. 2012;135(Pt 1):276-84.
176. Stewart JC, Gordon J, Winstein CJ. Control of reach extent with the paretic and nonparetic arms after unilateral sensorimotor stroke: kinematic differences based on side of brain damage. Exp Brain Res. 2014;232(7):2407-19.
177. Stewart JC, O'Donnell M, Handlery K, Winstein CJ. Skilled Reach Performance Correlates With Corpus Callosum Structural Integrity in Individuals With Mild Motor Impairment After Stroke: A Preliminary Investigation. Neurorehabil Neural Repair. 2017;31(7):657-65.
178. Stewart JC, Baird JF, Lewis AF, Fritz SL, Fridriksson J. Effect of behavioural practice targeted at the motor action selection network after stroke. Eur J Neurosci. 2022;56(4):4469-85.
179. Stinear CM, Barber PA, Smale PR, Coxon JP, Fleming MK, Byblow WD. Functional potential in chronic stroke patients depends on corticospinal tract integrity. Brain. 2007;130(Pt 1):170-80.
180. Strens LH, Asselman P, Pogosyan A, Loukas C, Thompson AJ, Brown P. Corticocortical coupling in chronic stroke: its relevance to recovery. Neurology. 2004;63(3):475-84.
181. Sugarman H, Avni A, Nathan R, Weisel-Eichler A, Tiran J. Movement in the ipsilesional hand is segmented following unilateral brain damage. Brain and Cognition. 2002;48(2-3):579-87.
182. Summers JJ, Kagerer FA, Garry MI, Hiraga CY, Loftus A, Cauraugh JH. Bilateral and unilateral movement training on upper limb function in chronic stroke patients: A TMS study. J Neurol Sci. 2007;252(1):76-82.
183. Sun Y, Ledwell NMH, Boyd LA, Zehr EP. Unilateral wrist extension training after stroke improves strength and neural plasticity in both arms. Exp Brain Res. 2018;236(7):2009-21.
184. Szameitat AJ, Shen S, Conforto A, Sterr A. Cortical activation during executed, imagined, observed, and passive wrist movements in healthy volunteers and stroke patients. Neuroimage. 2012;62(1):266-80.
185. Taga M, Charalambous CC, Raju S, Lin J, Zhang Y, Stern E, et al. Corticoreticulospinal tract neurophysiology in an arm and hand muscle in healthy and stroke subjects. J Physiol. 2021;599(16):3955-71.
186. Takahashi CD, Der-Yeghiaian L, Le V, Motiwala RR, Cramer SC. Robot-based hand motor therapy after stroke. Brain. 2008;131(Pt 2):425-37.
187. Takeuchi N, Tada T, Toshima M, Chuma T, Matsuo Y, Ikoma K. Inhibition of the unaffected motor cortex by 1 Hz repetitive transcranical magnetic stimulation enhances motor performance and training effect of the paretic hand in patients with chronic stroke. J Rehabil Med. 2008;40(4):298-303.
188. Takeuchi N, Chuma T, Matsuo Y, Watanabe I, Ikoma K. Repetitive transcranial magnetic stimulation of contralesional primary motor cortex improves hand function after stroke. Stroke. 2005;36(12):2681-6.
189. Takeuchi N, Tada T, Chuma T, Matsuo Y, Ikoma K. Disinhibition of the premotor cortex contributes to a maladaptive change in the affected hand after stroke. Stroke. 2007;38(5):1551-6.
190. Taylor L, Lewis GN, Taylor D. Short-term effects of electrical stimulation and voluntary activity on corticomotor excitability in healthy individuals and people with stroke. J Clin Neurophysiol. 2012;29(3):237-43.
191. Thickbroom GW, Byrnes ML, Archer SA, Mastaglia FL. Motor outcome after subcortical stroke: MEPs correlate with hand strength but not dexterity. Clin Neurophysiol. 2002;113(12):2025-9.
192. Thickbroom GW, Byrnes ML, Archer SA, Mastaglia FL. Motor outcome after subcortical stroke correlates with the degree of cortical reorganization. Clin Neurophysiol. 2004;115(9):2144-50.
193. Tian R, Dewald JPA, Yang Y. Assessing the Usage of Indirect Motor Pathways Following a Hemiparetic Stroke. IEEE Trans Neural Syst Rehabil Eng. 2021;29:1568-72.
194. Tilsley PA, Romaiguere P, Tramoni E, Felician O, Sarlegna FR. Interlimb Transfer of Reach Adaptation Does Not Require an Intact Corpus Callosum: Evidence from Patients with Callosal Lesions and Agenesis. eNeuro. 2021;8(4).
195. Tombari D, Loubinoux I, Pariente J, Gerdelat A, Albucher JF, Tardy J, et al. A longitudinal fMRI study: in recovering and then in clinically stable sub-cortical stroke patients. Neuroimage. 2004;23(3):827-39.
196. Tscherpel C, Hensel L, Lemberg K, Vollmer M, Volz LJ, Fink GR, et al. The differential roles of contralesional frontoparietal areas in cortical reorganization after stroke. Brain Stimul. 2020;13(3):614-24.

197. Turolla A, Daud Albasini OA, Oboe R, Agostini M, Tonin P, Paolucci S, et al. Haptic-based neurorehabilitation in poststroke patients: a feasibility prospective multicentre trial for robotics hand rehabilitation. Comput Math Methods Med. 2013;2013:895492.
198. Urbin MA, Collinger JL, Wittenberg GF. Corticospinal recruitment of spinal motor neurons in human stroke survivors. J Physiol. 2021;599(18):4357-73.
199. Vahdat S, Darainy M, Thiel A, Ostry DJ. A Single Session of Robot-Controlled Proprioceptive Training Modulates Functional Connectivity of Sensory Motor Networks and Improves Reaching Accuracy in Chronic Stroke. Neurorehabil Neural Repair. 2019;33(1):70-81.
200. van Assche M, Dirren E, Bourgeois A, Kleinschmidt A, Richiardi J, Carrera E. Periinfarct rewiring supports recovery after primary motor cortex stroke. J Cereb Blood Flow Metab. 2021;41(9):2174-84.
201. van Dokkum LEH, le Bars E, Mottet D, Bonafe A, Menjot de Champfleur N, Laffont I. Modified Brain Activations of the Nondamaged Hemisphere During Ipsilesional Upper-Limb Movement in Persons With Initial Severe Motor Deficits Poststroke. Neurorehabil Neural Repair. 2018;32(1):34-45.
202. Velicki MR, Winstein CJ, Pohl PS. Impaired direction and extent specification of aimed arm movements in humans with stroke-related brain damage. Exp Brain Res. 2000;130(3):362-74.
203. Verleger R, Adam S, Rose M, Vollmer C, Wauschkuhn B, Kompf D. Control of hand movements after striatocapsular stroke: high-resolution temporal analysis of the function of ipsilateral activation. Clin Neurophysiol. 2003;114(8):1468-76.
204. Volz LJ, Rehme AK, Michely J, Nettekoven C, Eickhoff SB, Fink GR, et al. Shaping Early Reorganization of Neural Networks Promotes Motor Function after Stroke. Cereb Cortex. 2016;26(6):2882-94.
205. Volz LJ, Vollmer M, Michely J, Fink GR, Rothwell JC, Grefkes C. Time-dependent functional role of the contralesional motor cortex after stroke. Neuroimage Clin. 2017;16:165-74.
206. Vongvaivanichakul PT, J.; Bovonsunthonchai, S.; Pakaprot, N.; Laksanakorn, W. Reach-to-grasp training in individuals with chronic stroke augmented by low-frequency repetitive transcranial magnetic stimulation. Journal of the Medical Association of Thailand. 2014.
207. Wang LE, Fink GR, Diekhoff S, Rehme AK, Eickhoff SB, Grefkes C. Noradrenergic enhancement improves motor network connectivity in stroke patients. Ann Neurol. 2011;69(2):375-88.
208. Ward NS, Brown MM, Thompson AJ, Frackowiak RS. Neural correlates of outcome after stroke: a cross-sectional fMRI study. Brain. 2003;126(Pt 6):1430-48.
209. Ward NS, Brown MM, Thompson AJ, Frackowiak RS. Neural correlates of motor recovery after stroke: a longitudinal fMRI study. Brain. 2003;126(Pt 11):2476-96.
210. Ward NS, Newton JM, Swayne OB, Lee L, Frackowiak RS, Thompson AJ, et al. The relationship between brain activity and peak grip force is modulated by corticospinal system integrity after subcortical stroke. Eur J Neurosci. 2007;25(6):1865-73.
211. Watanabe K, Kudo Y, Sugawara E, Nakamizo T, Amari K, Takahashi K, et al. Comparative study of ipsilesional and contralesional repetitive transcranial magnetic stimulations for acute infarction. J Neurol Sci. 2018;384:10-4.
212. Weitnauer L, Frisch S, Melie-Garcia L, Preisig M, Schroeter ML, Sajfutdinow I, et al. Mapping grip force to motor networks. Neuroimage. 2021;229:117735.
213. Weller P, Wittsack HJ, Siebler M, Homberg V, Seitz RJ. Motor recovery as assessed with isometric finger movements and perfusion magnetic resonance imaging after acute ischemic stroke. Neurorehabil Neural Repair. 2006;20(3):390-7.
214. Wenzelburger R, Kopper F, Frenzel A, Stolze H, Klebe S, Brossmann A, et al. Hand coordination following capsular stroke. Brain. 2005;128(Pt 1):64-74.
215. Whitall J, Waller SM, Sorkin JD, Forrester LW, Macko RF, Hanley DF, et al. Bilateral and unilateral arm training improve motor function through differing neuroplastic mechanisms: a single-blinded randomized controlled trial. Neurorehabil Neural Repair. 2011;25(2):118-29.
216. Wiese H, Stude P, Sarge R, Nebel K, Diener HC, Keidel M. Reorganization of motor execution rather than preparation in poststroke hemiparesis. Stroke. 2005;36(7):1474-9.
217. Wilkins KB, Yao J, Owen M, Karbasforoushan H, Carmona C, Dewald JPA. Limited capacity for ipsilateral secondary motor areas to support hand function post-stroke. J Physiol. 2020;598(11):2153-67.
218. Winstein CJ, Pohl PS. Effects of unilateral brain damage on the control of goal-directed hand movements. Exp Brain Res. 1995;105(1):163-74.
219. Wittmann M, von Steinbuchel N, Szelag E. Hemispheric specialisation for self-paced motor sequences. Brain Res Cogn Brain Res. 2001;10(3):341-4.
220. Wolbrecht ET, Rowe JB, Chan V, Ingemanson ML, Cramer SC, Reinkensmeyer DJ. Finger strength, individuation, and their interaction: Relationship to hand function and corticospinal tract injury after stroke. Clin Neurophysiol. 2018;129(4):797-808.

221. Woldag H, Renner C, Hummelsheim H. Isotonic and isometric contractions exert the same amount of corticomotor system excitability in healthy subjects and patients after stroke. J Rehabil Med. 2008;40(2):107-11.
222. Woldag H, Lukhaup S, Renner C, Hummelsheim H. Enhanced motor cortex excitability during ipsilateral voluntary hand activation in healthy subjects and stroke patients. Stroke. 2004;35(11):2556-9.
223. Xi X, Ding J, Wang J, Zhao YB, Wang T, Kong W, et al. Analysis of Functional Corticomuscular Coupling Based on Multiscale Transfer Spectral Entropy. IEEE J Biomed Health Inform. 2022;26(10):5085-96.
224. Zemke AC, Heagerty PJ, Lee C, Cramer SC. Motor cortex organization after stroke is related to side of stroke and level of recovery. Stroke. 2003;34(5):e23-8.
225. Zhang JJ, Bai Z, Fong KNK. Priming Intermittent Theta Burst Stimulation for Hemiparetic Upper Limb After Stroke: A Randomized Controlled Trial. Stroke. 2022;53(7):2171-81.
